# A two-sample Mendelian randomization study of type 1 diabetes and the risk of 22 site-specific cancers

**DOI:** 10.1038/s41598-025-89288-3

**Published:** 2025-04-03

**Authors:** Mikiyas Amare Getu, Xianbin Zhang, Ying Ying, Peng Gong

**Affiliations:** 1 Guangdong Key Laboratory for Biomedical Measurements and Ultrasound Imaging, National Regional Key Technology Engineering Laboratory for Medical Ultrasound, School of Biomedical Engineering, Shenzhen University Medical School, Shenzhen, 518060 China; 2 Department of General Surgery, Institute of Precision Diagnosis and Treatment of Digestive System Tumors and Guangdong Provincial Key Laboratory of Chinese Medicine Ingredients and Gut Microbiomics, Carson International Cancer Center, Shenzhen University General Hospital, Shenzhen University, Shenzhen, 518055 Guangdong China; 3 Woldia University, Woldia, Ethiopia; 4 Department of Physiology, School of Basic Medical Sciences, Center for Diabetes, Obesity and Metabolism, Shenzhen University Health Sciences Center, Shenzhen, 518060 Guangdong China

**Keywords:** Cancer, Genome-wide association studies, Mendelian randomization, Type 1 diabetes, Cancer, Oncology

## Abstract

Previous observational studies have suggested a potential link between Type 1 Diabetes (T1D) and site-specific cancer risk. However, the nature of this association remains uncertain due to confounding factors, reverse causation, and biases inherent in observational research. To address this gap, we conducted a two-sample Mendelian randomization (MR) study to assess the causal relationship between T1D and 22 site-specific cancers. Using summary statistics from large-scale genome-wide association studies of European ancestry, comprising data on T1D (N = 520,580) and the 22 site-specific cancers, we selected single nucleotide polymorphisms strongly associated with T1D as instruments for our analysis. Causal relationships were primarily evaluated through inverse-variance weighting-based analyses, supplemented by three additional methods: MR-Egger, weighted median, and mode-based estimate. Sensitivity analyses were performed, excluding genetic variants with potential pleiotropic effects. The finding demonstrated a causal association between T1D and increased risks of lung cancer (OR = 1.018, 95% CI 1.004–1.033, *p* = 0.011), colorectal cancer (OR = 1.022, 95% CI 1.003–1.041, *p* = 0.019), and prostate cancer (OR = 1.018, 95% CI 1.005–1.030, *p* = 0.006). Conversely, T1D was associated with decreased risks of breast cancer (OR = 0.989, 95% CI 0.981–0.998, *p* = 0.016), lymphoma (OR = 0.999, 95% CI 0.974–0.999, *p* = 0.003), malignant melanoma (OR = 0.999, 95% CI 0.989–0.999, *p* = 0.001), and non-melanoma skin cancer (OR = 0.999, 95% CI 0.899–0.999, *p* = 0.003). Our MR study provides an evidence of causal association between T1D and altered risks of various site-specific cancers. Further research is recommended to validate this finding in diverse populations to enhance the generalizability of findings across different ethnic groups.

## Introduction

Type 1 diabetes (T1D) and cancer are significant global health problems, each presenting its unique challenges to healthcare systems and individuals. T1D is a chronic disease characterized by autoimmune destruction of insulin-producing β cells in the pancreas^1^, affecting 8.4 million individuals worldwide in 2021, with an estimated to increase to 13.5–17.4 million by 2040^2^. Cancer remains the leading cause of morbidity and mortality, posing substantial societal, public health, and economic burdens^3^.

The co-occurrence of T1D and cancer has long intrigued researchers, prompting questions about potential shared etiological factors, biological mechanisms, and causal relationships. Epidemiological studies have shown varying associations between T1D and different cancer types, with some suggesting increased risk for certain cancers and others indicating a protective effect^4,5^. The underlying mechanisms are multifaceted, involving factors like chronic inflammation, immune dysregulation, shared genetic loci, and metabolic disturbances such as hyperglycemia and insulin-related pathways^6,7^.

Previous studies on the risk of cancer have primarily focused on type 2 diabetes (T2D), leaving a gap in understanding the relationship with T1D. Some observational studies, including those involving T1D, have yielded inconsistent findings, suggesting a site-specific risk of cancer^6^. For instance, a meta-analysis of 15 observational studies with 31,893 cancer patients found an increased risk of cancer among those with T1D^8^. However, a recent nationwide study in Finland, including 19,096 T1D patients, reported decreased cancer rates for urinary, respiratory, and intrathoracic organs^6^, consistent with findings from a long-term follow-up study in the UK involving 23,000 T1D patients, which showed an overall decreased risk of cancer^9^.

These studies have limitations due to their non-randomized design, making them susceptible to confounding factors, reverse causation, and inherent biases in observational research design^10^. Therefore, there is a compelling need for rigorous causal inference methods to comprehensively understand the nature of the association between T1D and cancer for effective prevention and management, leading to a significant reduction in disease burden.

Mendelian randomization (MR) emerges as a powerful tool in this context, to assess causal relationships using genetic variants as instrumental variables. It overcomes the methodological limitation of traditional observational study using genetic variants that are robustly associated with exposures like T1D as proxies. As genetic variants are randomly assorted at conception and fixed for life time, MR minimizes confounding and reverse causation, providing more reliable estimates of causal effects^11,12^. Therefore, the MR approach is conceptually similar to a randomized controlled trial (RCT) but being more feasible and cost-effective^13^.

Given the methodological limitations and inconsistent findings of previous observational studies, and lack of MR study between T1D and cancer, we conducted the two-sample MR analysis. This study aimed to investigate the causal association between T1D and 22 site-specific cancers using large-scale genome-wide association studies (GWAS) data.

## Methods

### Study design

**Fig. 1:**
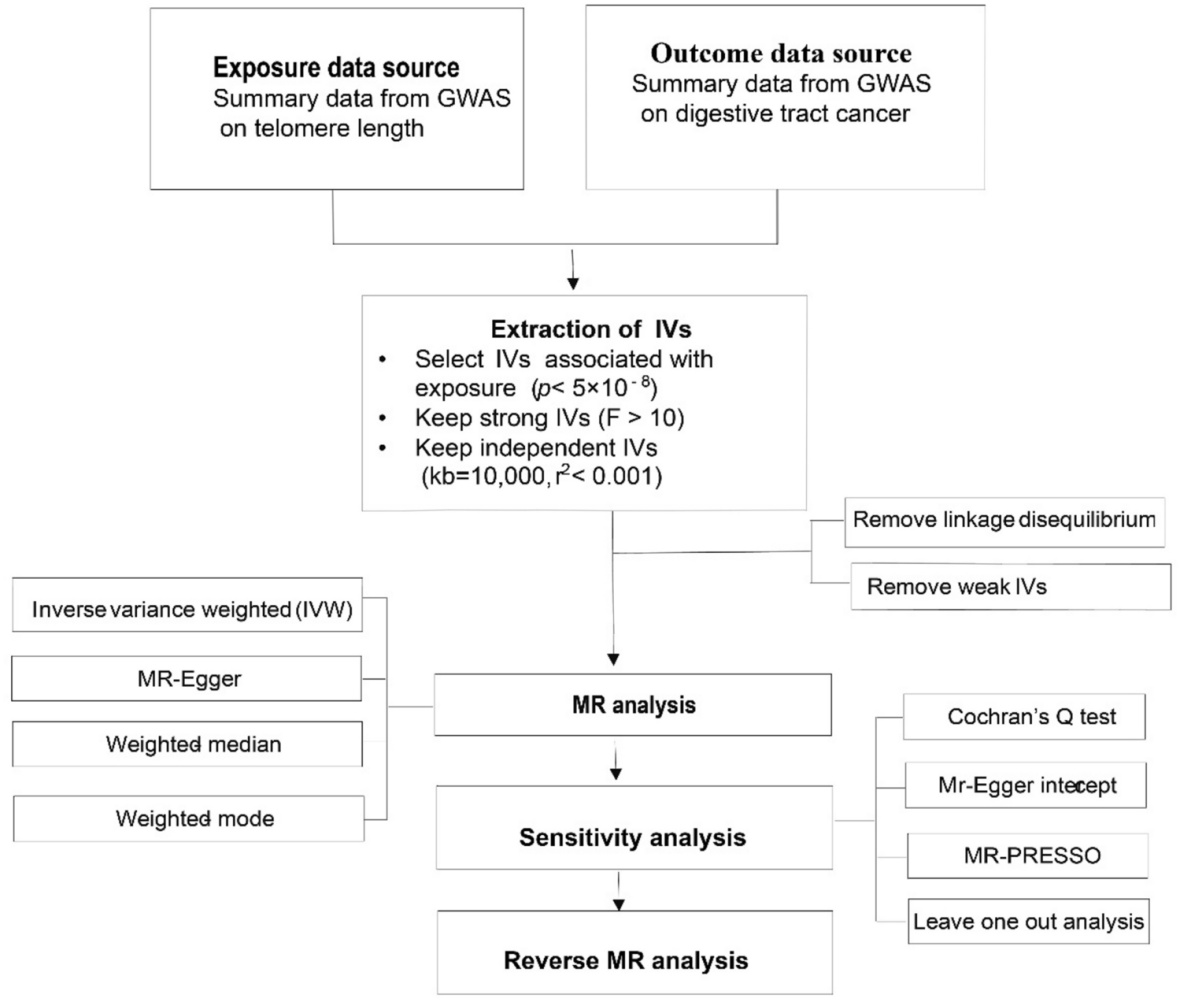
Mendelian randomization study flow chart.

The present study was conducted in accordance with Strengthening the Reporting of Observational Studies in Epidemiology—Mendelian Randomization (STROBE-MR) guideline^14^. We conducted a two-sample MR study using a single-nucleotide polymorphisms (SNPs) with T1D as instrument variable to assess the causal relationships with 22 site-specific cancer^12^.The study design is illustrated in Fig. 1 below.

**Fig. 2:**
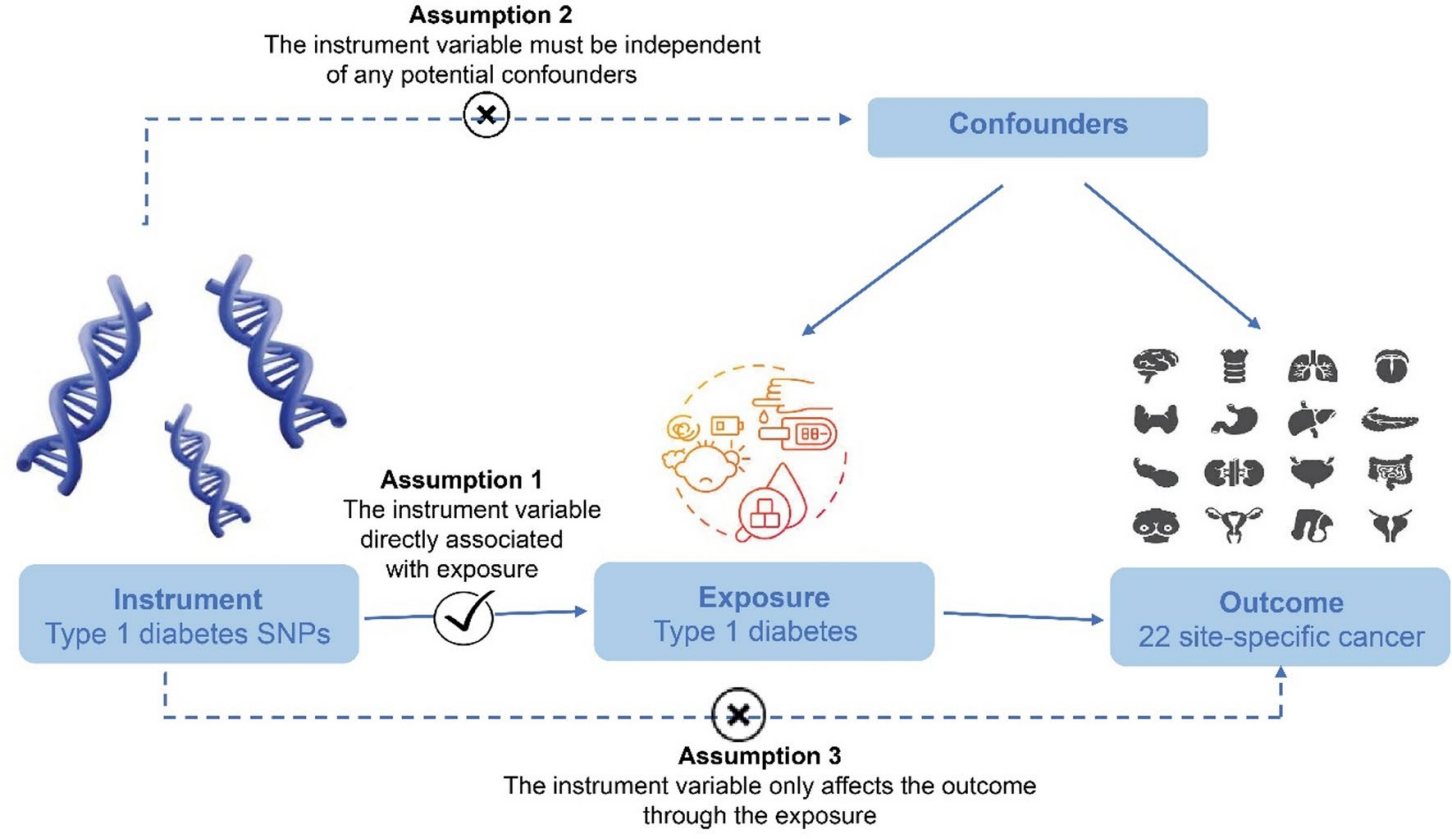
Basic assumptions of Mendelian randomization.

MR design was built based on three major assumptions^15^. The details of the assumption is found in Fig. 2.

### Data sources

We used publicly available large summary data from genome-wide association study (GWAS) database. All identified genetic variants of T1D used the data from the largest and latest GWAS study, in which 18,942 T1D patients and 501,638 control participants of European ancestry (Finland, Republic of Ireland, UK) from nine cohorts were enrolled. The total sample size was 520,580 participants with 60 million number of SNPs^16^. To our knowledge it is the largest and latest GWAS summary data available for T1D.

We have identified 22 site-specific cancer for which summary level data acquired from openGWAS database accessed on April 4th, 2024^17–19^. The 22 site-specific cancer includes: colorectal cancer, pancreatic cancer, gastric cancer, hepatic cancer, cholangiocarcinoma, esophageal cancer, breast cancer, ovarian cancer, cervical cancer, endometrial cancer, brain cancer, thyroid cancer, lung cancer, malignant neoplasm of kidney, bladder cancer, lymphoma, leukaemia, head and neck cancer, multiple myeloma, prostate cancer, malignant melanoma, and non-melanoma skin cancer. The total sample size of site-specific cancer data ranged from 32,072 to 491,974. The maximum sample overlapping rate between exposure data and outcome data is less than 1%.

We employed the following inclusion criteria for data selection: (1) When multiple datasets shared the same disease feature, we selected the dataset with the highest number of cases. If specific sample size for cases were unavailable in the current dataset, we chose the dataset with the largest overall sample size. (2) The GWAS cohort consisted entirely of individuals of European ancestry, and there was no overlap between the exposure and outcome cohorts. The details of included GWAS dataset is available on Table 1.

**Table 1:** Detailed information of GWAS datasets in the current study.

Traits	Population	Sample size	Number of SNPs	GWAS ID	Year
Exposure					
Type 1 diabetes	European	520,580	59,999,551	EBI-A-GCST90014023	2021
Outcome					
Colorectal cancer	European	32,072	38,356,021	EBI-A-GCST012879	2018
Pancreatic cancer	European	476,245	24,195,229	EBI-A-GCST90018893	2021
Gastric cancer	European	476,116	24,188,662	EBI-A-GCST90018849	2021
Hepatic cancer	European	475,638	24,194,938	EBI-A-GCST90018858	2021
Cholangiocarcinoma	European	476,091	24,196,592	EBI-A-GCST90018803	2021
Esophageal cancer	European	476,306	24,194,380	EBI-A-GCST90018841	2021
Breast cancer	European	83,691	10,680,257	IEU-A-1132	2017
Ovarian cancer	European	66,450	533,000	IEU-A-1120	2017
Cervical cancer	European	199,086	8,506,261	IEU-B-4876	2021
Endometrial cancer	European	121,885	9,470,555	EBI-A-GCST006464	2018
Thyroid cancer	European	491,974	24,198,226	EBI-A-GCST90018929	2021
Lung cancer	European	85,716	7,857,154	EBI-A-GCST004748	2017
Malignant neoplasm of kidney	European	463,010	9,851,867	UKB-B-1316	2018
Bladder cancer	European	462,933	9,851,867	UKB-B-8193	2018
Lymphoma	European	361,194	10,226,672	UKB-D-C_LYMPHOMA	2018
Non-melanoma skin cancer	European	462,933	9,851,867	UKB-B-12339	2018
Multiple myeloma	European	372,617	8,615,746	IEU-B-4957	2021
Prostate cancer	European	140,254	19,733,911	EBI-A-GCST006085	2018
Brain tumor	European	491,542	24,198,642	EBI-A-GCST90018800	2021
Malignant melanoma	European	462,933	9,851,867	UKB-B-12915	2018
Leukaemia	European	373,276	9,880,879	IEU-B-4914	2021
Head and neck cancer	European	373,122	9,655,080	IEU-B-4912	2021

GWAS ID: Genome-wide association studies identification; SNPs: Single nucleotide polymorphisms.

### Instrument variable selection

We have undertaken multiple steps to select the eligible genetic variants as IVs. First, single nucleotide polymorphisms (SNPs) influencing T1D were identified from openGWAS , based on a sample size of 18,942 cases and 501,638 controls. To satisfy the first MR assumption, we selected IVs associated with T1D at genome-wide statistical significance threshold (*p* < 5 × 10^−8^)^20^. Subsequently, we retained only the independent SNPs by excluding those in linkage disequilibrium (LD) with an r^2^ value of 0.001 and a window size of 10,000 kilobase (kb)^21^. This threshold is commonly used in MR studies to reduce potential bias from correlated genetic variants. In this case, the r^2^ < 0.001 threshold was selected to balance sufficient inclusion of variants while minimizing LD-related bias. Second, we computed F-statistics to assess the strength of the IVs on T1D ensuring they exceeded the minimal threshold value (F > 10) to reduce weak instrument bias^22^. For genetic variants not available in the outcome dataset, we used the LDproxy function in LD link (accessed on 20th April 2024) to select proxy SNPs (with an r^2^ > 0.8)^23^. Finally, SNPs absent in the outcome dataset were excluded from the analysis. Detailed information about the selected SNPs used as genetic instruments in this MR analysis is shown in Supplementary Table 1.

### Variant harmonization

Variant harmonization is important when combining summarized data on GWAS, as publicly available datasets may not consistently report strand information^20,24^. Therefore, we used variant harmonization process to exclude the SNPs with palindromic effect or have inconsistent alleles between the exposure and outcome dataset^24,25^. MR pleiotropy residual sum and outlier (MR-PRESSO) method were used to discard outlier SNPs^26^.

### Statistical power calculations

The statistical power of the MR analysis^27^ was calculated using the web based tool called mRND: Power calculations for Mendelian Randomization (https://shiny.cnsgenomics.com/mRND/ accessed on 19th April 2024). Power estimation considered factors like sample size, type-I error rate, proportion of cases, odds ratio (OR) of outcome variables per standard deviation of the exposure variable, and proportion of variance explained for the SNIP-exposure variable association (R^2^). The MR analysis had sufficient power (> 80%) to detect statistically significant effects, suggesting that the observed associations are unlikely to have occurred dueto random chance.

### Mendelian randomization analysis

Four MR methods were used to perform mendelian randomization analysis which includes IVW, MR Egger, weighted median, and weighted mode. Statistical analyses were conducted using packages such as “MendelianRandomization” (v.0.9.0), “TwoSampleMR” (v.0.5.10), “MRInstruments” (v.0.3.2), “MR-PRESSO” (v.1.0) in R (v.4.3.3). We performed a primary analysis using IVW two-sample MR to estimate the effect of T1D on 22 site-specific cancers susceptibility by regressing the SNPs in the exposure data against SNPs in the outcome data. The IVW method was performed using a multiplicative random-effects model due to the presence of multiple variants from different gene region. The IVW method with multiplicative random-effects is the most efficient analysis that provides valid estimates based on the assumption of balanced pleiotropy^20^. Additionally, we employed MR Egger, weighted median, and weighted mode methods to complement the IVW result.

The causal relationship was determined based on the following criteria^14^: (1) The statistical significance value of the MR analysis mainly the IVW should be (*p* < 0.05)^28^ (2) Consistency of the directions of the b values for each of the five MR methods. A value of less than 0 for all five b values suggests a negative causal relationship, while a value greater than 0 indicates a positive causal relationship. (3) An OR < 1 indicates that the exposure is a potential protective factor for the outcome, whereas an OR > 1 suggests that the exposure is a risk factor for the outcome. If a causal relationship is detected using IVW method based on these criteria, then sensitivity analyses were performed to assess the robustness of the finding to the assumption of balanced pleiotropy^20^.

### Sensitivity analysis

A sensitivity analysis were conducted by excluding genetic variants associated with potential confounders. Specifically, MR-Egger, weighted median, and MR-PRESSO were utilized to investigate potential pleiotropic effects that could bias the MR estimates^29^.

The second MR assumption which requires no associations among IVs and confounding factors was evaluated by exploring IVs associated with common cancer risk factors, such as BMI, T2D, smoking, alcohol consumption and physical activity, using GWAS summary data^6^ and the Ldlink tool^30^, a web-based collection of bioinformatic modules.

The third MR assumption, which states that IVs should be associated with the outcome solely through the exposure^26^. We examined horizontal pleiotropy using the MR-Egger intercept, (*p*-value < 0.05 indicating pleiotropy)^31^ and, MR-PRESSO to detect and adjust for outliers^26^, alongside a visual inspection via funnel plot assessment^32^. Additionally, we conducted weighted median analysis, assuming the validity of the majority of IVs when less than 50% of the total weight comes from IVs with horizontal pleiotropy^33^.

Furthermore, heterogeneity testing was performed using Cochran’s Q test^32^ and funnel plot inspection, where a symmetric plots suggesting low heterogeneity. Leave-one-out analyses were conducted to explore the effect of individual IVs on the overall MR estimate. Scatter plots were used to visualize summary data, aiding in the interpretation of the standard IVW estimate, MR-Egger regression, weighted median, weighted mode and simple mode. Finally, the MR Steiger directionality test^34^ was performed to detect potential reverse causation. These methods collectively provide a comprehensive assessment of the MR assumptions and the robustness of our findings.

## Result

### Genetic instruments

A total of 89 SNPs were used in the MR analysis to genetically predict the causal relationship between T1D and 22 site-specific cancers. We used Ldlink, a web-based tool^30^, to investigate whether SNPs in the MR analysis were associated with confounding traits linked to cancer, such as BMI, T2D, smoking, alcohol consumption, and physical activity. The analysis revealed that no SNPs were connected to these confounding traits, which might influence cancer risk independent of T1D. The F-statistics for the included IVs ranged from 29.866 to 8073.918, indicating no weak instrument bias in this MR analysis^22^.

SNP details on the association of T1D and 22 site-specific cancers is shown in Supplementary Tables 2–23.

### MR analysis

**Fig. 3:**
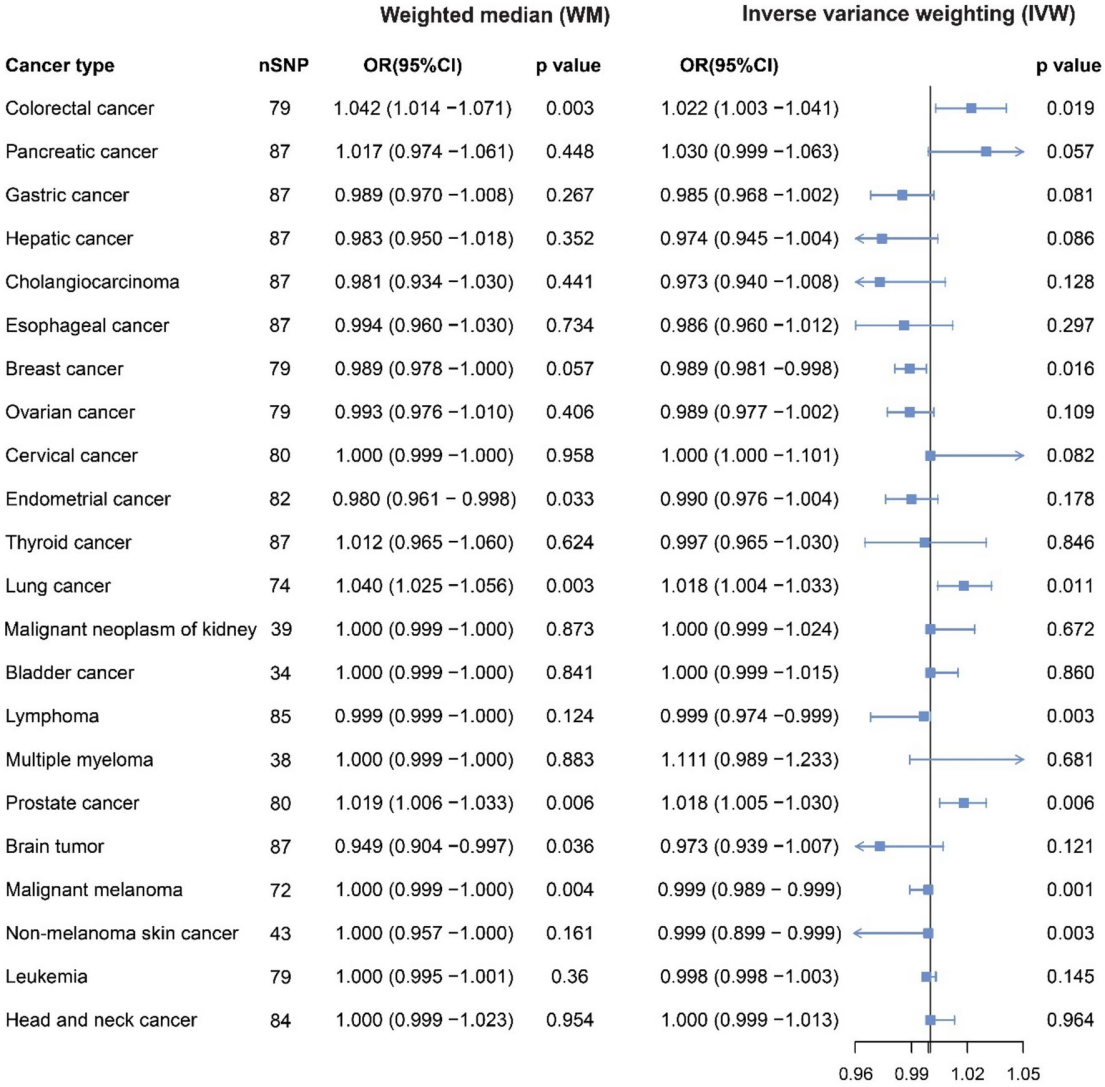
Forest plot of MR study investigating the causal association between T1D and 22 site-specific cancers.

The IVW results of the MR analysis showed an evidence of causal associations of T1D with an increased risks of lung cancer (OR = 1.018, 95% CI 1.004–1.033, *p* = 0.011), colorectal cancer (OR = 1.022, 95% CI 1.003–1.041, *p* = 0.019), prostate cancer (OR = 1.018, 95% CI 1.005–1.030, *p* = 0.006) and decreased risks of breast cancer (OR = 0.989, 95% CI 0.981–0.998, *p* = 0.016), lymphoma (OR = 0.999, 95% CI 0.974–0.999, *p* = 0.003), malignant melanoma (OR = 0.999, 95% CI0.989–0.999, *p* = 0.001), and non-melanoma skin cancer (OR = 0.999, 95% CI0.899–0.999, *p* = 0.003). (Fig. 3) The other three MR methods (weighted median, MR Egger and MR mode) also found the same result regarding the causal association. The b-values were consistently calculated in the same direction across all four MR methods, with IVW being the most critical method.

Moreover, we found no casual association between TID and pancreatic cancer (*p* = 0.057), gastric cancer (*p* = 0.081), hepatic cancer (*p* = 0.086), cholangiocarcinoma (*p* = 0.128), esophageal cancer (*p* = 0.297), ovarian cancer (*p* = 0.109), cervical cancer (*p* = 0.082), thyroid cancer (*p* = 0.846), malignant neoplasm of kidney (*p* = 0.672), bladder cancer (*p* = 0.860), multiple myeloma (*p* = 0.681), brain tumor (*p* = 0.121), leukemia (*p* = 0.145), and head and neck cancer (*p* = 0.964). (Fig. 3).

The MR Steiger’s directionality test revealed no indications of reverse causality between T1D and all types of cancer, as shown in the Supplementary Table 24. Consequently, the inferred direction of the causal association between T1D and various cancer types was deemed reliable. Furthermore, the MR analyses conducted in this study displayed no evidence of horizontal pleiotropy, as indicated in Table 2.

**Table 2:** Cochran’s Q test and MR-Egger intercept of MR analyses for T1D and risk of cancer.

Exposure	Outcome	Heterogeneity			Plieotropy		
		No. of SNPs	Cochran’s Q statistic^†^	*p*-value	MR-egger intercept^‡^	SE	*p*-value
Type 1 diabetes	Colorectal cancer	79	53.331	0.985	− 4.21E-03	3.27E-03	0.202
	Pancreatic cancer	87	93.136	0.281	− 1.63E-03	6.30E-03	0.797
	Gastric cancer	87	111.126	0.036	1.05E-03	3.41E-03	0.758
	Hepatic cancer	87	101.832	0.117	2.89E-03	5.95E-03	0.628
	Cholangiocarcinoma	87	87.633	0.431	8.99E-05	7.12E-03	0.990
	Esophageal cancer	87	67.708	0.927	− 5.37E-03	5.29E-03	0.313
	Breast cancer	79	85.326	0.267	1.49E-03	1.86E-03	0.425
	Ovarian cancer	79	86.165	0.247	− 1.12E-03	2.45E-03	0.650
	Cervical cancer	80	58.174	0.962	− 1.28E-05	3.17E-05	0.686
	Endometrial cancer	82	97.498	0.102	− 2.81E-05	2.89E-03	0.992
	Thyroid cancer	87	77.349	0.736	5.72E-03	6.59E-03	0.388
	Lung cancer	74	149.294	< 0.001	− 4.04E-03	2.90E-03	0.167
	Malignant neoplasm of kidney	39	52.172	0.040	− 2.21E-05	2.80E-05	0.436
	Bladder cancer	34	38.204	0.245	2.06E-06	2.62E-05	0.938
	Lymphoma	85	120.827	0.005	− 1.62E-05	3.62E-05	0.655
	Multiple myeloma	38	47.231	0.121	4.52E-05	3.49E-05	0.204
	Prostate cancer	80	132.316	< 0.001	− 1.49E-03	2.03E-03	0.466
	Brain tumor	87	70.865	0.881	1.27E-02	7.04E-03	0.074
	Malignant melanoma	72	93.212	0.040	− 6.28E-05	3.85E-05	0.107
	Non-melanoma skin cancer	43	53.887	0.103	− 4.00E-05	3.11E-05	0.205
	Leukeamia	79	75.851	0.548	− 8.12E-06	2.94E-05	0.783
	Head and neck cancer	84	74.630	0.733	− 3.63E-06	2.35E-05	0.878

SNPs: single nucleotide polymorphisms; SE: standard error.

^†^The Cochran’s Q test is a statistical test for heterogeneity.

^‡^The intercept term from the MR-Egger regression method is a statistical test of horizontal pleiotropy.

### Sensitivity analysis

Sensitivity analysis, including heterogeneity testing, horizontal pleiotropy testing, and leave-one-out analysis, was conducted to evaluate the robustness of the MR analysis results. The sensitivity tests revealed results consistent with the primary MR analysis. According to the MR-Egger interecept (*P*_interecept_ > 0.05), the sensitivity analysis provided no evidence of horizontal pleiotropy across all analyses (Table 2).

**Fig. 4:**
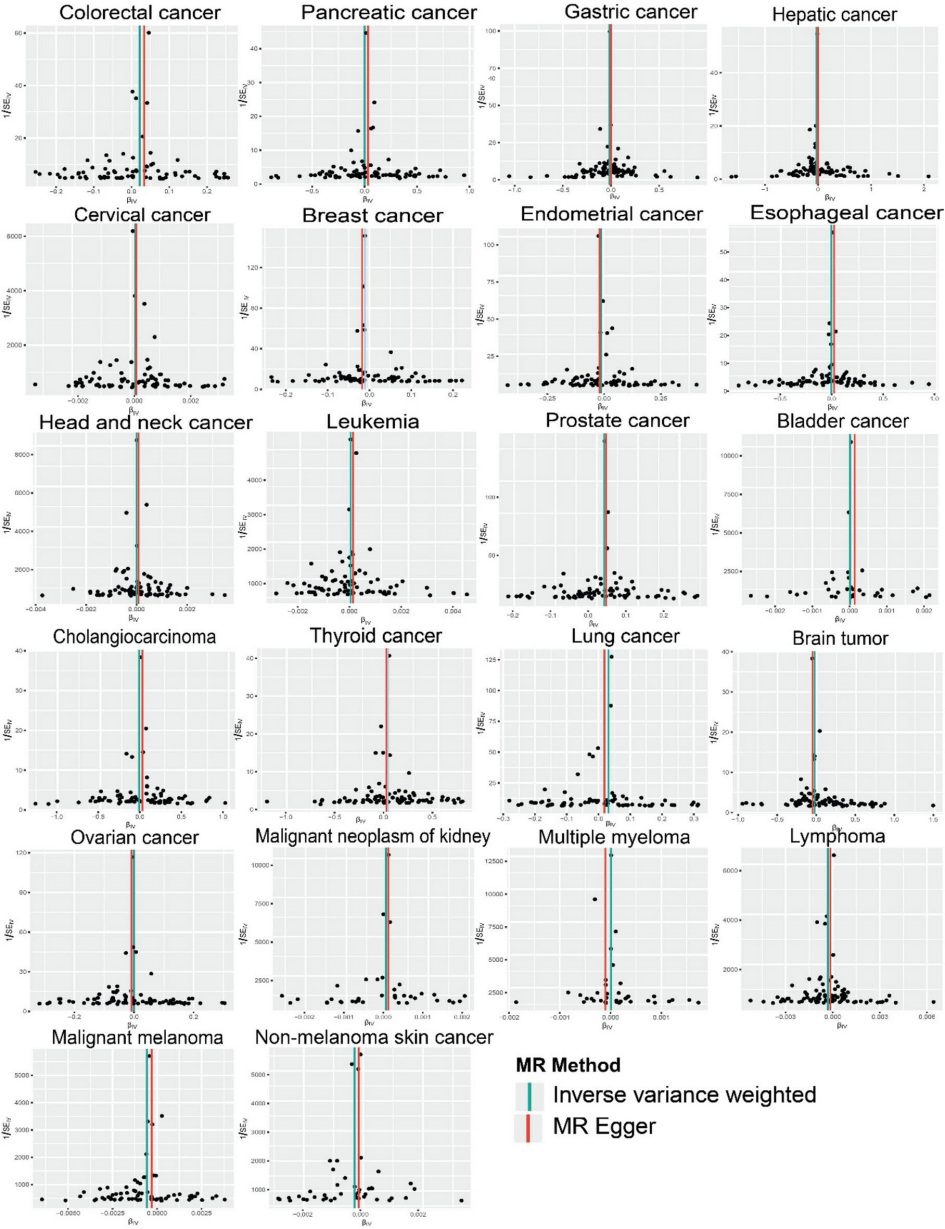
Funnel plot of MR study investigating the effect of T1D on 22 site-specific cancers. The funnel plot provides a visual representation of the precision of estimated effects (β_*IV*_) from IVW and MR Egger method of analyses assessing the impact of T1D on 22 site-specific cancers. The x-axis represents the effect size (β_*IV*_), while the y-axis represents the inverse of the standard error (1/SE_*IV*_), acting as a measure of precision. A symmetrical distribution of points around the estimated effect, resembling a funnel shape is shown. MR-Egger’s regression test or visual inspection of funnel plot indicated minimal heterogeneity in the study results.

While no heterogeneity was observed among the instrument variables associated with 16 types of cancer, significant heterogeneity (*p* < 0.05) was detected in the instrument variables used to establish causal associations between T1D and gastric cancer (*p* = 0.036), lung cancer (*p* < 0.001), malignant neoplasm of the kidney (*p* = 0.040), lymphoma, prostate cancer (*p* < 0.001), and malignant melanoma (*p* = 0.040). The causal effect of exposure on the outcome was estimated using the random-effects IVW method for casual associations with significant heterogeneity. The results of random-effects IVW indicated no heterogeneity for gastric cancer (*p* = 0.080) and malignant neoplasm of the kidney (*p* = 0.670). In the absence of heterogeneity, the fixed-effect model was employed. In addition, symmetrical funnel plots suggested low heterogeneity among the genetic variants in all analyses (Fig. 4).

Furthermore, leave-one-out analyses reaffirmed the reliability of the causal effects of individual instrument variables on the overall MR estimate, as illustrated in Supplementary Figs. 1–22. Outlier SNPs were identified and corrected using MR-PRESSO. In addition, T1D-associated SNPs showed no associations with potential confounders.

**Fig. 5:**
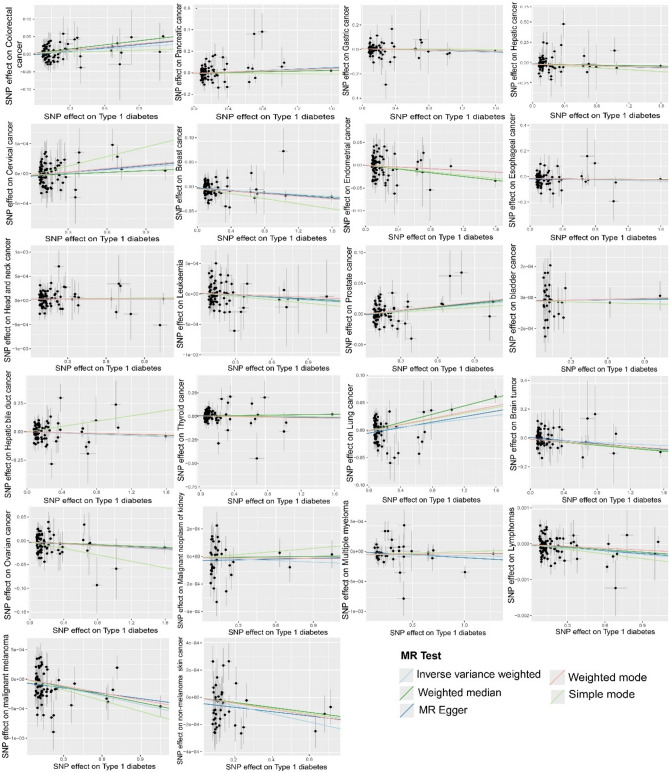
Scatter plot demonstrates a significant linear association between T1D and 22 site-specific cancers, while no discernible heterogeneity of SNPs was observed. The black dots correspond to the SNPs employed in the MR analysis. The lines depict the linear fitting trends derived from various analysis methods employed in the MR analysis. Each line represents different methods of MR analysis. The light blue line represents the fitting trend obtained through the IVW method, the dark blue line represents the fitting trend obtained through the MR-Egger method, the green line represents the fitting trend obtained through the weighted median method, the light red line represents the fitting trend obtained through the weighted mode method, and the light yellow green line represents the fitting trend obtained through the simple mode method. The x-axis represents the genetic association with T1D and the y-axis represents the genetic association with 22 site-specific cancers.

The scatter plot illustrating the MR estimate of T1D effect on different types of cancer exhibited a distinct linear trend. (Fig. 5).

## Discussion

Extensive research has recently focused on the causal association between T2D and cancer, consistently demonstrating T2D’s significant role in cancer development^35–37^. However, no previous study has investigated the causal associations of T1D and cancer. Therefore, our MR study aimed to assess this relationship, thereby expanding knowledge in the field of diabetes and cancer research. The result of this study found that T1D was associated with an increased risks of lung, colorectal, and prostate cancers as well as decreased risks of breast cancer, lymphoma, malignant melanoma, and non-melanoma skin cancer.

Previous observational studies^4,5,8,9,38,39^ investigating cancer risk among T1D patients have yielded inconsistent results regarding both overall and site-specific cancer risks. These inconsistencies might arise from factors such as misclassifications of diabetes, variation in followup periods, sample size differences, or other variations in study design. Therefore, our MR study resolves inconsistencies through standardized methodology of MR and the use of genetic variants as proxies for exposures, which minimize the impact of design, population, and methodology variations that can lead to heterogeneous results in cohort studies. This approach is particularly useful for studying long-term effects and chronic conditions, where traditional cohort studies may face challenges related to follow-up periods and attrition^11^.

Our study findings align with a meta-analysis involving 1,915,179 participants^8^ and another cohort study from the Australian National Diabetes Registry with 80,676 participants^7^ showing a consistent positive association between T1D and lung cancer. Possible mechanisms includes increased levels of insulin-like growth factor-1 (IGF-1) due to compensatory hyperinsulinemia, which may contribute to tumor development and progression^40^. Additionally, hyperglycemia can lead to structural damage in the lung such as emphysema, an independent risk factor for lung cancer^41,42^. However, another cohort study showed decreased risk of lung cancer among T1D, which is explained by reduction of smoking in older onset diabetes patients^9^.

Our study also identified positive associations of T1D with colorectal and prostate cancer demonstrating high statistical power of 98% and 100%, respectively. The finding was supported by studies from Australian National Diabetes Registry^7^ and Taiwan^38^ highlighting the association between TID and colorectal cancer. Insulin therapy-induced exogenous hyperinsulinemia may contribute to the proliferation of colon and rectum epithelial cells^43^. This is due to direct activation of insulin receptor and inhibition of IGF binding protein. Elevated C-peptide levels, a marker of insulin secretion, and an increased colorectal cancer risk^44,45^. Additionally, long-term insulin therapy in T1D, due to autoimmune destruction of pancreatic β-cells, may lead to elevated insulin concentrations in the circulation and potential mutagenic effects of insulin analogs, increasing the risk of certain cancers^46^. In line with a cohort study^38^, our research also indicates a positive association between T1D and prostate cancer. The increased risk observed in our study could be attributed to factors such as chronic inflammation, hormonal imbalances, and hyperglycemia. In addition, the higher statistical power (100%) of our study indicates credibility and robustness to its findings.

Our finding of an inverse association between T1D and breast cancer is in line with most observational studies but not all. A large study of five nationwide diabetes registries^5^ and a meta-analysis of 15 observational studies^8^ reported a decreased risk of breast cancer in T1D patients. However, some previous observational studies conducted in Taiwan^38^, Finland^6^, and UK^9^, did not confirm our result. The observed discrepancy in the association between T1D and breast cancer across various studies might be due to variations in study design, sample size, criteria for defining T1D, breast cancer diagnosis, and the duration of follow-up, leading to varying interpretations of the relationship. Mendelian randomization studies, which effectively control confounding variables are more likely to provide reliable estimates than observational studies with limited control over confounders. Furthermore, the statistical power for the inverse association between T1D and breast cancer was high, at 92%, indicating a greater ability to detect a genuine association between the two variables.

The reduced risk of malignant melanoma among individuals with T1D aligns with findings from other large T1D cohorts^5^. Additionally, supporting evidence also comes from another cohort study^7^ and a Mendelian randomization study^35^ on T2D and cancer risk. Moreover, our study’s finding of decreased non-melanoma skin cancer is consistent with a large cohort study among 23,000 T1D patients^9^. The mechanism underlying the reduced risk of malignant melanoma in T1D individuals can be multifactorial. One potential explanation is the altered immune response associated with T1D, which can impact immune surveillance mechanisms against cancer development, including malignant melanoma. Autoimmune processes in T1D may modulate these mechanisms, contributing to a decreased incidence of certain cancers. Furthermore, the similarity in the reduction of malignant melanoma risk in both diabetes types might be attributed to diabetes management, which often involves monitoring blood glucose levels and treatments that can influence cellular pathways linked to cancer development. These treatments might indirectly affect malignant melanoma and non-melanoma risk by altering metabolic and signaling pathways involved in cancer progression. However, the underlying mechanisms for these protective effects of T1D warrant further investigation.

The identification of specific cancer types associated with T1D has important implications for clinical practice. Given T1D is a lifelong disease, healthcare providers should be aware of the potential increased risk of lung, colorectal, and prostate cancers among T1D patients, which may necessitate targeted screening and early detection strategies.

### Strength and limitations

This study exhibits several notable strengths. Firstly, it is the first MR study that comprehensively evaluated the causal association of T1D with 22 site-specific cancers, utilizing the most recent and extensive GWAS data available. Secondly, by employing MR analysis techniques, potential biases such as confounding and reverse causation, common in observational studies, were minimized. Thirdly, rigorous sensitivity analyses were conducted to validate the consistency of the causal effects. Additionally, stringent quality control measures including Cochrane’s Q test, MR-Egger intercept test, and the MR-PRESSO method were employed to systematically assess pleiotropy or violations of MR assumptions, with no detected pleiotropy enhancing the reliability of the MR analysis. Moreover, consistent results were observed across different datasets, ensuring the robustness of the findings.

However, there are still several limitations should be acknowledged. First, the GWAS data utilized in this study predominantly comprised participants of European ancestry, limiting the generalizability of our findings to other populations. Additionally, although the two-sample MR approach was employed, the data used came from a single platform. While the sample datasets were not entirely overlapping, the potential for bias arising from using data sourced from the same platform warrant attention. Second, while MR analysis is a powerful technique for identifying causal relationships, it has inherent methodological limitations. Specifically, MR relies on three key assumptions that are difficult to fully validate in practice. Any violations of these assumptions could lead to biased estimates. Although sensitivity analyses were conducted to assess the potential for pleiotropy, residual pleiotropic effects cannot be entirely ruled out. Furthermore, MR analysis operates at the population level, meaning the findings may not fully reflect individual-level causal relationships, which should be carefully considered when translating these results to clinical practice. Finally, due to the lack of detailed information on variables such as age, sex, and T1D severity in summary-level data, subgroup analysis was not feasible.

Despite employing robust methods to address heterogeneity and pleiotropy, potential biases from unknown pleiotropic effects cannot be fully ruled out, urging careful interpretation of the results.

In conclusion, while this study provides valuable insights into the causal association between T1D and cancer risk, the findings must be interpreted with caution, and further research is recommended to expand GWAS studies to include more diverse populations to enhance the generalizability of findings across different ethnic groups. Additionally, investigating the impact of specific demographic and clinical factors, such as age, sex, and T1D severity, on the observed causal associations can provide valuable insights into personalized risk assessment and targeted interventions.

## Conclusion

Our two-sample MR study provides evidence of a causal association between T1D and increased risk of lung, colorectal, and prostate cancers as well as decreased risks of breast cancer, lymphoma, malignant melanoma, and non-melanoma skin cancer. These findings underscore the importance of personalized cancer screening and prevention strategies tailored to individuals with T1D. Further research is recommended to validate this finding in diverse populations to enhance the generalizability of findings across different genetic backgrounds.

## Supplementary Information

Supplementary Information 1.

Supplementary Information 2.

Supplementary Information 3.

## Data Availability

The dataset(s) supporting the conclusions of this article were obtained from IEU OpenGWAS (https://gwas.mrcieu.ac.uk/).
