# Supplementary material for: A two-sample Mendelian randomization study of type 1 diabetes and the risk of 22 site-specific cancers
Source: Sci Rep. 2025 Apr 3;15:11371. doi: 10.1038/s41598-025-89288-3 (PMC11965372; doi:10.1038/s41598-025-89288-3)
Supplement: Supplementary file 1 — Supplementary Information 1. [file 41598_2025_89288_MOESM1_ESM.docx]

**Supplementary Table 1**

Summary information of genetic instruments/SNPs associated with type 1 diabetes.

| **SNPs** | **NEA** | **EA** | **EAF** | **β** | **SE** | **P-value** | **R^2^** | **F** |
| --- | --- | --- | --- | --- | --- | --- | --- | --- |
| rs10751776 | A | C | 0.51 | 0.078 | 0.014 | 2.67E-08 | 5.94E-05 | 30.935 |
| rs855330 | T | C | 0.26 | 0.111 | 0.017 | 4.89E-11 | 8.30E-05 | 43.219 |
| rs12128789 | T | C | 0.13 | 0.127 | 0.022 | 3.73E-09 | 6.68E-05 | 34.762 |
| rs10801128 | A | G | 0.72 | 0.096 | 0.016 | 8.98E-10 | 7.21E-05 | 37.536 |
| rs17623914 | T | C | 0.10 | -0.135 | 0.023 | 7.97E-09 | 6.39E-05 | 33.282 |
| rs7511678 | G | A | 0.23 | 0.094 | 0.017 | 3.09E-08 | 5.89E-05 | 30.650 |
| rs12742756 | A | G | 0.43 | -0.083 | 0.015 | 3.54E-08 | 5.84E-05 | 30.384 |
| rs6679677 | C | A | 0.11 | 0.642 | 0.021 | 1.00E-200 | 1.80E-03 | 939.332 |
| rs3024493 | C | A | 0.15 | -0.164 | 0.020 | 7.26E-17 | 1.34E-04 | 69.597 |
| rs574384 | C | A | 0.90 | -0.134 | 0.024 | 2.20E-08 | 6.01E-05 | 31.311 |
| rs2493411 | T | C | 0.13 | 0.127 | 0.022 | 1.28E-08 | 6.22E-05 | 32.364 |
| rs3087243 | G | A | 0.42 | -0.199 | 0.014 | 1.16E-44 | 3.78E-04 | 196.595 |
| rs4490209 | C | G | 0.36 | -0.085 | 0.015 | 4.55E-08 | 5.74E-05 | 29.901 |
| rs1881146 | A | T | 0.31 | -0.095 | 0.017 | 4.57E-08 | 5.74E-05 | 29.892 |
| rs12464462 | A | G | 0.41 | -0.088 | 0.014 | 8.61E-10 | 7.23E-05 | 37.615 |
| rs2111485 | A | G | 0.60 | 0.128 | 0.014 | 1.05E-18 | 1.50E-04 | 77.960 |
| rs6434435 | G | A | 0.16 | -0.123 | 0.019 | 1.23E-10 | 7.96E-05 | 41.421 |
| rs55893453 | A | G | 0.20 | 0.095 | 0.017 | 4.63E-08 | 5.74E-05 | 29.866 |
| rs28648882 | G | A | 0.22 | 0.099 | 0.018 | 3.03E-08 | 5.89E-05 | 30.686 |
| rs7668577 | A | C | 0.31 | 0.094 | 0.015 | 7.26E-10 | 7.29E-05 | 37.947 |
| rs2611211 | C | T | 0.82 | -0.144 | 0.019 | 1.39E-14 | 1.14E-04 | 59.248 |
| rs12644686 | C | G | 0.19 | -0.108 | 0.019 | 2.44E-08 | 5.97E-05 | 31.104 |
| rs13147049 | A | G | 0.64 | -0.110 | 0.015 | 8.92E-14 | 1.07E-04 | 55.593 |
| rs2303137 | A | T | 0.44 | -0.082 | 0.014 | 6.17E-09 | 6.49E-05 | 33.779 |
| rs114378220 | C | T | 0.07 | 0.178 | 0.030 | 5.11E-09 | 6.56E-05 | 34.147 |
| rs2188962 | C | T | 0.40 | 0.079 | 0.014 | 1.73E-08 | 6.10E-05 | 31.776 |
| rs9260802 | A | G | 0.04 | -0.356 | 0.042 | 1.35E-17 | 1.40E-04 | 72.925 |
| rs7752257 | T | G | 0.74 | -0.680 | 0.017 | 1.00E-200 | 3.21E-03 | 1677.550 |
| rs6908626 | G | T | 0.17 | 0.203 | 0.019 | 6.14E-28 | 2.31E-04 | 120.055 |
| rs28752526 | A | G | 0.36 | 0.630 | 0.016 | 1.00E-200 | 3.01E-03 | 1569.786 |
| rs4548024 | T | C | 0.23 | -0.096 | 0.017 | 9.95E-09 | 6.31E-05 | 32.853 |
| rs1050979 | A | G | 0.52 | 0.106 | 0.014 | 5.65E-14 | 1.09E-04 | 56.493 |
| rs1611236 | G | A | 0.30 | -0.260 | 0.017 | 1.83E-50 | 4.29E-04 | 223.172 |
| rs3135348 | A | G | 0.56 | 1.048 | 0.017 | 1.00E-200 | 6.89E-03 | 3610.106 |
| rs9468618 | C | T | 0.08 | -0.272 | 0.026 | 5.10E-25 | 2.05E-04 | 106.730 |
| rs2429557 | T | A | 0.01 | 1.021 | 0.058 | 4.98E-69 | 5.92E-04 | 308.356 |
| rs9385401 | C | T | 0.45 | 0.120 | 0.015 | 4.59E-16 | 1.27E-04 | 65.961 |
| rs238873 | A | G | 0.01 | 0.793 | 0.053 | 4.30E-50 | 4.25E-04 | 221.481 |
| rs73432769 | C | T | 0.02 | 0.335 | 0.046 | 2.34E-13 | 1.03E-04 | 53.693 |
| rs112733823 | C | T | 0.14 | 0.383 | 0.020 | 7.42E-81 | 6.96E-04 | 362.648 |
| rs1794269 | C | T | 0.43 | 1.594 | 0.018 | 1.00E-200 | 1.53E-02 | 8073.918 |
| rs6908236 | A | C | 0.51 | 0.166 | 0.014 | 6.31E-32 | 2.66E-04 | 138.290 |
| rs7795896 | C | T | 0.69 | -0.135 | 0.016 | 1.58E-16 | 1.31E-04 | 68.065 |
| rs17323934 | C | G | 0.22 | -0.130 | 0.017 | 1.26E-14 | 1.14E-04 | 59.440 |
| rs10275896 | T | C | 0.23 | -0.121 | 0.017 | 2.60E-13 | 1.03E-04 | 53.488 |
| rs7776597 | A | G | 0.96 | 0.244 | 0.036 | 1.82E-11 | 8.67E-05 | 45.156 |
| rs10224046 | T | G | 0.32 | 0.086 | 0.015 | 2.71E-08 | 5.94E-05 | 30.904 |
| rs13259300 | A | C | 0.60 | -0.092 | 0.015 | 3.28E-10 | 7.59E-05 | 39.498 |
| rs1947178 | A | G | 0.79 | -0.103 | 0.017 | 1.67E-09 | 6.98E-05 | 36.325 |
| rs3802214 | T | C | 0.80 | -0.107 | 0.019 | 2.96E-08 | 5.90E-05 | 30.735 |
| rs1574285 | G | T | 0.59 | -0.127 | 0.014 | 4.27E-19 | 1.53E-04 | 79.746 |
| rs78325861 | C | G | 0.04 | -0.282 | 0.042 | 2.31E-11 | 8.58E-05 | 44.692 |
| rs12257077 | C | T | 0.03 | 0.231 | 0.037 | 3.91E-10 | 7.52E-05 | 39.156 |
| rs7068821 | G | T | 0.25 | -0.165 | 0.016 | 5.07E-24 | 1.96E-04 | 102.182 |
| rs61839660 | C | T | 0.09 | -0.357 | 0.026 | 5.25E-43 | 3.63E-04 | 188.999 |
| rs41295159 | C | G | 0.01 | -0.700 | 0.090 | 9.11E-15 | 1.15E-04 | 60.079 |
| rs722988 | T | C | 0.35 | 0.083 | 0.014 | 9.78E-09 | 6.32E-05 | 32.887 |
| rs7936434 | G | C | 0.46 | 0.077 | 0.014 | 3.58E-08 | 5.83E-05 | 30.367 |
| rs7130222 | T | G | 0.31 | -0.092 | 0.016 | 1.30E-08 | 6.21E-05 | 32.324 |
| rs663743 | G | A | 0.35 | -0.100 | 0.015 | 3.50E-11 | 8.43E-05 | 43.872 |
| rs607703 | C | T | 0.48 | 0.092 | 0.014 | 1.17E-10 | 7.98E-05 | 41.520 |
| rs689 | A | T | 0.73 | 0.712 | 0.019 | 1.00E-200 | 2.83E-03 | 1476.890 |
| rs1701704 | T | G | 0.34 | 0.244 | 0.015 | 4.52E-63 | 5.40E-04 | 281.025 |
| rs7301381 | T | C | 0.46 | -0.094 | 0.014 | 5.25E-11 | 8.28E-05 | 43.085 |
| rs10844597 | G | A | 0.51 | -0.090 | 0.014 | 1.20E-10 | 7.96E-05 | 41.461 |
| rs3184504 | T | C | 0.53 | -0.231 | 0.014 | 1.08E-60 | 5.19E-04 | 270.096 |
| rs238265 | T | G | 0.70 | -0.091 | 0.015 | 2.08E-09 | 6.90E-05 | 35.903 |
| rs9517712 | T | C | 0.74 | -0.102 | 0.016 | 1.06E-10 | 8.01E-05 | 41.701 |
| rs17106304 | C | G | 0.66 | 0.115 | 0.015 | 6.83E-15 | 1.16E-04 | 60.648 |
| rs1350275 | T | G | 0.70 | -0.094 | 0.015 | 8.86E-10 | 7.21E-05 | 37.561 |
| rs56994090 | T | C | 0.43 | -0.134 | 0.015 | 3.60E-20 | 1.63E-04 | 84.627 |
| rs34593439 | G | A | 0.11 | -0.218 | 0.024 | 1.54E-19 | 1.57E-04 | 81.748 |
| rs12927355 | C | T | 0.32 | -0.204 | 0.015 | 4.41E-41 | 3.46E-04 | 180.198 |
| rs231972 | A | C | 0.12 | 0.171 | 0.021 | 5.12E-16 | 1.26E-04 | 65.750 |
| rs55993634 | C | G | 0.08 | 0.219 | 0.024 | 2.29E-19 | 1.56E-04 | 80.970 |
| rs8046043 | G | C | 0.39 | -0.085 | 0.015 | 2.49E-08 | 5.97E-05 | 31.070 |
| rs57209021 | C | T | 0.23 | 0.101 | 0.018 | 3.73E-08 | 5.82E-05 | 30.286 |
| rs61759532 | C | T | 0.24 | 0.118 | 0.019 | 1.91E-10 | 7.79E-05 | 40.563 |
| rs35327136 | C | A | 0.16 | -0.119 | 0.019 | 3.37E-10 | 7.58E-05 | 39.448 |
| rs1808094 | T | C | 0.52 | -0.114 | 0.015 | 2.40E-14 | 1.12E-04 | 58.180 |
| rs7237497 | T | C | 0.84 | -0.220 | 0.019 | 2.71E-32 | 2.69E-04 | 139.966 |
| rs34536443 | G | C | 0.04 | -0.385 | 0.039 | 1.47E-23 | 1.92E-04 | 100.073 |
| rs601338 | G | A | 0.48 | 0.127 | 0.014 | 1.20E-18 | 1.49E-04 | 77.695 |
| rs113374757 | C | T | 0.16 | -0.171 | 0.021 | 1.63E-16 | 1.31E-04 | 68.009 |
| rs202535 | C | A | 0.83 | -0.141 | 0.018 | 1.79E-14 | 1.13E-04 | 58.748 |
| rs11203203 | G | A | 0.35 | 0.144 | 0.014 | 1.81E-23 | 1.91E-04 | 99.657 |
| rs2543537 | C | T | 0.46 | -0.083 | 0.014 | 5.59E-09 | 6.53E-05 | 33.971 |
| rs4820827 | T | C | 0.62 | -0.130 | 0.014 | 1.01E-19 | 1.59E-04 | 82.590 |
| rs229527 | C | A | 0.42 | 0.104 | 0.014 | 1.82E-13 | 1.04E-04 | 54.192 |

**Abbreviation**

SNPs Single-nucleotide polymorphisms

EA Effect allele

NEA Non-effect allele

EAF Effect allele frequency

SE Standard error of the β

P-value Strength of evidence against the null hypothesis of no association between

variant and outcome

F Instrument strengh

R^2^ Proportion of variance in the exposure variable

β Per allele effect

**Supplementary Table 2**

Summary information of genetic instruments associated with brain tumor

| **SNPs** | **β** | **SE** | **P-value** | **EAF** | **EA** | **NEA** |
| --- | --- | --- | --- | --- | --- | --- |
| rs10751776 | 0.078 | 0.014 | 2.67E-08 | 0.1147 | A | C |
| rs855330 | 0.111 | 0.017 | 4.89E-11 | 0.4079 | G | C |
| rs12128789 | 0.127 | 0.022 | 3.73E-09 | 0.9597 | G | A |
| rs10801128 | 0.096 | 0.016 | 8.98E-10 | 0.1886 | G | C |
| rs17623914 | -0.135 | 0.023 | 7.97E-09 | 0.3288 | G | T |
| rs7511678 | 0.094 | 0.017 | 3.09E-08 | 0.1617 | T | G |
| rs12742756 | -0.083 | 0.015 | 3.54E-08 | 0.3117 | C | A |
| rs6679677 | 0.642 | 0.021 | 1.00E-200 | 0.3007 | G | A |
| rs3024493 | -0.164 | 0.020 | 7.26E-17 | 0.1261 | G | C |
| rs574384 | -0.134 | 0.024 | 2.20E-08 | 0.4734 | A | G |
| rs2493411 | 0.127 | 0.022 | 1.28E-08 | 0.8258 | A | C |
| rs3087243 | -0.199 | 0.014 | 1.16E-44 | 0.0963 | A | G |
| rs4490209 | -0.085 | 0.015 | 4.55E-08 | 0.4607 | C | G |
| rs1881146 | -0.095 | 0.017 | 4.57E-08 | 0.1484 | A | C |
| rs12464462 | -0.088 | 0.014 | 8.61E-10 | 0.7097 | A | C |
| rs2111485 | 0.128 | 0.014 | 1.05E-18 | 0.4080 | G | A |
| rs6434435 | -0.123 | 0.019 | 1.23E-10 | 0.3782 | A | G |
| rs55893453 | 0.095 | 0.017 | 4.63E-08 | 0.2627 | C | T |
| rs28648882 | 0.099 | 0.018 | 3.03E-08 | 0.1282 | T | C |
| rs7668577 | 0.094 | 0.015 | 7.26E-10 | 0.5550 | C | A |
| rs2611211 | -0.144 | 0.019 | 1.39E-14 | 0.4649 | G | A |
| rs12644686 | -0.108 | 0.019 | 2.44E-08 | 0.2907 | A | G |
| rs13147049 | -0.110 | 0.015 | 8.92E-14 | 0.5526 | G | A |
| rs2303137 | -0.082 | 0.014 | 6.17E-09 | 0.7642 | G | T |
| rs114378220 | 0.178 | 0.030 | 5.11E-09 | 0.0010 | T | A |
| rs2188962 | 0.079 | 0.014 | 1.73E-08 | 0.5239 | T | A |
| rs9260802 | -0.356 | 0.042 | 1.35E-17 | 0.2928 | G | C |
| rs7752257 | -0.680 | 0.017 | 1.00E-200 | 0.1548 | G | A |
| rs6908626 | 0.203 | 0.019 | 6.14E-28 | 0.7942 | T | A |
| rs28752526 | 0.630 | 0.016 | 1.00E-200 | 0.7181 | G | T |
| rs4548024 | -0.096 | 0.017 | 9.95E-09 | 0.4269 | C | T |
| rs1050979 | 0.106 | 0.014 | 5.65E-14 | 0.4650 | A | G |
| rs1611236 | -0.260 | 0.017 | 1.83E-50 | 0.4711 | C | T |
| rs3135348 | 1.048 | 0.017 | 1.00E-200 | 0.1156 | C | A |
| rs9468618 | -0.272 | 0.026 | 5.10E-25 | 0.0980 | C | T |
| rs2429557 | 1.021 | 0.058 | 4.98E-69 | 0.1172 | A | C |
| rs9385401 | 0.120 | 0.015 | 4.59E-16 | 0.3362 | T | A |
| rs238873 | 0.793 | 0.053 | 4.30E-50 | 0.1313 | C | T |
| rs73432769 | 0.335 | 0.046 | 2.34E-13 | 0.4238 | T | C |
| rs112733823 | 0.383 | 0.020 | 7.42E-81 | 0.7717 | T | C |
| rs1794269 | 1.594 | 0.018 | 1.00E-200 | 0.4895 | G | A |
| rs6908236 | 0.166 | 0.014 | 6.31E-32 | 0.3387 | G | A |
| rs7795896 | -0.135 | 0.016 | 1.58E-16 | 0.0337 | T | C |
| rs17323934 | -0.130 | 0.017 | 1.26E-14 | 0.2243 | T | C |
| rs10275896 | -0.121 | 0.017 | 2.60E-13 | 0.2936 | G | T |
| rs7776597 | 0.244 | 0.036 | 1.82E-11 | 0.3128 | A | G |
| rs10224046 | 0.086 | 0.015 | 2.71E-08 | 0.5387 | C | T |
| rs13259300 | -0.092 | 0.015 | 3.28E-10 | 0.0419 | C | G |
| rs1947178 | -0.103 | 0.017 | 1.67E-09 | 0.6684 | G | C |
| rs3802214 | -0.107 | 0.019 | 2.96E-08 | 0.2970 | G | T |
| rs1574285 | -0.127 | 0.014 | 4.27E-19 | 0.2497 | T | G |
| rs78325861 | -0.282 | 0.042 | 2.31E-11 | 0.1949 | C | T |
| rs12257077 | 0.231 | 0.037 | 3.91E-10 | 0.6338 | T | C |
| rs7068821 | -0.165 | 0.016 | 5.07E-24 | 0.1102 | C | T |
| rs61839660 | -0.357 | 0.026 | 5.25E-43 | 0.5405 | C | A |
| rs41295159 | -0.700 | 0.090 | 9.11E-15 | 0.6762 | G | A |
| rs722988 | 0.083 | 0.014 | 9.78E-09 | 0.0831 | T | C |
| rs7936434 | 0.077 | 0.014 | 3.58E-08 | 0.7246 | C | T |
| rs7130222 | -0.092 | 0.016 | 1.30E-08 | 0.5207 | C | A |
| rs663743 | -0.100 | 0.015 | 3.50E-11 | 0.1993 | C | T |
| rs607703 | 0.092 | 0.014 | 1.17E-10 | 0.5997 | G | A |
| rs689 | 0.712 | 0.019 | 1.00E-200 | 0.0143 | G | A |
| rs1701704 | 0.244 | 0.015 | 4.52E-63 | 0.2924 | T | C |
| rs7301381 | -0.094 | 0.014 | 5.25E-11 | 0.6615 | C | T |
| rs10844597 | -0.090 | 0.014 | 1.20E-10 | 0.1812 | T | C |
| rs3184504 | -0.231 | 0.014 | 1.08E-60 | 0.8540 | C | T |
| rs238265 | -0.091 | 0.015 | 2.08E-09 | 0.7690 | C | T |
| rs9517712 | -0.102 | 0.016 | 1.06E-10 | 0.6222 | G | T |
| rs17106304 | 0.115 | 0.015 | 6.83E-15 | 0.1884 | T | C |
| rs1350275 | -0.094 | 0.015 | 8.86E-10 | 0.3560 | C | G |
| rs56994090 | -0.134 | 0.015 | 3.60E-20 | 0.5343 | C | T |
| rs34593439 | -0.218 | 0.024 | 1.54E-19 | 0.0384 | G | C |
| rs12927355 | -0.204 | 0.015 | 4.41E-41 | 0.2241 | A | G |
| rs231972 | 0.171 | 0.021 | 5.12E-16 | 0.1556 | A | G |
| rs55993634 | 0.219 | 0.024 | 2.29E-19 | 0.2914 | A | G |
| rs8046043 | -0.085 | 0.015 | 2.49E-08 | 0.0694 | T | C |
| rs57209021 | 0.101 | 0.018 | 3.73E-08 | 0.7676 | T | C |
| rs61759532 | 0.118 | 0.019 | 1.91E-10 | 0.0090 | A | T |
| rs35327136 | -0.119 | 0.019 | 3.37E-10 | 0.0358 | G | A |
| rs1808094 | -0.114 | 0.015 | 2.40E-14 | 0.0721 | T | C |
| rs7237497 | -0.220 | 0.019 | 2.71E-32 | 0.5828 | T | G |
| rs34536443 | -0.385 | 0.039 | 1.47E-23 | 0.8130 | G | A |
| rs601338 | 0.127 | 0.014 | 1.20E-18 | 0.0309 | T | C |
| rs113374757 | -0.171 | 0.021 | 1.63E-16 | 0.0085 | G | C |
| rs202535 | -0.141 | 0.018 | 1.79E-14 | 0.4449 | C | T |
| rs11203203 | 0.144 | 0.014 | 1.81E-23 | 0.4676 | T | C |
| rs2543537 | -0.083 | 0.014 | 5.59E-09 | 0.2746 | T | C |
| rs4820827 | -0.130 | 0.014 | 1.01E-19 | 0.2613 | A | G |
| rs229527 | 0.104 | 0.014 | 1.82E-13 | 0.4562 | T | C |

**Supplementary Table 3**

Summary information of genetic instruments associated with breast cancer

| **SNP** | **Beta** | **SE** | **P-value** | **EAF** | **EA** | **NEA** |
| --- | --- | --- | --- | --- | --- | --- |
| rs7795896 | -0.0072 | 0.0110 | 0.513 | 0.6942 | T | C |
| rs41295159 | 0.0021 | 0.0519 | 0.968 | 0.0110 | G | C |
| rs1881146 | -0.0211 | 0.0110 | 0.054 | 0.3156 | T | A |
| rs2611211 | 0.0073 | 0.0137 | 0.591 | 0.8316 | T | C |
| rs6908236 | -0.0119 | 0.0101 | 0.239 | 0.5085 | C | A |
| rs73432769 | -0.0377 | 0.0342 | 0.271 | 0.0232 | T | C |
| rs2429557 | 0.0789 | 0.0483 | 0.102 | 0.0104 | A | T |
| rs8046043 | -0.0080 | 0.0104 | 0.438 | 0.3982 | C | G |
| rs1947178 | 0.0134 | 0.0121 | 0.270 | 0.7729 | G | A |
| rs34536443 | 0.0054 | 0.0251 | 0.829 | 0.0424 | C | G |
| rs607703 | -0.0033 | 0.0102 | 0.743 | 0.4728 | T | C |
| rs9517712 | 0.0047 | 0.0119 | 0.695 | 0.7565 | C | T |
| rs10751776 | 0.0270 | 0.0101 | 0.008 | 0.4884 | C | A |
| rs6908626 | -0.0098 | 0.0152 | 0.518 | 0.1647 | T | G |
| rs9260802 | -0.0047 | 0.0272 | 0.861 | 0.0356 | G | A |
| rs12644686 | -0.0097 | 0.0138 | 0.482 | 0.1826 | G | C |
| rs2188962 | 0.0312 | 0.0103 | 0.002 | 0.4156 | T | C |
| rs855330 | -0.0023 | 0.0121 | 0.850 | 0.2345 | C | T |
| rs12128789 | -0.0295 | 0.0155 | 0.057 | 0.1264 | C | T |
| rs2493411 | 0.0183 | 0.0154 | 0.233 | 0.1242 | C | T |
| rs574384 | -0.0103 | 0.0166 | 0.535 | 0.8971 | A | C |
| rs56994090 | 0.0315 | 0.0111 | 0.005 | 0.4095 | C | T |
| rs17323934 | 0.0052 | 0.0122 | 0.672 | 0.2251 | G | C |
| rs1574285 | 0.0077 | 0.0104 | 0.458 | 0.5770 | T | G |
| rs35327136 | -0.0458 | 0.0132 | 0.001 | 0.1774 | A | C |
| rs7068821 | 0.0240 | 0.0117 | 0.041 | 0.2531 | T | G |
| rs34593439 | 0.0149 | 0.0167 | 0.372 | 0.1007 | A | G |
| rs229527 | -0.0044 | 0.0102 | 0.671 | 0.4175 | A | C |
| rs3135348 | -0.0150 | 0.0103 | 0.147 | 0.5871 | G | A |
| rs7752257 | 0.0196 | 0.0118 | 0.096 | 0.7459 | G | T |
| rs2111485 | -0.0106 | 0.0103 | 0.306 | 0.6077 | G | A |
| rs722988 | 0.0046 | 0.0104 | 0.660 | 0.3742 | C | T |
| rs12257077 | 0.0138 | 0.0259 | 0.593 | 0.0408 | T | C |
| rs7936434 | 0.0099 | 0.0102 | 0.330 | 0.4735 | C | G |
| rs7776597 | 0.0023 | 0.0262 | 0.931 | 0.9588 | G | A |
| rs1350275 | -0.0147 | 0.0111 | 0.188 | 0.7112 | G | T |
| rs6679677 | 0.0329 | 0.0175 | 0.061 | 0.0952 | A | C |
| rs17623914 | 0.0248 | 0.0165 | 0.134 | 0.1034 | C | T |
| rs4548024 | -0.0128 | 0.0125 | 0.304 | 0.2275 | C | T |
| rs13147049 | 0.0023 | 0.0107 | 0.831 | 0.6571 | G | A |
| rs238265 | -0.0024 | 0.0112 | 0.829 | 0.7045 | G | T |
| rs2543537 | 0.0100 | 0.0106 | 0.345 | 0.4274 | T | C |
| rs601338 | 0.0130 | 0.0102 | 0.202 | 0.4626 | A | G |
| rs231972 | 0.0091 | 0.0156 | 0.560 | 0.1204 | C | A |
| rs3087243 | 0.0043 | 0.0104 | 0.678 | 0.4352 | A | G |
| rs7668577 | -0.0143 | 0.0111 | 0.194 | 0.3106 | C | A |
| rs3024493 | 0.0078 | 0.0139 | 0.575 | 0.1569 | A | C |
| rs4490209 | 0.0012 | 0.0105 | 0.908 | 0.3557 | G | C |
| rs28648882 | 0.0037 | 0.0118 | 0.755 | 0.2364 | A | G |
| rs55893453 | -0.0208 | 0.0127 | 0.101 | 0.2009 | G | A |
| rs6434435 | -0.0116 | 0.0134 | 0.385 | 0.1772 | A | G |
| rs12464462 | -0.0182 | 0.0104 | 0.078 | 0.4078 | G | A |
| rs238873 | -0.0087 | 0.0465 | 0.851 | 0.0119 | G | A |
| rs1794269 | -0.0174 | 0.0105 | 0.099 | 0.3755 | T | C |
| rs9385401 | 0.0083 | 0.0101 | 0.411 | 0.4668 | T | C |
| rs10801128 | -0.0105 | 0.0112 | 0.349 | 0.7148 | G | A |
| rs12742756 | -0.0020 | 0.0102 | 0.845 | 0.4327 | G | A |
| rs2303137 | 0.0008 | 0.0102 | 0.938 | 0.4354 | T | A |
| rs202535 | 0.0096 | 0.0134 | 0.475 | 0.8260 | A | C |
| rs113374757 | -0.0100 | 0.0140 | 0.475 | 0.1594 | T | C |
| rs78325861 | 0.0098 | 0.0265 | 0.711 | 0.0438 | G | C |
| rs4820827 | -0.0168 | 0.0104 | 0.107 | 0.6242 | C | T |
| rs11203203 | 0.0029 | 0.0105 | 0.780 | 0.3718 | A | G |
| rs57209021 | 0.0168 | 0.0121 | 0.165 | 0.2327 | T | C |
| rs55993634 | -0.0115 | 0.0183 | 0.532 | 0.0894 | G | C |
| rs10224046 | -0.0068 | 0.0109 | 0.534 | 0.3237 | G | T |
| rs7130222 | -0.0177 | 0.0108 | 0.104 | 0.3203 | G | T |
| rs663743 | 0.0012 | 0.0108 | 0.910 | 0.3349 | A | G |
| rs13259300 | -0.0180 | 0.0107 | 0.092 | 0.6308 | C | A |
| rs17106304 | -0.0115 | 0.0107 | 0.284 | 0.6664 | G | C |
| rs12927355 | 0.0052 | 0.0108 | 0.629 | 0.3253 | T | C |
| rs28752526 | -0.0075 | 0.0108 | 0.483 | 0.3306 | G | A |
| rs1050979 | 0.0049 | 0.0102 | 0.630 | 0.4870 | G | A |
| rs114378220 | -0.0038 | 0.0250 | 0.879 | 0.0579 | T | C |
| rs689 | -0.0105 | 0.0112 | 0.351 | 0.7205 | T | A |
| rs7237497 | -0.0244 | 0.0139 | 0.079 | 0.8402 | C | T |
| rs61759532 | -0.0032 | 0.0136 | 0.815 | 0.2090 | T | C |
| rs1808094 | -0.0073 | 0.0101 | 0.474 | 0.5246 | C | T |
| rs7511678 | 0.0108 | 0.0125 | 0.385 | 0.2074 | A | G |
| rs3802214 | 0.0047 | 0.0132 | 0.720 | 0.8137 | C | T |
| rs61839660 | -0.0228 | 0.0178 | 0.201 | 0.0883 | T | C |
| rs1611236 | 0.0074 | 0.0115 | 0.519 | 0.3269 | A | G |
| rs112733823 | -0.0400 | 0.0158 | 0.011 | 0.1319 | T | C |
| rs10275896 | 0.0070 | 0.0119 | 0.559 | 0.2339 | C | T |

**Supplementary Table 4**

Summary information of genetic instruments associated with cervical cancer

| **SNP** | **Beta** | **SE** | **p value** | **EAF** | **EA** | **NEA** |
| --- | --- | --- | --- | --- | --- | --- |
| rs2493411 | 2.16E-05 | 2.55E-04 | 0.930 | 0.124 | C | T |
| rs2111485 | -2.21E-04 | 1.72E-04 | 0.200 | 0.607 | G | A |
| rs855330 | -1.25E-04 | 2.00E-04 | 0.530 | 0.233 | C | T |
| rs12644686 | 1.78E-04 | 2.22E-04 | 0.420 | 0.179 | G | C |
| rs9517712 | -1.59E-04 | 1.94E-04 | 0.410 | 0.749 | C | T |
| rs9260802 | 3.78E-04 | 4.57E-04 | 0.410 | 0.035 | G | A |
| rs7752257 | -3.28E-04 | 1.93E-04 | 0.090 | 0.742 | G | T |
| rs6908236 | -2.09E-04 | 1.69E-04 | 0.220 | 0.530 | C | A |
| rs6908626 | 1.01E-04 | 2.21E-04 | 0.650 | 0.178 | T | G |
| rs10275896 | 2.03E-04 | 1.96E-04 | 0.300 | 0.245 | C | T |
| rs663743 | -9.63E-06 | 1.78E-04 | 0.960 | 0.340 | A | G |
| rs7936434 | -1.50E-04 | 1.68E-04 | 0.370 | 0.477 | C | G |
| rs12464462 | -6.88E-05 | 1.71E-04 | 0.690 | 0.415 | G | A |
| rs17106304 | 3.56E-05 | 1.77E-04 | 0.840 | 0.657 | G | C |
| rs1350275 | -1.92E-04 | 1.88E-04 | 0.310 | 0.721 | G | T |
| rs56994090 | -9.75E-05 | 1.71E-04 | 0.570 | 0.416 | C | T |
| rs12927355 | -1.23E-04 | 1.80E-04 | 0.500 | 0.322 | T | C |
| rs55993634 | 2.65E-04 | 3.11E-04 | 0.390 | 0.083 | G | C |
| rs8046043 | 1.38E-04 | 1.71E-04 | 0.420 | 0.416 | C | G |
| rs1808094 | 3.22E-05 | 1.69E-04 | 0.850 | 0.526 | C | T |
| rs112733823 | 2.23E-04 | 2.62E-04 | 0.390 | 0.116 | T | C |
| rs61839660 | 2.47E-04 | 2.83E-04 | 0.380 | 0.098 | T | C |
| rs4490209 | -1.43E-04 | 1.75E-04 | 0.410 | 0.363 | G | C |
| rs7511678 | 4.96E-06 | 2.10E-04 | 0.980 | 0.203 | A | G |
| rs1881146 | -1.63E-04 | 1.83E-04 | 0.370 | 0.312 | T | A |
| rs4548024 | 6.78E-05 | 2.00E-04 | 0.730 | 0.237 | C | T |
| rs689 | 1.13E-04 | 1.87E-04 | 0.550 | 0.712 | T | A |
| rs607703 | 8.43E-05 | 1.69E-04 | 0.620 | 0.474 | T | C |
| rs1701704 | -2.53E-04 | 1.77E-04 | 0.150 | 0.342 | G | T |
| rs34536443 | -4.03E-04 | 4.16E-04 | 0.330 | 0.045 | C | G |
| rs601338 | 4.10E-04 | 1.68E-04 | 0.015 | 0.508 | A | G |
| rs229527 | 5.31E-05 | 1.70E-04 | 0.760 | 0.424 | A | C |
| rs1611236 | 1.24E-04 | 1.80E-04 | 0.490 | 0.324 | A | G |
| rs9468618 | -2.77E-04 | 2.95E-04 | 0.350 | 0.090 | T | C |
| rs10224046 | 1.10E-04 | 1.82E-04 | 0.550 | 0.310 | G | T |
| rs13259300 | 1.08E-04 | 1.74E-04 | 0.540 | 0.612 | C | A |
| rs7068821 | -1.18E-04 | 1.93E-04 | 0.540 | 0.256 | T | G |
| rs7301381 | 3.06E-04 | 1.69E-04 | 0.070 | 0.464 | C | T |
| rs231972 | 7.18E-05 | 2.55E-04 | 0.780 | 0.125 | C | A |
| rs57209021 | 1.59E-05 | 2.01E-04 | 0.940 | 0.231 | T | C |
| rs7237497 | 6.83E-05 | 2.27E-04 | 0.760 | 0.835 | C | T |
| rs6679677 | 5.34E-04 | 2.79E-04 | 0.056 | 0.101 | A | C |
| rs238265 | -2.32E-04 | 1.85E-04 | 0.210 | 0.702 | G | T |
| rs574384 | -3.32E-04 | 2.87E-04 | 0.250 | 0.904 | A | C |
| rs13147049 | 1.98E-04 | 1.79E-04 | 0.270 | 0.671 | G | A |
| rs17623914 | 2.86E-04 | 2.73E-04 | 0.290 | 0.109 | C | T |
| rs7668577 | -5.86E-05 | 1.82E-04 | 0.750 | 0.311 | C | A |
| rs6434435 | -9.98E-05 | 2.25E-04 | 0.660 | 0.176 | A | G |
| rs11203203 | -2.18E-04 | 1.75E-04 | 0.210 | 0.367 | A | G |
| rs202535 | 2.38E-04 | 2.30E-04 | 0.300 | 0.840 | A | C |
| rs12128789 | 2.13E-04 | 2.45E-04 | 0.380 | 0.138 | C | T |
| rs722988 | -2.89E-05 | 1.75E-04 | 0.870 | 0.365 | C | T |
| rs78325861 | 2.57E-05 | 4.27E-04 | 0.950 | 0.041 | G | C |
| rs34593439 | -1.72E-04 | 2.77E-04 | 0.540 | 0.103 | A | G |
| rs113374757 | 2.26E-04 | 2.29E-04 | 0.320 | 0.163 | T | C |
| rs114378220 | -1.31E-04 | 3.35E-04 | 0.700 | 0.074 | T | C |
| rs7795896 | -1.18E-04 | 1.85E-04 | 0.520 | 0.706 | T | C |
| rs1947178 | 1.93E-04 | 2.03E-04 | 0.340 | 0.775 | G | A |
| rs1574285 | 3.77E-05 | 1.72E-04 | 0.830 | 0.599 | T | G |
| rs3087243 | -1.33E-04 | 1.69E-04 | 0.430 | 0.451 | A | G |
| rs28648882 | 2.87E-04 | 2.02E-04 | 0.150 | 0.225 | A | G |
| rs12257077 | 1.79E-04 | 4.47E-04 | 0.690 | 0.037 | T | C |
| rs2611211 | -3.98E-05 | 2.31E-04 | 0.860 | 0.842 | T | C |
| rs2303137 | -2.03E-05 | 1.70E-04 | 0.900 | 0.432 | T | A |
| rs7776597 | 2.23E-04 | 4.07E-04 | 0.580 | 0.955 | G | A |
| rs2188962 | 6.84E-05 | 1.70E-04 | 0.690 | 0.426 | T | C |
| rs1050979 | -2.00E-04 | 1.68E-04 | 0.230 | 0.527 | G | A |
| rs3135348 | 8.67E-05 | 1.69E-04 | 0.610 | 0.560 | G | A |
| rs10751776 | -2.51E-05 | 1.69E-04 | 0.880 | 0.501 | C | A |
| rs10801128 | 1.14E-04 | 1.85E-04 | 0.540 | 0.710 | G | A |
| rs3024493 | -2.12E-04 | 2.34E-04 | 0.360 | 0.154 | A | C |
| rs55893453 | -3.20E-05 | 2.06E-04 | 0.880 | 0.214 | G | A |
| rs12742756 | 7.51E-05 | 1.70E-04 | 0.660 | 0.427 | G | A |
| rs3802214 | 1.13E-04 | 2.19E-04 | 0.600 | 0.818 | C | T |
| rs7130222 | -2.46E-04 | 1.80E-04 | 0.170 | 0.325 | G | T |
| rs3184504 | -7.87E-06 | 1.68E-04 | 0.960 | 0.516 | C | T |
| rs61759532 | 2.20E-04 | 2.00E-04 | 0.270 | 0.247 | T | C |
| rs35327136 | -3.78E-04 | 2.17E-04 | 0.081 | 0.186 | A | C |
| rs4820827 | 2.73E-05 | 1.72E-04 | 0.870 | 0.606 | C | T |
| rs9385401 | 7.31E-05 | 1.69E-04 | 0.670 | 0.457 | T | C |
| rs17323934 | 1.81E-04 | 1.97E-04 | 0.360 | 0.242 | G | C |
| rs2543537 | -4.06E-05 | 1.71E-04 | 0.810 | 0.435 | T | C |

**Supplementary Table 5**

Summary information of genetic instruments associated with cholangiocarcinoma

| **SNP** | **Beta** | **SE** | **p-value** | **EAF** | **EA** | **NEA** |
| --- | --- | --- | --- | --- | --- | --- |
| rs574384 | -0.0152 | 0.0602 | 0.800 | 0.7204 | A | C |
| rs12742756 | -0.0239 | 0.0412 | 0.562 | 0.4099 | G | A |
| rs6679677 | 0.0291 | 0.0786 | 0.711 | 0.1152 | A | C |
| rs17623914 | -0.0748 | 0.0826 | 0.365 | 0.0977 | C | T |
| rs12464462 | -0.0844 | 0.0502 | 0.092 | 0.3069 | G | A |
| rs2111485 | 0.0589 | 0.0439 | 0.180 | 0.4959 | G | A |
| rs6434435 | 0.0214 | 0.0556 | 0.700 | 0.1551 | A | G |
| rs9468618 | -0.1332 | 0.0779 | 0.087 | 0.0724 | T | C |
| rs2611211 | -0.0605 | 0.0484 | 0.211 | 0.7703 | T | C |
| rs9385401 | -0.0181 | 0.0544 | 0.739 | 0.6257 | T | C |
| rs4548024 | -0.0190 | 0.0522 | 0.716 | 0.2011 | C | T |
| rs1947178 | 0.0277 | 0.0519 | 0.594 | 0.8126 | G | A |
| rs1881146 | 0.0252 | 0.0459 | 0.583 | 0.3353 | T | A |
| rs13147049 | -0.0361 | 0.0416 | 0.386 | 0.6009 | G | A |
| rs6908236 | 0.0065 | 0.0404 | 0.871 | 0.5193 | C | A |
| rs7301381 | -0.0235 | 0.0403 | 0.560 | 0.4707 | C | T |
| rs56994090 | 0.0121 | 0.0409 | 0.766 | 0.4283 | C | T |
| rs113374757 | 0.0877 | 0.0561 | 0.118 | 0.1804 | T | C |
| rs1808094 | -0.0337 | 0.0432 | 0.435 | 0.5335 | C | T |
| rs7936434 | 0.0430 | 0.0495 | 0.385 | 0.4603 | C | G |
| rs1701704 | 0.0408 | 0.0449 | 0.363 | 0.2993 | G | T |
| rs61839660 | -0.0742 | 0.0873 | 0.395 | 0.0826 | T | C |
| rs9517712 | 0.0199 | 0.0488 | 0.684 | 0.7671 | C | T |
| rs2543537 | 0.0084 | 0.0407 | 0.837 | 0.4572 | T | C |
| rs2429557 | 0.2392 | 0.2091 | 0.253 | 0.0090 | A | T |
| rs2303137 | -0.0172 | 0.0421 | 0.683 | 0.5196 | T | A |
| rs28648882 | -0.1091 | 0.0657 | 0.097 | 0.2240 | A | G |
| rs663743 | 0.0615 | 0.0444 | 0.166 | 0.3154 | A | G |
| rs3184504 | 0.1140 | 0.0496 | 0.021 | 0.6548 | C | T |
| rs78325861 | 0.2829 | 0.1285 | 0.028 | 0.0384 | G | C |
| rs13259300 | 0.0118 | 0.0412 | 0.775 | 0.5571 | C | A |
| rs10844597 | 0.0137 | 0.0408 | 0.738 | 0.4678 | A | G |
| rs238265 | -0.0163 | 0.0454 | 0.720 | 0.7164 | G | T |
| rs7795896 | -0.1012 | 0.0535 | 0.059 | 0.7666 | T | C |
| rs28752526 | -0.0006 | 0.0434 | 0.990 | 0.3391 | G | A |
| rs3024493 | -0.0095 | 0.0665 | 0.887 | 0.1193 | A | C |
| rs3802214 | 0.0555 | 0.0463 | 0.230 | 0.7280 | C | T |
| rs3135348 | 0.0364 | 0.0512 | 0.477 | 0.5523 | G | A |
| rs8046043 | -0.0112 | 0.0425 | 0.792 | 0.3571 | C | G |
| rs4490209 | -0.0230 | 0.0440 | 0.602 | 0.4058 | G | C |
| rs2493411 | -0.0957 | 0.0675 | 0.157 | 0.1119 | C | T |
| rs55893453 | 0.0528 | 0.0588 | 0.369 | 0.1573 | G | A |
| rs7511678 | 0.0106 | 0.0453 | 0.815 | 0.2889 | A | G |
| rs10801128 | 0.0709 | 0.0434 | 0.102 | 0.6789 | G | A |
| rs855330 | -0.0052 | 0.0466 | 0.910 | 0.2640 | C | T |
| rs2188962 | -0.0999 | 0.0504 | 0.047 | 0.2978 | T | C |
| rs17323934 | 0.0615 | 0.0527 | 0.243 | 0.1901 | G | C |
| rs689 | -0.0917 | 0.0534 | 0.086 | 0.7910 | T | A |
| rs7068821 | 0.0488 | 0.0464 | 0.293 | 0.2494 | T | G |
| rs7237497 | 0.0590 | 0.0575 | 0.305 | 0.8534 | C | T |
| rs11203203 | 0.0447 | 0.0498 | 0.370 | 0.2653 | A | G |
| rs231972 | -0.0658 | 0.0764 | 0.389 | 0.1154 | C | A |
| rs55993634 | 0.0461 | 0.0616 | 0.454 | 0.1237 | G | C |
| rs35327136 | -0.0621 | 0.0685 | 0.364 | 0.1472 | A | C |
| rs4820827 | 0.0580 | 0.0422 | 0.170 | 0.5444 | C | T |
| rs601338 | 0.0138 | 0.0498 | 0.782 | 0.4719 | A | G |
| rs202535 | -0.0425 | 0.0658 | 0.518 | 0.8254 | A | C |
| rs3087243 | 0.0597 | 0.0422 | 0.156 | 0.3797 | A | G |
| rs10751776 | 0.0239 | 0.0405 | 0.556 | 0.5394 | C | A |
| rs7668577 | 0.0126 | 0.0532 | 0.814 | 0.3117 | C | A |
| rs722988 | 0.0077 | 0.0428 | 0.857 | 0.4390 | C | T |
| rs6908626 | 0.0292 | 0.0667 | 0.661 | 0.1610 | T | G |
| rs10224046 | -0.0242 | 0.0529 | 0.647 | 0.3296 | G | T |
| rs7752257 | 0.1319 | 0.0481 | 0.006 | 0.7631 | G | T |
| rs9260802 | 0.0494 | 0.1328 | 0.710 | 0.0358 | G | A |
| rs7776597 | 0.0756 | 0.1233 | 0.540 | 0.9600 | G | A |
| rs1050979 | -0.0059 | 0.0411 | 0.885 | 0.4674 | G | A |
| rs73432769 | -0.0835 | 0.1061 | 0.431 | 0.0328 | T | C |
| rs34593439 | 0.1285 | 0.0703 | 0.068 | 0.0973 | A | G |
| rs34536443 | -0.2981 | 0.1243 | 0.017 | 0.0418 | C | G |
| rs229527 | -0.0511 | 0.0412 | 0.215 | 0.4758 | A | C |
| rs12927355 | 0.0297 | 0.0462 | 0.520 | 0.2769 | T | C |
| rs12128789 | 0.0727 | 0.0727 | 0.317 | 0.1311 | C | T |
| rs3135348 | 2.4717 | 1.8739 | 0.187 | 0.0010 | T | A |
| rs1794269 | -0.0396 | 0.0415 | 0.340 | 0.4249 | T | C |
| rs1611236 | -0.0119 | 0.0445 | 0.790 | 0.2902 | A | G |
| rs112733823 | 0.0175 | 0.0635 | 0.783 | 0.1299 | T | C |
| rs114378220 | 0.0329 | 0.0994 | 0.741 | 0.0692 | T | C |
| rs238873 | 0.0986 | 0.2083 | 0.636 | 0.0143 | G | A |
| rs10275896 | 0.0491 | 0.0521 | 0.346 | 0.1964 | C | T |
| rs12644686 | -0.0216 | 0.0484 | 0.656 | 0.2876 | G | C |
| rs12257077 | 0.0928 | 0.1403 | 0.508 | 0.0307 | T | C |
| rs41295159 | 0.1923 | 0.2599 | 0.460 | 0.0085 | G | C |
| rs7130222 | 0.0147 | 0.0443 | 0.740 | 0.2940 | G | T |
| rs1574285 | 0.0158 | 0.0410 | 0.700 | 0.5829 | T | G |
| rs607703 | 0.0078 | 0.0404 | 0.846 | 0.4692 | T | C |
| rs17106304 | -0.0089 | 0.0428 | 0.835 | 0.6676 | G | C |
| rs57209021 | 0.0257 | 0.0593 | 0.665 | 0.2242 | T | C |
| rs1350275 | 0.0030 | 0.0429 | 0.945 | 0.6256 | G | T |
| rs61759532 | -0.0659 | 0.0559 | 0.238 | 0.1904 | T | C |

**Supplementary Table 6**

Summary information of genetic instruments associated with colorectal cancer

| **SNP** | **Beta** | **SE** | **p-value** | **EAF** | **EA** | **NEA** |
| --- | --- | --- | --- | --- | --- | --- |
| rs1611236 | -0.0131 | 0.0181 | 0.468 | 0.3275 | A | G |
| rs10275896 | 0.0237 | 0.0199 | 0.233 | 0.2191 | C | T |
| rs55893453 | 0.0079 | 0.0211 | 0.709 | 0.1765 | G | A |
| rs3087243 | 0.0232 | 0.0173 | 0.179 | 0.4162 | A | G |
| rs10751776 | 0.0061 | 0.0169 | 0.720 | 0.5255 | C | A |
| rs1881146 | 0.0181 | 0.0187 | 0.332 | 0.3219 | T | A |
| rs10801128 | 0.0236 | 0.0188 | 0.210 | 0.6952 | G | A |
| rs1947178 | -0.0308 | 0.0199 | 0.121 | 0.7644 | G | A |
| rs722988 | 0.0041 | 0.0174 | 0.813 | 0.4419 | C | T |
| rs7795896 | -0.0208 | 0.0188 | 0.269 | 0.7507 | T | C |
| rs78325861 | -0.0262 | 0.0453 | 0.563 | 0.0352 | G | C |
| rs61839660 | -0.0437 | 0.0307 | 0.155 | 0.0717 | T | C |
| rs1350275 | -0.0070 | 0.0187 | 0.707 | 0.6693 | G | T |
| rs3184504 | 0.0753 | 0.0171 | 0.000 | 0.5799 | C | T |
| rs8046043 | 0.0061 | 0.0174 | 0.728 | 0.3768 | C | G |
| rs61759532 | -0.0099 | 0.0226 | 0.663 | 0.1900 | T | C |
| rs855330 | 0.0187 | 0.0202 | 0.353 | 0.2370 | C | T |
| rs6679677 | 0.0192 | 0.0311 | 0.538 | 0.0743 | A | C |
| rs7776597 | 0.0550 | 0.0468 | 0.240 | 0.9693 | G | A |
| rs9385401 | -0.0306 | 0.0170 | 0.072 | 0.5523 | T | C |
| rs3135348 | 0.0479 | 0.0174 | 0.006 | 0.6044 | G | A |
| rs1050979 | -0.0259 | 0.0169 | 0.126 | 0.4727 | G | A |
| rs73432769 | 0.0388 | 0.0499 | 0.437 | 0.0350 | T | C |
| rs2429557 | 0.0040 | 0.0816 | 0.961 | 0.0196 | A | T |
| rs3802214 | 0.0042 | 0.0221 | 0.849 | 0.7733 | C | T |
| rs10844597 | 0.0065 | 0.0169 | 0.702 | 0.4985 | A | G |
| rs7068821 | 0.0115 | 0.0199 | 0.564 | 0.2399 | T | G |
| rs9517712 | 0.0125 | 0.0195 | 0.523 | 0.7662 | C | T |
| rs689 | 0.0019 | 0.0189 | 0.921 | 0.7582 | T | A |
| rs34593439 | -0.0027 | 0.0281 | 0.925 | 0.0946 | A | G |
| rs1701704 | -0.0163 | 0.0181 | 0.368 | 0.3067 | G | T |
| rs238265 | -0.0212 | 0.0188 | 0.261 | 0.7295 | G | T |
| rs7237497 | -0.0090 | 0.0243 | 0.711 | 0.8518 | C | T |
| rs601338 | 0.0090 | 0.0170 | 0.597 | 0.4108 | A | G |
| rs55993634 | 0.0314 | 0.0307 | 0.306 | 0.1018 | G | C |
| rs2543537 | 0.0024 | 0.0175 | 0.892 | 0.4281 | T | C |
| rs229527 | -0.0317 | 0.0172 | 0.065 | 0.4759 | A | C |
| rs2111485 | -0.0059 | 0.0173 | 0.732 | 0.5186 | G | A |
| rs12464462 | 0.0023 | 0.0176 | 0.896 | 0.3244 | G | A |
| rs12644686 | 0.0147 | 0.0218 | 0.501 | 0.2380 | G | C |
| rs6908626 | -0.0025 | 0.0253 | 0.922 | 0.1342 | T | G |
| rs238873 | 0.0452 | 0.0772 | 0.558 | 0.0108 | G | A |
| rs2188962 | -0.0095 | 0.0172 | 0.581 | 0.3545 | T | C |
| rs17106304 | 0.0255 | 0.0181 | 0.160 | 0.6755 | G | C |
| rs12742756 | -0.0204 | 0.0170 | 0.231 | 0.4223 | G | A |
| rs7511678 | 0.0071 | 0.0210 | 0.736 | 0.2562 | A | G |
| rs28648882 | -0.0155 | 0.0197 | 0.431 | 0.2086 | A | G |
| rs2611211 | -0.0353 | 0.0224 | 0.115 | 0.7941 | T | C |
| rs7668577 | 0.0085 | 0.0190 | 0.655 | 0.2628 | C | A |
| rs9260802 | -0.0125 | 0.0469 | 0.790 | 0.0308 | G | A |
| rs4490209 | 0.0021 | 0.0176 | 0.905 | 0.4092 | G | C |
| rs17323934 | 0.0183 | 0.0203 | 0.368 | 0.2123 | G | C |
| rs12257077 | 0.0683 | 0.0430 | 0.112 | 0.0377 | T | C |
| rs663743 | 0.0585 | 0.0181 | 0.001 | 0.3144 | A | G |
| rs41295159 | 0.0306 | 0.1012 | 0.762 | 0.0071 | G | C |
| rs12927355 | 0.0119 | 0.0179 | 0.506 | 0.3167 | T | C |
| rs35327136 | 0.0011 | 0.0219 | 0.959 | 0.1602 | A | C |
| rs231972 | -0.0239 | 0.0253 | 0.345 | 0.1160 | C | A |
| rs202535 | 0.0306 | 0.0221 | 0.166 | 0.8499 | A | C |
| rs607703 | 0.0123 | 0.0170 | 0.468 | 0.4567 | T | C |
| rs57209021 | 0.0179 | 0.0214 | 0.405 | 0.3836 | T | C |
| rs34536443 | 0.0399 | 0.0442 | 0.366 | 0.0348 | C | G |
| rs7936434 | 0.0105 | 0.0169 | 0.535 | 0.4824 | C | G |
| rs574384 | -0.0333 | 0.0262 | 0.204 | 0.7756 | A | C |
| rs6434435 | -0.0001 | 0.0220 | 0.995 | 0.1814 | A | G |
| rs2493411 | 0.0325 | 0.0260 | 0.211 | 0.1157 | C | T |
| rs13147049 | -0.0128 | 0.0185 | 0.490 | 0.6581 | G | A |
| rs2303137 | 0.0046 | 0.0171 | 0.790 | 0.4755 | T | A |
| rs17623914 | 0.0084 | 0.0270 | 0.756 | 0.0957 | C | T |
| rs3024493 | 0.0209 | 0.0237 | 0.376 | 0.1349 | A | C |
| rs12128789 | -0.0224 | 0.0267 | 0.402 | 0.1373 | C | T |
| rs1574285 | -0.0284 | 0.0175 | 0.103 | 0.5777 | T | G |
| rs13259300 | -0.0035 | 0.0182 | 0.847 | 0.6315 | C | A |
| rs114378220 | -0.0057 | 0.0373 | 0.879 | 0.0662 | T | C |
| rs10224046 | -0.0070 | 0.0183 | 0.704 | 0.2729 | G | T |
| rs6908236 | 0.0086 | 0.0170 | 0.611 | 0.5055 | C | A |
| rs9468618 | -0.0108 | 0.0294 | 0.713 | 0.0901 | T | C |
| rs56994090 | -0.0005 | 0.0186 | 0.979 | 0.3987 | C | T |
| rs7301381 | -0.0004 | 0.0170 | 0.983 | 0.4454 | C | T |
| rs7130222 | -0.0003 | 0.0181 | 0.986 | 0.3168 | G | T |
| rs113374757 | 0.0106 | 0.0261 | 0.685 | 0.1413 | T | C |
| rs11203203 | -0.0255 | 0.0176 | 0.147 | 0.3332 | A | G |
| rs4820827 | -0.0017 | 0.0176 | 0.922 | 0.5879 | C | T |
| rs7752257 | -0.0280 | 0.0203 | 0.168 | 0.7754 | G | T |
| rs28752526 | 0.0075 | 0.0179 | 0.675 | 0.3280 | G | A |
| rs112733823 | -0.0084 | 0.0274 | 0.759 | 0.1050 | T | C |
| rs1808094 | -0.0639 | 0.0170 | 0.000 | 0.5379 | C | T |

**Supplementary Table 7**

Summary information of genetic instruments associated with endometrial cancer

| **SNP** | **Beta** | **SE** | **p-value** | **EAF** | **EA** | **NEA** |
| --- | --- | --- | --- | --- | --- | --- |
| rs7668577 | 8.21E-03 | 1.67E-02 | 0.622 | 0.309565 | C | A |
| rs9468618 | 3.24E-02 | 2.68E-02 | 0.227 | 0.0899499 | T | C |
| rs73432769 | -1.05E-02 | 5.40E-02 | 0.845 | 0.0219461 | T | C |
| rs112733823 | -7.81E-03 | 2.35E-02 | 0.740 | 0.127072 | T | C |
| rs574384 | -4.40E-02 | 2.54E-02 | 0.083 | 0.900036 | A | C |
| rs13147049 | -1.55E-02 | 1.60E-02 | 0.332 | 0.657688 | G | A |
| rs6679677 | 7.77E-03 | 2.60E-02 | 0.765 | 0.099552 | A | C |
| rs1881146 | 2.49E-02 | 1.68E-02 | 0.138 | 0.310432 | T | A |
| rs55893453 | 1.09E-02 | 1.92E-02 | 0.568 | 0.206144 | G | A |
| rs607703 | -1.02E-02 | 1.53E-02 | 0.508 | 0.474697 | T | C |
| rs9517712 | -2.74E-02 | 1.77E-02 | 0.122 | 0.754556 | C | T |
| rs12742756 | -1.13E-02 | 1.61E-02 | 0.483 | 0.42952 | G | A |
| rs12257077 | 4.11E-02 | 4.01E-02 | 0.305 | 0.0373931 | T | C |
| rs722988 | -3.97E-04 | 1.59E-02 | 0.980 | 0.360728 | C | T |
| rs34593439 | 1.55E-02 | 2.61E-02 | 0.553 | 0.0996784 | A | G |
| rs12927355 | -1.85E-02 | 1.65E-02 | 0.261 | 0.317627 | T | C |
| rs10275896 | 3.12E-02 | 1.78E-02 | 0.080 | 0.231236 | C | T |
| rs55993634 | 1.33E-02 | 2.85E-02 | 0.640 | 0.0884688 | G | C |
| rs35327136 | 1.71E-02 | 1.96E-02 | 0.383 | 0.177397 | A | C |
| rs113374757 | 6.44E-03 | 2.13E-02 | 0.762 | 0.161106 | T | C |
| rs855330 | -5.70E-03 | 1.81E-02 | 0.753 | 0.234201 | C | T |
| rs7130222 | -1.92E-02 | 1.63E-02 | 0.240 | 0.317475 | G | T |
| rs61839660 | -2.53E-02 | 2.62E-02 | 0.334 | 0.094231 | T | C |
| rs11203203 | -3.06E-02 | 1.60E-02 | 0.056 | 0.359225 | A | G |
| rs1350275 | -1.40E-02 | 1.68E-02 | 0.406 | 0.715488 | G | T |
| rs1701704 | -3.89E-02 | 1.61E-02 | 0.016 | 0.332362 | G | T |
| rs7237497 | 2.08E-02 | 2.07E-02 | 0.315 | 0.835786 | C | T |
| rs2303137 | -1.30E-02 | 1.53E-02 | 0.398 | 0.439857 | T | A |
| rs7776597 | -4.40E-02 | 3.71E-02 | 0.235 | 0.953715 | G | A |
| rs238873 | -5.41E-02 | 6.88E-02 | 0.432 | 0.0158894 | G | A |
| rs12644686 | 1.44E-02 | 2.12E-02 | 0.498 | 0.180271 | G | C |
| rs17106304 | 1.03E-02 | 1.62E-02 | 0.524 | 0.658687 | G | C |
| rs1050979 | 3.21E-02 | 1.53E-02 | 0.035 | 0.514642 | G | A |
| rs2111485 | -1.69E-02 | 1.57E-02 | 0.281 | 0.614578 | G | A |
| rs6434435 | 4.06E-02 | 2.08E-02 | 0.051 | 0.17047 | A | G |
| rs3087243 | 4.76E-03 | 1.54E-02 | 0.757 | 0.428363 | A | G |
| rs2188962 | 4.46E-03 | 1.54E-02 | 0.772 | 0.421487 | T | C |
| rs601338 | 8.28E-03 | 1.53E-02 | 0.588 | 0.479823 | A | G |
| rs231972 | 5.49E-03 | 2.47E-02 | 0.824 | 0.114006 | C | A |
| rs56994090 | 3.59E-03 | 1.64E-02 | 0.827 | 0.414764 | C | T |
| rs34536443 | 4.42E-02 | 3.95E-02 | 0.263 | 0.0426595 | C | G |
| rs202535 | -6.88E-03 | 2.07E-02 | 0.739 | 0.831095 | A | C |
| rs57209021 | 4.11E-02 | 1.83E-02 | 0.025 | 0.23198 | T | C |
| rs13259300 | -1.04E-02 | 1.59E-02 | 0.511 | 0.612164 | C | A |
| rs229527 | 1.31E-03 | 1.54E-02 | 0.932 | 0.415436 | A | C |
| rs28648882 | -1.20E-02 | 1.80E-02 | 0.506 | 0.22656 | A | G |
| rs663743 | 2.47E-02 | 1.62E-02 | 0.126 | 0.340502 | A | G |
| rs7068821 | 2.06E-02 | 1.74E-02 | 0.237 | 0.257009 | T | G |
| rs1794269 | -3.39E-02 | 1.59E-02 | 0.033 | 0.396139 | T | C |
| rs28752526 | -8.36E-03 | 1.63E-02 | 0.607 | 0.34849 | G | A |
| rs7752257 | -1.12E-02 | 1.77E-02 | 0.525 | 0.744894 | G | T |
| rs9260802 | 6.23E-02 | 4.63E-02 | 0.179 | 0.0318175 | G | A |
| rs2493411 | 6.83E-03 | 2.31E-02 | 0.767 | 0.128163 | C | T |
| rs7511678 | -9.61E-03 | 1.88E-02 | 0.610 | 0.209815 | A | G |
| rs17623914 | 2.60E-03 | 2.48E-02 | 0.917 | 0.105329 | C | T |
| rs1947178 | 3.37E-02 | 1.88E-02 | 0.073 | 0.782711 | G | A |
| rs10224046 | -1.93E-03 | 1.65E-02 | 0.907 | 0.312726 | G | T |
| rs78325861 | 2.25E-02 | 4.16E-02 | 0.589 | 0.0410946 | G | C |
| rs1808094 | 2.55E-02 | 1.52E-02 | 0.095 | 0.529853 | C | T |
| rs3802214 | -3.79E-03 | 1.95E-02 | 0.846 | 0.809194 | C | T |
| rs9385401 | 8.39E-03 | 1.52E-02 | 0.581 | 0.457527 | T | C |
| rs4820827 | 9.80E-03 | 1.56E-02 | 0.531 | 0.622926 | C | T |
| rs17323934 | 3.27E-02 | 1.82E-02 | 0.072 | 0.22252 | G | C |
| rs6908236 | 8.00E-03 | 1.52E-02 | 0.599 | 0.522308 | C | A |
| rs61759532 | -6.25E-03 | 1.97E-02 | 0.751 | 0.230615 | T | C |
| rs7936434 | -5.18E-04 | 1.54E-02 | 0.973 | 0.468587 | C | G |
| rs689 | 2.74E-02 | 1.71E-02 | 0.110 | 0.718917 | T | A |
| rs238265 | -1.77E-02 | 1.67E-02 | 0.288 | 0.697927 | G | T |
| rs2543537 | 3.50E-02 | 1.61E-02 | 0.029 | 0.434204 | T | C |
| rs3184504 | 9.83E-02 | 1.53E-02 | 0.000 | 0.519822 | C | T |
| rs1574285 | -5.81E-03 | 1.58E-02 | 0.713 | 0.585103 | T | G |
| rs12128789 | 1.81E-02 | 2.27E-02 | 0.426 | 0.132702 | C | T |
| rs10801128 | -5.88E-02 | 1.68E-02 | 0.000 | 0.711719 | G | A |
| rs8046043 | -2.14E-02 | 1.56E-02 | 0.169 | 0.402925 | C | G |
| rs2611211 | -3.00E-02 | 2.07E-02 | 0.147 | 0.8329 | T | C |
| rs4490209 | 5.99E-04 | 1.62E-02 | 0.970 | 0.348132 | G | C |
| rs1611236 | -3.27E-02 | 1.66E-02 | 0.049 | 0.319955 | A | G |
| rs3135348 | -1.92E-03 | 1.78E-02 | 0.914 | 0.5674 | G | A |
| rs4548024 | 8.37E-03 | 1.87E-02 | 0.654 | 0.228989 | C | T |
| rs6908626 | -1.06E-02 | 2.25E-02 | 0.638 | 0.163628 | T | G |
| rs114378220 | 1.99E-02 | 3.66E-02 | 0.587 | 0.0601701 | T | C |
| rs12464462 | 3.18E-02 | 1.55E-02 | 0.040 | 0.419363 | G | A |
| rs3024493 | -3.69E-02 | 2.10E-02 | 0.079 | 0.157635 | A | C |
| rs10751776 | -3.60E-03 | 1.53E-02 | 0.815 | 0.494916 | C | A |
| rs7795896 | 1.07E-02 | 1.65E-02 | 0.519 | 0.695163 | T | C |
| rs41295159 | 1.50E-02 | 7.63E-02 | 0.844 | 0.011258 | G | C |

**Supplementary Table 8**

Summary information of genetic instruments associated with esophageal cancer

| **SNP** | **Beta** | **SE** | **p-value** | **EAF** | **EA** | **NEA** |
| --- | --- | --- | --- | --- | --- | --- |
| rs4490209 | -0.0645 | 0.0304 | 0.034 | 0.4059 | G | C |
| rs7668577 | -0.0088 | 0.0487 | 0.857 | 0.3117 | C | A |
| rs1050979 | -0.0042 | 0.0304 | 0.889 | 0.4672 | G | A |
| rs7752257 | 0.0263 | 0.0358 | 0.463 | 0.7631 | G | T |
| rs2611211 | 0.0410 | 0.0333 | 0.218 | 0.7702 | T | C |
| rs9468618 | -0.0109 | 0.0593 | 0.854 | 0.0724 | T | C |
| rs73432769 | 0.1001 | 0.0659 | 0.129 | 0.0328 | T | C |
| rs6908236 | 0.0189 | 0.0294 | 0.521 | 0.5194 | C | A |
| rs238873 | 0.1238 | 0.1929 | 0.521 | 0.0143 | G | A |
| rs7068821 | -0.0166 | 0.0339 | 0.626 | 0.2494 | T | G |
| rs7301381 | 0.0207 | 0.0293 | 0.480 | 0.4707 | C | T |
| rs229527 | -0.0334 | 0.0304 | 0.271 | 0.4760 | A | C |
| rs3184504 | 0.0134 | 0.0452 | 0.767 | 0.6552 | C | T |
| rs34593439 | -0.0467 | 0.0551 | 0.397 | 0.0972 | A | G |
| rs35327136 | -0.0575 | 0.0604 | 0.341 | 0.1472 | A | C |
| rs11203203 | -0.0024 | 0.0425 | 0.956 | 0.2650 | A | G |
| rs113374757 | 0.0175 | 0.0384 | 0.649 | 0.1805 | T | C |
| rs601338 | 0.0093 | 0.0453 | 0.837 | 0.4720 | A | G |
| rs6679677 | -0.0062 | 0.0727 | 0.932 | 0.1152 | A | C |
| rs1881146 | -0.0065 | 0.0318 | 0.839 | 0.3354 | T | A |
| rs855330 | 0.0167 | 0.0342 | 0.625 | 0.2639 | C | T |
| rs3024493 | -0.0142 | 0.0585 | 0.808 | 0.1191 | A | C |
| rs2429557 | -0.1861 | 0.1602 | 0.246 | 0.0090 | A | T |
| rs3135348 | -1.0440 | 1.9812 | 0.598 | 0.0010 | T | A |
| rs10275896 | 0.0059 | 0.0409 | 0.885 | 0.1963 | C | T |
| rs4548024 | 0.0152 | 0.0407 | 0.709 | 0.2010 | C | T |
| rs1794269 | -0.0065 | 0.03 | 0.827 | 0.4248 | T | C |
| rs2303137 | -0.0391 | 0.0317 | 0.217 | 0.5199 | T | A |
| rs12257077 | 0.0615 | 0.1246 | 0.622 | 0.0307 | T | C |
| rs689 | -0.0120 | 0.0454 | 0.792 | 0.7913 | T | A |
| rs1574285 | -0.0133 | 0.0299 | 0.656 | 0.5829 | T | G |
| rs2111485 | 0.0102 | 0.0338 | 0.763 | 0.4954 | G | A |
| rs13147049 | 0.0034 | 0.0301 | 0.909 | 0.6007 | G | A |
| rs7936434 | 0.0663 | 0.0453 | 0.143 | 0.4604 | C | G |
| rs663743 | 0.0283 | 0.0337 | 0.401 | 0.3152 | A | G |
| rs8046043 | -0.0064 | 0.0317 | 0.839 | 0.3570 | C | G |
| rs1701704 | -0.0404 | 0.0342 | 0.238 | 0.2991 | G | T |
| rs17106304 | -0.0022 | 0.0314 | 0.945 | 0.6677 | G | C |
| rs55993634 | -0.0232 | 0.0408 | 0.569 | 0.1238 | G | C |
| rs9517712 | 0.0181 | 0.0373 | 0.628 | 0.7672 | C | T |
| rs57209021 | -0.0749 | 0.054 | 0.166 | 0.2242 | T | C |
| rs10801128 | -0.0630 | 0.031 | 0.042 | 0.6786 | G | A |
| rs17623914 | 0.1222 | 0.0744 | 0.101 | 0.0977 | C | T |
| rs28648882 | -0.0299 | 0.0572 | 0.602 | 0.2241 | A | G |
| rs3135348 | -0.0333 | 0.0462 | 0.470 | 0.5522 | G | A |
| rs9385401 | -0.0354 | 0.0468 | 0.450 | 0.6263 | T | C |
| rs28752526 | 0.0177 | 0.0316 | 0.575 | 0.3391 | G | A |
| rs1947178 | -0.0372 | 0.0388 | 0.338 | 0.8126 | G | A |
| rs7776597 | -0.0907 | 0.11 | 0.410 | 0.9599 | G | A |
| rs17323934 | 0.0042 | 0.0413 | 0.920 | 0.1900 | G | C |
| rs10224046 | 0.0116 | 0.0485 | 0.810 | 0.3296 | G | T |
| rs7795896 | 0.0651 | 0.0488 | 0.182 | 0.7670 | T | C |
| rs114378220 | 0.1058 | 0.0902 | 0.241 | 0.0693 | T | C |
| rs7130222 | 0.0747 | 0.0326 | 0.022 | 0.2940 | G | T |
| rs78325861 | 0.1229 | 0.1164 | 0.291 | 0.0384 | G | C |
| rs7237497 | 0.0441 | 0.0435 | 0.310 | 0.8535 | C | T |
| rs12927355 | 0.0059 | 0.0356 | 0.868 | 0.2767 | T | C |
| rs56994090 | -0.0129 | 0.0299 | 0.666 | 0.4283 | C | T |
| rs2543537 | 0.0509 | 0.0297 | 0.087 | 0.4572 | T | C |
| rs4820827 | 0.0046 | 0.0312 | 0.883 | 0.5440 | C | T |
| rs12464462 | -0.0356 | 0.0456 | 0.434 | 0.3065 | G | A |
| rs3087243 | -0.0262 | 0.0315 | 0.405 | 0.3796 | A | G |
| rs6434435 | -0.0054 | 0.0404 | 0.894 | 0.1551 | A | G |
| rs6908626 | -0.0459 | 0.0601 | 0.445 | 0.1610 | T | G |
| rs1611236 | 0.0075 | 0.0323 | 0.815 | 0.2902 | A | G |
| rs722988 | -0.0083 | 0.0315 | 0.791 | 0.4394 | C | T |
| rs238265 | 0.0366 | 0.0342 | 0.285 | 0.7165 | G | T |
| rs10844597 | -0.0364 | 0.0301 | 0.227 | 0.4676 | A | G |
| rs607703 | -0.0227 | 0.0296 | 0.443 | 0.4691 | T | C |
| rs1808094 | 0.0583 | 0.0302 | 0.054 | 0.5336 | C | T |
| rs34536443 | 0.0969 | 0.1127 | 0.390 | 0.0418 | C | G |
| rs61759532 | 0.0435 | 0.0465 | 0.349 | 0.1903 | T | C |
| rs1350275 | -0.0198 | 0.0307 | 0.521 | 0.6253 | G | T |
| rs12128789 | 0.0245 | 0.0661 | 0.711 | 0.1311 | C | T |
| rs7511678 | -0.0076 | 0.0318 | 0.810 | 0.2891 | A | G |
| rs55893453 | -0.0489 | 0.0495 | 0.323 | 0.1571 | G | A |
| rs2493411 | 0.1174 | 0.0557 | 0.035 | 0.1118 | C | T |
| rs10751776 | 0.0234 | 0.0297 | 0.431 | 0.5395 | C | A |
| rs12742756 | -0.0202 | 0.0304 | 0.507 | 0.4098 | G | A |
| rs574384 | 0.0789 | 0.0412 | 0.055 | 0.7197 | A | C |
| rs12644686 | -0.0292 | 0.0341 | 0.391 | 0.2880 | G | C |
| rs2188962 | -0.0320 | 0.0457 | 0.484 | 0.2974 | T | C |
| rs61839660 | -0.0109 | 0.0777 | 0.888 | 0.0826 | T | C |
| rs41295159 | -0.1810 | 0.2266 | 0.425 | 0.0085 | G | C |
| rs3802214 | -0.0004 | 0.0321 | 0.989 | 0.7277 | C | T |
| rs9260802 | 0.0262 | 0.1216 | 0.830 | 0.0358 | G | A |
| rs112733823 | -0.0096 | 0.0496 | 0.847 | 0.1298 | T | C |
| rs13259300 | 0.0040 | 0.0301 | 0.893 | 0.5569 | C | A |
| rs231972 | 0.0247 | 0.069 | 0.721 | 0.1154 | C | A |
| rs202535 | 0.0485 | 0.0609 | 0.425 | 0.8254 | A | C |

**Supplementary Table 9**

Summary information of genetic instruments associated with Gastric cancer

| **SNP** | **Beta** | **SE** | **p-value** | **EAF** | **EA** | **NEA** |
| --- | --- | --- | --- | --- | --- | --- |
| rs2111485 | -2.11E-02 | 0.0195 | 0.280 | 0.4921 | G | A |
| rs2493411 | 2.67E-02 | 0.0359 | 0.457 | 0.1110 | C | T |
| rs9385401 | -2.33E-02 | 0.047 | 0.621 | 0.6311 | T | C |
| rs238873 | -2.26E-02 | 0.1862 | 0.903 | 0.0143 | G | A |
| rs1881146 | 2.53E-02 | 0.0167 | 0.130 | 0.3360 | T | A |
| rs2611211 | -4.10E-03 | 0.0165 | 0.804 | 0.7685 | T | C |
| rs1574285 | 7.50E-03 | 0.016 | 0.638 | 0.5827 | T | G |
| rs41295159 | -7.52E-02 | 0.2509 | 0.764 | 0.0085 | G | C |
| rs78325861 | 2.98E-01 | 0.1178 | 0.011 | 0.0384 | G | C |
| rs56994090 | -3.29E-02 | 0.0161 | 0.040 | 0.4279 | C | T |
| rs8046043 | 4.00E-04 | 0.0175 | 0.981 | 0.3561 | C | G |
| rs55993634 | -2.61E-02 | 0.0197 | 0.185 | 0.1250 | G | C |
| rs10844597 | -1.73E-02 | 0.0163 | 0.290 | 0.4664 | A | G |
| rs229527 | -2.40E-03 | 0.0164 | 0.885 | 0.4779 | A | C |
| rs113374757 | -7.80E-03 | 0.0199 | 0.696 | 0.1809 | T | C |
| rs34593439 | -1.02E-02 | 0.0324 | 0.752 | 0.0968 | A | G |
| rs114378220 | 1.10E-01 | 0.0904 | 0.224 | 0.0693 | T | C |
| rs6908626 | -7.29E-02 | 0.0615 | 0.236 | 0.1610 | T | G |
| rs3135348 | 6.85E-01 | 1.3191 | 0.604 | 0.0010 | T | A |
| rs1050979 | 1.90E-03 | 0.0165 | 0.909 | 0.4658 | G | A |
| rs12464462 | -4.20E-02 | 0.0439 | 0.339 | 0.3033 | G | A |
| rs13147049 | -8.50E-03 | 0.0158 | 0.590 | 0.5997 | G | A |
| rs28752526 | -2.30E-03 | 0.0171 | 0.892 | 0.3386 | G | A |
| rs1350275 | -2.15E-02 | 0.0159 | 0.177 | 0.6232 | G | T |
| rs607703 | -7.00E-03 | 0.0159 | 0.659 | 0.4685 | T | C |
| rs238265 | -2.00E-04 | 0.0189 | 0.992 | 0.7172 | G | T |
| rs7936434 | 5.68E-02 | 0.0446 | 0.203 | 0.4603 | C | G |
| rs12644686 | -1.30E-02 | 0.0173 | 0.453 | 0.2907 | G | C |
| rs7668577 | -3.81E-02 | 0.0479 | 0.426 | 0.3117 | C | A |
| rs663743 | -1.47E-02 | 0.0189 | 0.438 | 0.3140 | A | G |
| rs35327136 | 3.37E-02 | 0.0644 | 0.601 | 0.1472 | A | C |
| rs7237497 | 3.43E-02 | 0.0246 | 0.164 | 0.8539 | C | T |
| rs10801128 | -2.84E-02 | 0.0162 | 0.080 | 0.6774 | G | A |
| rs2188962 | -3.08E-02 | 0.0453 | 0.496 | 0.2944 | T | C |
| rs3087243 | 1.40E-02 | 0.0174 | 0.423 | 0.3784 | A | G |
| rs4490209 | 2.10E-02 | 0.016 | 0.190 | 0.4073 | G | C |
| rs9260802 | -1.65E-01 | 0.1188 | 0.164 | 0.0358 | G | A |
| rs112733823 | 1.87E-02 | 0.028 | 0.504 | 0.1293 | T | C |
| rs13259300 | -7.80E-03 | 0.016 | 0.626 | 0.5557 | C | A |
| rs61839660 | -4.76E-02 | 0.0816 | 0.559 | 0.0826 | T | C |
| rs9517712 | 2.88E-02 | 0.0212 | 0.175 | 0.7680 | C | T |
| rs7795896 | 1.09E-02 | 0.046 | 0.813 | 0.7693 | T | C |
| rs57209021 | -4.44E-02 | 0.0536 | 0.408 | 0.2242 | T | C |
| rs2543537 | -1.37E-02 | 0.0159 | 0.390 | 0.4569 | T | C |
| rs202535 | -9.30E-03 | 0.0584 | 0.873 | 0.8254 | A | C |
| rs55893453 | -6.29E-02 | 0.0364 | 0.084 | 0.1558 | G | A |
| rs12128789 | -6.09E-02 | 0.0661 | 0.356 | 0.1311 | C | T |
| rs855330 | 1.14E-02 | 0.0182 | 0.531 | 0.2638 | C | T |
| rs6679677 | 2.00E-04 | 0.0694 | 0.997 | 0.1152 | A | C |
| rs10751776 | 5.70E-03 | 0.016 | 0.723 | 0.5403 | C | A |
| rs12742756 | 6.00E-04 | 0.0164 | 0.973 | 0.4091 | G | A |
| rs2303137 | -1.70E-03 | 0.0176 | 0.922 | 0.5222 | T | A |
| rs1794269 | -1.74E-02 | 0.016 | 0.276 | 0.4247 | T | C |
| rs6908236 | 5.70E-03 | 0.0157 | 0.715 | 0.5197 | C | A |
| rs73432769 | -1.09E-01 | 0.031 | 0.000 | 0.0332 | T | C |
| rs7068821 | 1.10E-02 | 0.0182 | 0.546 | 0.2494 | T | G |
| rs1947178 | -3.90E-03 | 0.0218 | 0.860 | 0.8131 | G | A |
| rs10275896 | 1.51E-02 | 0.0247 | 0.541 | 0.1953 | C | T |
| rs10224046 | -2.44E-02 | 0.0472 | 0.606 | 0.3296 | G | T |
| rs722988 | -1.30E-03 | 0.0171 | 0.941 | 0.4421 | C | T |
| rs17106304 | 2.09E-02 | 0.017 | 0.219 | 0.6681 | G | C |
| rs4548024 | 9.00E-04 | 0.0242 | 0.970 | 0.2001 | C | T |
| rs61759532 | -2.39E-02 | 0.0319 | 0.455 | 0.1890 | T | C |
| rs601338 | 3.29E-02 | 0.0449 | 0.463 | 0.4720 | A | G |
| rs1808094 | 2.35E-02 | 0.0161 | 0.146 | 0.5339 | C | T |
| rs7511678 | 5.19E-02 | 0.0161 | 0.001 | 0.2909 | A | G |
| rs6434435 | 2.00E-03 | 0.0219 | 0.926 | 0.1551 | A | G |
| rs574384 | -4.50E-03 | 0.0204 | 0.827 | 0.7143 | A | C |
| rs28648882 | 8.86E-02 | 0.0644 | 0.169 | 0.2241 | A | G |
| rs17623914 | 1.13E-01 | 0.0754 | 0.135 | 0.0977 | C | T |
| rs3024493 | 1.11E-02 | 0.0466 | 0.812 | 0.1181 | A | C |
| rs3135348 | -3.47E-02 | 0.047 | 0.460 | 0.5522 | G | A |
| rs7776597 | -4.77E-02 | 0.1138 | 0.675 | 0.9599 | G | A |
| rs17323934 | 1.60E-02 | 0.0249 | 0.521 | 0.1891 | G | C |
| rs3802214 | 3.06E-02 | 0.016 | 0.056 | 0.7258 | C | T |
| rs1611236 | -4.64E-02 | 0.0174 | 0.008 | 0.2900 | A | G |
| rs2429557 | -4.33E-02 | 0.0948 | 0.648 | 0.0090 | A | T |
| rs9468618 | 2.23E-02 | 0.0347 | 0.520 | 0.0722 | T | C |
| rs12257077 | -8.44E-02 | 0.1299 | 0.516 | 0.0307 | T | C |
| rs3184504 | -5.30E-02 | 0.0446 | 0.235 | 0.6587 | C | T |
| rs7130222 | 3.30E-03 | 0.0178 | 0.852 | 0.2936 | G | T |
| rs689 | 5.26E-02 | 0.0339 | 0.120 | 0.7931 | T | A |
| rs1701704 | -5.20E-03 | 0.0194 | 0.790 | 0.2980 | G | T |
| rs7301381 | 2.00E-02 | 0.0157 | 0.202 | 0.4709 | C | T |
| rs7752257 | 7.65E-02 | 0.0199 | 0.000 | 0.7638 | G | T |
| rs231972 | 1.99E-02 | 0.0697 | 0.775 | 0.1154 | C | A |
| rs12927355 | -2.71E-02 | 0.0206 | 0.187 | 0.2755 | T | C |
| rs4820827 | -6.30E-03 | 0.017 | 0.709 | 0.5415 | C | T |
| rs34536443 | 7.00E-02 | 0.1145 | 0.541 | 0.0418 | C | G |
| rs11203203 | -1.46E-02 | 0.0318 | 0.645 | 0.2626 | A | G |

**Supplementary Table 10**

Summary information of genetic instruments associated with head and neck cancer

| **SNP** | **Beta** | **SE** | **p-value** | **EAF** | **EA** | **NEA** |
| --- | --- | --- | --- | --- | --- | --- |
| rs6434435 | -1.69E-04 | 0.0002 | 0.320 | 0.1754 | A | G |
| rs2493411 | -1.96E-04 | 0.0002 | 0.300 | 0.1248 | C | T |
| rs574384 | -5.65E-05 | 0.0002 | 0.790 | 0.9045 | A | C |
| rs12742756 | -3.24E-05 | 0.0001 | 0.800 | 0.4271 | G | A |
| rs663743 | 1.83E-04 | 0.0001 | 0.170 | 0.3399 | A | G |
| rs6908626 | 1.18E-05 | 0.0002 | 0.940 | 0.1788 | T | G |
| rs1611236 | 2.07E-04 | 0.0001 | 0.120 | 0.3238 | A | G |
| rs112733823 | -1.31E-04 | 0.0002 | 0.510 | 0.1157 | T | C |
| rs3135348 | 6.66E-06 | 0.0001 | 0.960 | 0.5597 | G | A |
| rs7752257 | 2.74E-04 | 0.0001 | 0.058 | 0.7424 | G | T |
| rs607703 | -1.31E-05 | 0.0001 | 0.920 | 0.4731 | T | C |
| rs113374757 | -3.43E-04 | 0.0002 | 0.045 | 0.1637 | T | C |
| rs8046043 | 1.14E-04 | 0.0001 | 0.370 | 0.4160 | C | G |
| rs56994090 | 9.64E-05 | 0.0001 | 0.450 | 0.4151 | C | T |
| rs229527 | -1.91E-04 | 0.0001 | 0.130 | 0.4239 | A | C |
| rs1947178 | -9.53E-05 | 0.0002 | 0.530 | 0.7744 | G | A |
| rs4548024 | 1.40E-04 | 0.0001 | 0.350 | 0.2372 | C | T |
| rs4490209 | -2.95E-04 | 0.0001 | 0.024 | 0.3621 | G | C |
| rs10751776 | -1.77E-05 | 0.0001 | 0.890 | 0.5009 | C | A |
| rs6679677 | 6.42E-06 | 0.0002 | 0.980 | 0.1011 | A | C |
| rs2111485 | 6.25E-05 | 0.0001 | 0.630 | 0.6076 | G | A |
| rs12644686 | -1.84E-04 | 0.0002 | 0.270 | 0.1792 | G | C |
| rs1881146 | 1.27E-04 | 0.0001 | 0.350 | 0.3126 | T | A |
| rs7936434 | -4.16E-05 | 0.0001 | 0.740 | 0.4775 | C | G |
| rs3184504 | 1.14E-04 | 0.0001 | 0.360 | 0.5164 | C | T |
| rs12927355 | -1.13E-04 | 0.0001 | 0.400 | 0.3225 | T | C |
| rs1350275 | 2.39E-05 | 0.0001 | 0.870 | 0.7217 | G | T |
| rs55993634 | -5.64E-05 | 0.0002 | 0.810 | 0.0826 | G | C |
| rs11203203 | 4.40E-06 | 0.0001 | 0.970 | 0.3675 | A | G |
| rs689 | 2.81E-04 | 0.0001 | 0.044 | 0.7110 | T | A |
| rs3802214 | 1.58E-04 | 0.0002 | 0.330 | 0.8185 | C | T |
| rs7068821 | -2.19E-04 | 0.0001 | 0.130 | 0.2570 | T | G |
| rs114378220 | 2.34E-05 | 0.0003 | 0.930 | 0.0737 | T | C |
| rs2429557 | -5.50E-04 | 0.0006 | 0.340 | 0.0122 | A | T |
| rs238873 | -3.15E-04 | 0.0005 | 0.560 | 0.0136 | G | A |
| rs6908236 | 4.31E-06 | 0.0001 | 0.970 | 0.5302 | C | A |
| rs2303137 | 1.48E-04 | 0.0001 | 0.240 | 0.4331 | T | A |
| rs7668577 | 9.46E-05 | 0.0001 | 0.490 | 0.3109 | C | A |
| rs17623914 | 1.42E-04 | 0.0002 | 0.490 | 0.1086 | C | T |
| rs7511678 | 1.14E-04 | 0.0002 | 0.470 | 0.2023 | A | G |
| rs3024493 | 9.30E-05 | 0.0002 | 0.590 | 0.1541 | A | C |
| rs7776597 | 4.91E-05 | 0.0003 | 0.870 | 0.9550 | G | A |
| rs722988 | -3.09E-04 | 0.0001 | 0.018 | 0.3665 | C | T |
| rs34593439 | 4.39E-05 | 0.0002 | 0.830 | 0.1039 | A | G |
| rs231972 | -1.45E-04 | 0.0002 | 0.450 | 0.1253 | C | A |
| rs1808094 | -5.65E-05 | 0.0001 | 0.650 | 0.5248 | C | T |
| rs601338 | 1.09E-04 | 0.0001 | 0.390 | 0.5080 | A | G |
| rs34536443 | -3.39E-05 | 0.0003 | 0.910 | 0.0451 | C | G |
| rs4820827 | 1.04E-06 | 0.0001 | 0.990 | 0.6059 | C | T |
| rs13259300 | -1.48E-04 | 0.0001 | 0.260 | 0.6101 | C | A |
| rs7301381 | 2.55E-05 | 0.0001 | 0.840 | 0.4641 | C | T |
| rs7795896 | -6.86E-05 | 0.0001 | 0.620 | 0.7058 | T | C |
| rs10275896 | 2.15E-05 | 0.0001 | 0.880 | 0.2448 | C | T |
| rs78325861 | -2.52E-04 | 0.0003 | 0.430 | 0.0406 | G | C |
| rs3087243 | -9.03E-05 | 0.0001 | 0.480 | 0.4498 | A | G |
| rs1701704 | -2.03E-04 | 0.0001 | 0.130 | 0.3422 | G | T |
| rs10801128 | 5.25E-05 | 0.0001 | 0.700 | 0.7101 | G | A |
| rs12128789 | -1.10E-05 | 0.0002 | 0.950 | 0.1371 | C | T |
| rs12464462 | -1.70E-04 | 0.0001 | 0.180 | 0.4143 | G | A |
| rs2611211 | -1.74E-04 | 0.0002 | 0.320 | 0.8427 | T | C |
| rs1050979 | 1.00E-05 | 0.0001 | 0.940 | 0.5267 | G | A |
| rs2188962 | 9.78E-05 | 0.0001 | 0.440 | 0.4269 | T | C |
| rs9260802 | -5.56E-05 | 0.0003 | 0.870 | 0.0354 | G | A |
| rs855330 | -7.51E-05 | 0.0001 | 0.620 | 0.2323 | C | T |
| rs1574285 | -1.60E-04 | 0.0001 | 0.210 | 0.5992 | T | G |
| rs61839660 | -1.07E-04 | 0.0002 | 0.610 | 0.0979 | T | C |
| rs17106304 | -3.71E-05 | 0.0001 | 0.780 | 0.6562 | G | C |
| rs35327136 | -6.06E-05 | 0.0002 | 0.710 | 0.1853 | A | C |
| rs7237497 | -1.64E-04 | 0.0002 | 0.330 | 0.8350 | C | T |
| rs55893453 | -1.03E-04 | 0.0002 | 0.500 | 0.2139 | G | A |
| rs28648882 | -1.02E-04 | 0.0002 | 0.500 | 0.2253 | A | G |
| rs9468618 | -2.63E-04 | 0.0002 | 0.230 | 0.0895 | T | C |
| rs13147049 | 1.69E-04 | 0.0001 | 0.210 | 0.6714 | G | A |
| rs12257077 | 6.94E-04 | 0.0003 | 0.038 | 0.0367 | T | C |
| rs9517712 | 8.15E-06 | 0.0001 | 0.960 | 0.7495 | C | T |
| rs61759532 | -6.72E-06 | 0.0002 | 0.960 | 0.2465 | T | C |
| rs57209021 | 7.36E-05 | 0.0002 | 0.620 | 0.2310 | T | C |
| rs7130222 | -1.68E-05 | 0.0001 | 0.900 | 0.3255 | G | T |
| rs10224046 | 7.21E-05 | 0.0001 | 0.600 | 0.3099 | G | T |
| rs238265 | -3.93E-05 | 0.0001 | 0.780 | 0.7022 | G | T |
| rs202535 | 1.48E-04 | 0.0002 | 0.390 | 0.8401 | A | C |
| rs73432769 | -1.38E-06 | 0.0004 | 1.000 | 0.0210 | T | C |
| rs9385401 | -3.02E-04 | 0.0001 | 0.017 | 0.4560 | T | C |
| rs17323934 | 5.96E-05 | 0.0001 | 0.690 | 0.2415 | G | C |
| rs41295159 | -3.26E-04 | 0.0006 | 0.590 | 0.0124 | G | C |
| rs2543537 | 1.31E-04 | 0.0001 | 0.310 | 0.4354 | T | C |

**Supplementary Table 11**

Summary information of genetic instruments associated with leukaemia

| **SNP** | **Beta** | **SE** | **p-value** | **EAF** | **EA** | **NEA** |
| --- | --- | --- | --- | --- | --- | --- |
| rs6679677 | -1.16E-04 | 0.0002 | 0.600 | 0.1010 | A | C |
| rs12464462 | -4.14E-05 | 0.0001 | 0.760 | 0.4143 | G | A |
| rs6434435 | -6.50E-05 | 0.0002 | 0.720 | 0.1754 | A | G |
| rs12742756 | -9.04E-05 | 0.0001 | 0.510 | 0.4271 | G | A |
| rs7668577 | 4.41E-04 | 0.0001 | 0.002 | 0.3110 | C | A |
| rs10751776 | -1.13E-04 | 0.0001 | 0.400 | 0.5008 | C | A |
| rs1574285 | 5.96E-05 | 0.0001 | 0.660 | 0.5992 | T | G |
| rs78325861 | 6.34E-04 | 0.0003 | 0.062 | 0.0406 | G | C |
| rs9517712 | 3.68E-05 | 0.0002 | 0.810 | 0.7495 | C | T |
| rs202535 | 4.76E-05 | 0.0002 | 0.800 | 0.8400 | A | C |
| rs34536443 | -1.55E-04 | 0.0003 | 0.640 | 0.0451 | C | G |
| rs10224046 | 1.00E-04 | 0.0001 | 0.490 | 0.3099 | G | T |
| rs7130222 | 1.45E-04 | 0.0001 | 0.310 | 0.3256 | G | T |
| rs10801128 | -1.60E-04 | 0.0001 | 0.280 | 0.7101 | G | A |
| rs3024493 | 3.03E-04 | 0.0002 | 0.100 | 0.1541 | A | C |
| rs1881146 | -1.06E-04 | 0.0001 | 0.470 | 0.3126 | T | A |
| rs574384 | 3.26E-05 | 0.0002 | 0.890 | 0.9045 | A | C |
| rs2493411 | -4.53E-06 | 0.0002 | 0.980 | 0.1248 | C | T |
| rs1808094 | 3.99E-05 | 0.0001 | 0.770 | 0.5248 | C | T |
| rs607703 | -1.06E-05 | 0.0001 | 0.940 | 0.4731 | T | C |
| rs11203203 | -1.52E-04 | 0.0001 | 0.270 | 0.3675 | A | G |
| rs7301381 | 1.07E-04 | 0.0001 | 0.430 | 0.4641 | C | T |
| rs1701704 | -1.29E-04 | 0.0001 | 0.360 | 0.3422 | G | T |
| rs10275896 | -1.63E-04 | 0.0002 | 0.300 | 0.2447 | C | T |
| rs17106304 | -1.91E-04 | 0.0001 | 0.180 | 0.6561 | G | C |
| rs1350275 | 1.82E-04 | 0.0002 | 0.230 | 0.7218 | G | T |
| rs7936434 | -3.60E-04 | 0.0001 | 0.007 | 0.4775 | C | G |
| rs722988 | -6.11E-05 | 0.0001 | 0.660 | 0.3666 | C | T |
| rs238873 | -1.08E-04 | 0.0006 | 0.850 | 0.0136 | G | A |
| rs73432769 | -3.17E-04 | 0.0005 | 0.500 | 0.0210 | T | C |
| rs6908236 | 3.19E-05 | 0.0001 | 0.810 | 0.5302 | C | A |
| rs4548024 | -1.43E-04 | 0.0002 | 0.370 | 0.2371 | C | T |
| rs13259300 | -5.01E-05 | 0.0001 | 0.720 | 0.6101 | C | A |
| rs2188962 | 3.31E-04 | 0.0001 | 0.015 | 0.4270 | T | C |
| rs28648882 | -4.10E-04 | 0.0002 | 0.011 | 0.2253 | A | G |
| rs1611236 | -1.51E-04 | 0.0001 | 0.290 | 0.3237 | A | G |
| rs9260802 | 2.49E-04 | 0.0004 | 0.490 | 0.0354 | G | A |
| rs7511678 | 2.62E-04 | 0.0002 | 0.120 | 0.2023 | A | G |
| rs12644686 | 1.15E-05 | 0.0002 | 0.950 | 0.1793 | G | C |
| rs13147049 | 1.55E-04 | 0.0001 | 0.280 | 0.6714 | G | A |
| rs2111485 | -3.00E-05 | 0.0001 | 0.830 | 0.6076 | G | A |
| rs1947178 | 1.53E-04 | 0.0002 | 0.340 | 0.7745 | G | A |
| rs7237497 | 1.82E-04 | 0.0002 | 0.320 | 0.8350 | C | T |
| rs4820827 | 1.37E-05 | 0.0001 | 0.920 | 0.6059 | C | T |
| rs663743 | 1.20E-04 | 0.0001 | 0.400 | 0.3399 | A | G |
| rs57209021 | 3.70E-04 | 0.0002 | 0.021 | 0.2311 | T | C |
| rs3802214 | 6.55E-05 | 0.0002 | 0.710 | 0.8185 | C | T |
| rs7776597 | -3.18E-04 | 0.0003 | 0.330 | 0.9550 | G | A |
| rs7068821 | 1.96E-04 | 0.0002 | 0.200 | 0.2571 | T | G |
| rs2303137 | 1.00E-04 | 0.0001 | 0.460 | 0.4330 | T | A |
| rs12257077 | 2.96E-04 | 0.0004 | 0.410 | 0.0366 | T | C |
| rs41295159 | 2.37E-04 | 0.0007 | 0.720 | 0.0109 | G | C |
| rs855330 | -5.94E-05 | 0.0002 | 0.710 | 0.2323 | C | T |
| rs12128789 | 8.78E-05 | 0.0002 | 0.650 | 0.1371 | C | T |
| rs4490209 | -2.27E-05 | 0.0001 | 0.870 | 0.3622 | G | C |
| rs2611211 | -1.38E-05 | 0.0002 | 0.940 | 0.8427 | T | C |
| rs9468618 | -9.62E-06 | 0.0002 | 0.970 | 0.0895 | T | C |
| rs7752257 | -4.92E-05 | 0.0002 | 0.750 | 0.7423 | G | T |
| rs1050979 | 3.89E-04 | 0.0001 | 0.004 | 0.5268 | G | A |
| rs6908626 | 3.70E-06 | 0.0002 | 0.980 | 0.1788 | T | G |
| rs7795896 | -8.35E-05 | 0.0001 | 0.570 | 0.7058 | T | C |
| rs61839660 | 6.41E-05 | 0.0002 | 0.780 | 0.0979 | T | C |
| rs689 | -9.54E-05 | 0.0001 | 0.520 | 0.7109 | T | A |
| rs56994090 | 1.43E-04 | 0.0001 | 0.290 | 0.4152 | C | T |
| rs34593439 | -1.88E-04 | 0.0002 | 0.390 | 0.1038 | A | G |
| rs61759532 | -1.43E-04 | 0.0002 | 0.370 | 0.2465 | T | C |
| rs601338 | -1.00E-04 | 0.0001 | 0.450 | 0.5080 | A | G |
| rs17623914 | -4.96E-04 | 0.0002 | 0.023 | 0.1086 | C | T |
| rs114378220 | 1.71E-04 | 0.0003 | 0.520 | 0.0737 | T | C |
| rs55893453 | -9.23E-05 | 0.0002 | 0.570 | 0.2139 | G | A |
| rs3087243 | 8.74E-05 | 0.0001 | 0.520 | 0.4498 | A | G |
| rs112733823 | 5.68E-04 | 0.0002 | 0.007 | 0.1158 | T | C |
| rs2429557 | -5.92E-05 | 0.0006 | 0.920 | 0.0122 | A | T |
| rs3135348 | 3.55E-04 | 0.0001 | 0.009 | 0.5598 | G | A |
| rs55993634 | -1.97E-06 | 0.0002 | 0.990 | 0.0826 | G | C |
| rs8046043 | -1.08E-04 | 0.0001 | 0.430 | 0.4160 | C | G |
| rs12927355 | 3.25E-04 | 0.0001 | 0.024 | 0.3226 | T | C |
| rs238265 | 1.93E-04 | 0.0001 | 0.190 | 0.7023 | G | T |
| rs231972 | -2.63E-05 | 0.0002 | 0.900 | 0.1253 | C | A |
| rs3184504 | 1.95E-05 | 0.0001 | 0.880 | 0.5164 | C | T |
| rs113374757 | -3.06E-04 | 0.0002 | 0.094 | 0.1637 | T | C |
| rs229527 | 2.80E-04 | 0.0001 | 0.040 | 0.4241 | A | C |
| rs35327136 | 2.77E-05 | 0.0002 | 0.870 | 0.1853 | A | C |
| rs9385401 | -2.97E-04 | 0.0001 | 0.027 | 0.4560 | T | C |
| rs17323934 | -2.03E-04 | 0.0002 | 0.200 | 0.2414 | G | C |
| rs2543537 | 2.41E-04 | 0.0001 | 0.078 | 0.4354 | T | C |

**Supplementary Table 12**

Summary information of genetic instruments associated with liver cancer

| **SNP** | **Beta** | **SE** | **p-value** | **EAF** | **EA** | **NA** |
| --- | --- | --- | --- | --- | --- | --- |
| rs2111485 | 0.0615 | 0.0355 | 0.083 | 0.4949 | G | A |
| rs10751776 | -0.0269 | 0.0293 | 0.360 | 0.5396 | C | A |
| rs55893453 | -0.0442 | 0.0646 | 0.494 | 0.1569 | G | A |
| rs2303137 | -0.0041 | 0.0321 | 0.899 | 0.5203 | T | A |
| rs73432769 | -0.0400 | 0.0575 | 0.487 | 0.0329 | T | C |
| rs6908236 | 0.0291 | 0.0288 | 0.313 | 0.5194 | C | A |
| rs4820827 | 0.0349 | 0.0310 | 0.261 | 0.5437 | C | T |
| rs663743 | -0.0607 | 0.0345 | 0.079 | 0.3150 | A | G |
| rs231972 | 0.0711 | 0.1163 | 0.541 | 0.1154 | C | A |
| rs13259300 | 0.0383 | 0.0293 | 0.191 | 0.5568 | C | A |
| rs238265 | 0.0415 | 0.0346 | 0.230 | 0.7166 | G | T |
| rs3802214 | 0.0132 | 0.0295 | 0.653 | 0.7274 | C | T |
| rs2543537 | 0.0277 | 0.0291 | 0.341 | 0.4571 | T | C |
| rs6434435 | 0.0869 | 0.0402 | 0.031 | 0.1551 | A | G |
| rs3087243 | 0.0067 | 0.0319 | 0.833 | 0.3794 | A | G |
| rs7511678 | -0.0158 | 0.0295 | 0.592 | 0.2894 | A | G |
| rs1881146 | -0.0339 | 0.0311 | 0.275 | 0.3354 | T | A |
| rs9260802 | 0.1009 | 0.1944 | 0.604 | 0.0358 | G | A |
| rs9468618 | -0.1641 | 0.0633 | 0.010 | 0.0723 | T | C |
| rs7752257 | 0.1044 | 0.0365 | 0.004 | 0.7633 | G | T |
| rs7795896 | 0.0263 | 0.0759 | 0.729 | 0.7673 | T | C |
| rs10275896 | -0.0166 | 0.0449 | 0.711 | 0.1961 | C | T |
| rs112733823 | -0.0408 | 0.0505 | 0.419 | 0.1297 | T | C |
| rs114378220 | 0.2683 | 0.1499 | 0.074 | 0.0692 | T | C |
| rs7068821 | 0.0046 | 0.0334 | 0.890 | 0.2494 | T | G |
| rs78325861 | 0.0562 | 0.1952 | 0.773 | 0.0384 | G | C |
| rs1350275 | 0.0068 | 0.0292 | 0.817 | 0.6250 | G | T |
| rs601338 | 0.0028 | 0.0742 | 0.970 | 0.4719 | A | G |
| rs57209021 | 0.0501 | 0.0884 | 0.571 | 0.2242 | T | C |
| rs7237497 | 0.0092 | 0.0447 | 0.837 | 0.8535 | C | T |
| rs229527 | 0.0048 | 0.0301 | 0.874 | 0.4763 | A | C |
| rs574384 | 0.0170 | 0.0375 | 0.651 | 0.7189 | A | C |
| rs12464462 | -0.0828 | 0.0728 | 0.256 | 0.3060 | G | A |
| rs17623914 | -0.2813 | 0.1272 | 0.027 | 0.0977 | C | T |
| rs238873 | -0.0404 | 0.2992 | 0.893 | 0.0143 | G | A |
| rs28752526 | -0.0259 | 0.0314 | 0.409 | 0.3390 | G | A |
| rs17323934 | -0.0083 | 0.0451 | 0.855 | 0.1898 | G | C |
| rs7776597 | 0.0852 | 0.1931 | 0.659 | 0.9600 | G | A |
| rs12644686 | -0.0274 | 0.0318 | 0.388 | 0.2884 | G | C |
| rs689 | -0.0362 | 0.0595 | 0.543 | 0.7915 | T | A |
| rs11203203 | 0.0262 | 0.0559 | 0.640 | 0.2646 | A | G |
| rs202535 | -0.0632 | 0.0945 | 0.504 | 0.8254 | A | C |
| rs17106304 | -0.0128 | 0.0311 | 0.680 | 0.6677 | G | C |
| rs7301381 | 0.0018 | 0.0287 | 0.951 | 0.4707 | C | T |
| rs1808094 | -0.0079 | 0.0299 | 0.791 | 0.5336 | C | T |
| rs34536443 | -0.5186 | 0.1900 | 0.006 | 0.0418 | C | G |
| rs12742756 | -0.0069 | 0.0301 | 0.819 | 0.4097 | G | A |
| rs6679677 | 0.1045 | 0.1119 | 0.350 | 0.1152 | A | C |
| rs10801128 | 0.0085 | 0.0298 | 0.774 | 0.6785 | G | A |
| rs12128789 | 0.1091 | 0.1096 | 0.319 | 0.1311 | C | T |
| rs3024493 | -0.0374 | 0.0802 | 0.641 | 0.1190 | A | C |
| rs1794269 | -0.0231 | 0.0292 | 0.430 | 0.4249 | T | C |
| rs6908626 | -0.1105 | 0.1029 | 0.283 | 0.1610 | T | G |
| rs1050979 | -0.0252 | 0.0302 | 0.403 | 0.4670 | G | A |
| rs2188962 | -0.1231 | 0.0753 | 0.102 | 0.2969 | T | C |
| rs10224046 | -0.0321 | 0.0772 | 0.678 | 0.3296 | G | T |
| rs9385401 | -0.0493 | 0.0841 | 0.558 | 0.6271 | T | C |
| rs4548024 | 0.0012 | 0.0441 | 0.979 | 0.2009 | C | T |
| rs1947178 | -0.0283 | 0.0399 | 0.478 | 0.8127 | G | A |
| rs722988 | 0.0138 | 0.0313 | 0.661 | 0.4398 | C | T |
| rs12257077 | -0.2007 | 0.2225 | 0.367 | 0.0307 | T | C |
| rs35327136 | -0.0902 | 0.1119 | 0.421 | 0.1472 | A | C |
| rs113374757 | 0.0233 | 0.0369 | 0.528 | 0.1805 | T | C |
| rs855330 | 0.0085 | 0.0332 | 0.798 | 0.2639 | C | T |
| rs2493411 | -0.0631 | 0.0636 | 0.322 | 0.1117 | C | T |
| rs4490209 | -0.0132 | 0.0297 | 0.656 | 0.4062 | G | C |
| rs3135348 | 2.4717 | 1.8739 | 0.187 | 0.0010 | T | A |
| rs3135348 | -0.0510 | 0.0787 | 0.517 | 0.5522 | G | A |
| rs1611236 | 0.0432 | 0.0320 | 0.177 | 0.2902 | A | G |
| rs2429557 | 0.1747 | 0.1687 | 0.300 | 0.0090 | A | T |
| rs28648882 | -0.1612 | 0.1144 | 0.159 | 0.2240 | A | G |
| rs13147049 | 0.0155 | 0.0290 | 0.592 | 0.6006 | G | A |
| rs7668577 | -0.0730 | 0.0788 | 0.354 | 0.3117 | C | A |
| rs2611211 | 0.0115 | 0.0304 | 0.705 | 0.7699 | T | C |
| rs1574285 | -0.0255 | 0.0292 | 0.383 | 0.5828 | T | G |
| rs10844597 | 0.0109 | 0.0299 | 0.715 | 0.4675 | A | G |
| rs61839660 | -0.2464 | 0.1396 | 0.077 | 0.0826 | T | C |
| rs41295159 | -0.0014 | 0.4415 | 0.998 | 0.0085 | G | C |
| rs9517712 | 0.0060 | 0.0387 | 0.877 | 0.7673 | C | T |
| rs56994090 | -0.0755 | 0.0294 | 0.010 | 0.4282 | C | T |
| rs1701704 | -0.0319 | 0.0354 | 0.368 | 0.2989 | G | T |
| rs7936434 | 0.0776 | 0.0734 | 0.291 | 0.4603 | C | G |
| rs7130222 | -0.0199 | 0.0326 | 0.541 | 0.2938 | G | T |
| rs3184504 | 0.1605 | 0.0735 | 0.029 | 0.6558 | C | T |
| rs607703 | 0.0822 | 0.0291 | 0.005 | 0.4691 | T | C |
| rs34593439 | 0.0138 | 0.0585 | 0.814 | 0.0971 | A | G |
| rs12927355 | 0.0748 | 0.0373 | 0.045 | 0.2766 | T | C |
| rs61759532 | -0.0087 | 0.0568 | 0.878 | 0.1901 | T | C |
| rs55993634 | -0.0288 | 0.0364 | 0.428 | 0.1240 | G | C |
| rs8046043 | -0.0121 | 0.0320 | 0.705 | 0.3568 | C | G |

**Supplementary Table 13**

Summary information of genetic instruments associated with lung cancer

| **SNP** | **Beta** | **SE** | **p-value** | **EAF** | **EA** | **NEA** |
| --- | --- | --- | --- | --- | --- | --- |
| rs1881146 | -0.0191 | 0.0131 | 0.144 | 0.3169 | T | A |
| rs12464462 | -0.0259 | 0.0120 | 0.031 | 0.4018 | G | A |
| rs34536443 | 0.1337 | 0.0309 | 0.000 | 0.0393 | C | G |
| rs2611211 | 0.0259 | 0.0157 | 0.098 | 0.8458 | T | C |
| rs7776597 | 0.0072 | 0.0297 | 0.807 | 0.9505 | G | A |
| rs3135348 | 0.0405 | 0.0119 | 0.001 | 0.5606 | G | A |
| rs73432769 | -0.0493 | 0.0439 | 0.261 | 0.0212 | T | C |
| rs2303137 | 0.0039 | 0.0118 | 0.742 | 0.4633 | T | A |
| rs4820827 | 0.0203 | 0.0121 | 0.094 | 0.6305 | C | T |
| rs7068821 | -0.0146 | 0.0136 | 0.285 | 0.2517 | T | G |
| rs6679677 | -0.0418 | 0.0201 | 0.037 | 0.1053 | A | C |
| rs3024493 | -0.0074 | 0.0163 | 0.649 | 0.1441 | A | C |
| rs12128789 | -0.0199 | 0.0179 | 0.266 | 0.1223 | C | T |
| rs1794269 | 0.0654 | 0.0125 | 0.000 | 0.3573 | T | C |
| rs7752257 | 0.0194 | 0.0141 | 0.170 | 0.7346 | G | T |
| rs1574285 | -0.0037 | 0.0120 | 0.759 | 0.5879 | T | G |
| rs12257077 | 0.0143 | 0.0302 | 0.636 | 0.0385 | T | C |
| rs10275896 | 0.0239 | 0.0137 | 0.081 | 0.2414 | C | T |
| rs6908236 | 0.0127 | 0.0117 | 0.276 | 0.5147 | C | A |
| rs9517712 | 0.0149 | 0.0137 | 0.278 | 0.7607 | C | T |
| rs12644686 | -0.0083 | 0.0160 | 0.604 | 0.1856 | G | C |
| rs56994090 | -0.0057 | 0.0126 | 0.651 | 0.4149 | C | T |
| rs3802214 | -0.0138 | 0.0150 | 0.358 | 0.8095 | C | T |
| rs7130222 | -0.0022 | 0.0127 | 0.863 | 0.3143 | G | T |
| rs11203203 | -0.0039 | 0.0121 | 0.746 | 0.3767 | A | G |
| rs17623914 | 0.0079 | 0.0191 | 0.679 | 0.1064 | C | T |
| rs12742756 | 0.0073 | 0.0124 | 0.557 | 0.4194 | G | A |
| rs28648882 | -0.0055 | 0.0137 | 0.687 | 0.2280 | A | G |
| rs7511678 | 0.0149 | 0.0143 | 0.295 | 0.2054 | A | G |
| rs10224046 | 0.0079 | 0.0126 | 0.531 | 0.3133 | G | T |
| rs34593439 | -0.0821 | 0.0206 | 0.000 | 0.1028 | A | G |
| rs722988 | 0.0197 | 0.0120 | 0.100 | 0.3659 | C | T |
| rs229527 | -0.0211 | 0.0118 | 0.074 | 0.4143 | A | C |
| rs3184504 | 0.0345 | 0.0119 | 0.004 | 0.5485 | C | T |
| rs17106304 | -0.0036 | 0.0125 | 0.772 | 0.6633 | G | C |
| rs35327136 | -0.0202 | 0.0156 | 0.194 | 0.1712 | A | C |
| rs8046043 | -0.0072 | 0.0121 | 0.550 | 0.4037 | C | G |
| rs7795896 | 0.0015 | 0.0126 | 0.907 | 0.6941 | T | C |
| rs114378220 | -0.0284 | 0.0279 | 0.308 | 0.0699 | T | C |
| rs6908626 | -0.0175 | 0.0170 | 0.302 | 0.1886 | T | G |
| rs9468618 | -0.0367 | 0.0210 | 0.080 | 0.0876 | T | C |
| rs1050979 | 0.0318 | 0.0117 | 0.006 | 0.4808 | G | A |
| rs13259300 | -0.0289 | 0.0124 | 0.020 | 0.6099 | C | A |
| rs689 | -0.0013 | 0.0134 | 0.925 | 0.7286 | T | A |
| rs574384 | -0.0087 | 0.0197 | 0.658 | 0.9008 | A | C |
| rs4490209 | 0.0213 | 0.0121 | 0.078 | 0.3644 | G | C |
| rs55893453 | -0.0109 | 0.0147 | 0.457 | 0.1980 | G | A |
| rs2111485 | -0.0358 | 0.0120 | 0.003 | 0.6065 | G | A |
| rs13147049 | 0.0118 | 0.0125 | 0.345 | 0.6777 | G | A |
| rs7668577 | -0.0102 | 0.0137 | 0.457 | 0.2996 | C | A |
| rs7936434 | 0.0264 | 0.0119 | 0.027 | 0.4565 | C | G |
| rs4548024 | -0.0160 | 0.0147 | 0.276 | 0.2009 | C | T |
| rs2493411 | 0.0033 | 0.0177 | 0.853 | 0.1276 | C | T |
| rs10751776 | 0.0244 | 0.0117 | 0.037 | 0.4965 | C | A |
| rs238265 | -0.0083 | 0.0128 | 0.516 | 0.6950 | G | T |
| rs231972 | -0.0073 | 0.0188 | 0.699 | 0.1208 | C | A |
| rs2543537 | -0.0196 | 0.0122 | 0.106 | 0.4402 | T | C |
| rs855330 | -0.0094 | 0.0139 | 0.498 | 0.2315 | C | T |
| rs9260802 | -0.0091 | 0.0325 | 0.780 | 0.0368 | G | A |
| rs238873 | 0.0390 | 0.0548 | 0.476 | 0.0150 | G | A |
| rs112733823 | -0.0407 | 0.0219 | 0.064 | 0.1016 | T | C |
| rs9385401 | -0.0023 | 0.0117 | 0.845 | 0.4557 | T | C |
| rs6434435 | -0.0023 | 0.0153 | 0.879 | 0.1883 | A | G |
| rs3087243 | -0.0086 | 0.0119 | 0.469 | 0.4296 | A | G |
| rs2188962 | 0.0116 | 0.0119 | 0.331 | 0.4066 | T | C |
| rs17323934 | 0.0276 | 0.0140 | 0.048 | 0.2362 | G | C |
| rs78325861 | 0.0592 | 0.0308 | 0.055 | 0.0461 | G | C |
| rs607703 | -0.0223 | 0.0117 | 0.057 | 0.4835 | T | C |
| rs663743 | 0.0112 | 0.0126 | 0.374 | 0.3304 | A | G |
| rs7237497 | 0.0211 | 0.0163 | 0.194 | 0.8328 | C | T |
| rs10844597 | 0.0076 | 0.0117 | 0.517 | 0.5005 | A | G |
| rs7301381 | 0.0411 | 0.0118 | 0.000 | 0.4412 | C | T |
| rs1701704 | -0.0413 | 0.0124 | 0.001 | 0.3247 | G | T |
| rs1947178 | -0.0109 | 0.0139 | 0.430 | 0.7728 | G | A |
| rs1350275 | 0.0141 | 0.0129 | 0.275 | 0.7170 | G | T |
| rs601338 | 0.0266 | 0.0118 | 0.024 | 0.5109 | A | G |
| rs1808094 | 0.0201 | 0.0117 | 0.087 | 0.5151 | C | T |
| rs113374757 | 0.0002 | 0.0162 | 0.988 | 0.1544 | T | C |
| rs57209021 | 0.0232 | 0.0142 | 0.101 | 0.2269 | T | C |
| rs202535 | 0.0007 | 0.0153 | 0.962 | 0.8302 | A | C |
| rs28752526 | -0.0112 | 0.0136 | 0.410 | 0.3185 | G | A |
| rs1611236 | 0.0725 | 0.0130 | 0.000 | 0.2996 | A | G |
| rs41295159 | -0.0359 | 0.0590 | 0.543 | 0.0112 | G | C |

**Supplementary Table 14**

Summary information of genetic instruments associated with lymphoma

| **SNP** | **Beta** | **SE** | **p-value** | **EAF** | **EA** | **NEA** |
| --- | --- | --- | --- | --- | --- | --- |
| rs3024493 | 8.11E-05 | 2.26E-04 | 0.720 | 0.1541 | A | C |
| rs6434435 | -2.97E-04 | 2.18E-04 | 0.173 | 0.1748 | A | G |
| rs114378220 | 9.71E-05 | 3.25E-04 | 0.765 | 0.0738 | T | C |
| rs28752526 | -6.26E-04 | 1.71E-04 | 0.000 | 0.3664 | G | A |
| rs34593439 | 1.78E-04 | 2.68E-04 | 0.507 | 0.1043 | A | G |
| rs7068821 | -1.61E-04 | 1.87E-04 | 0.389 | 0.2587 | T | G |
| rs55893453 | 3.80E-04 | 2.00E-04 | 0.057 | 0.2143 | G | A |
| rs17323934 | 1.26E-04 | 1.93E-04 | 0.515 | 0.2345 | G | C |
| rs113374757 | 8.56E-05 | 2.22E-04 | 0.700 | 0.1641 | T | C |
| rs7301381 | 4.12E-05 | 1.64E-04 | 0.802 | 0.4656 | C | T |
| rs238265 | 9.06E-05 | 1.79E-04 | 0.613 | 0.7011 | G | T |
| rs61839660 | -7.55E-05 | 2.74E-04 | 0.783 | 0.0993 | T | C |
| rs2303137 | 2.89E-05 | 1.65E-04 | 0.861 | 0.4332 | T | A |
| rs3135348 | 7.59E-05 | 1.65E-04 | 0.645 | 0.5571 | G | A |
| rs73432769 | 4.09E-05 | 5.74E-04 | 0.943 | 0.0207 | T | C |
| rs7752257 | 3.35E-04 | 1.87E-04 | 0.074 | 0.7405 | G | T |
| rs1611236 | 2.02E-04 | 1.75E-04 | 0.249 | 0.3226 | A | G |
| rs9260802 | -2.58E-04 | 4.45E-04 | 0.562 | 0.0351 | G | A |
| rs6679677 | 1.93E-05 | 2.69E-04 | 0.943 | 0.1025 | A | C |
| rs12742756 | 1.87E-05 | 1.65E-04 | 0.910 | 0.4256 | G | A |
| rs2611211 | 4.04E-04 | 2.25E-04 | 0.073 | 0.8435 | T | C |
| rs13147049 | 9.20E-05 | 1.74E-04 | 0.597 | 0.6693 | G | A |
| rs689 | -2.64E-04 | 1.81E-04 | 0.145 | 0.7100 | T | A |
| rs10224046 | -8.81E-05 | 1.77E-04 | 0.619 | 0.3101 | G | T |
| rs35327136 | -4.08E-04 | 2.11E-04 | 0.053 | 0.1852 | A | C |
| rs12927355 | 5.27E-04 | 1.75E-04 | 0.003 | 0.3208 | T | C |
| rs10844597 | 2.99E-05 | 1.63E-04 | 0.855 | 0.4956 | A | G |
| rs41295159 | 1.22E-03 | 7.87E-04 | 0.120 | 0.0112 | G | C |
| rs1808094 | -3.45E-05 | 1.64E-04 | 0.833 | 0.5253 | C | T |
| rs56994090 | 2.21E-04 | 1.66E-04 | 0.183 | 0.4154 | C | T |
| rs202535 | 2.58E-04 | 2.24E-04 | 0.249 | 0.8417 | A | C |
| rs4820827 | -9.19E-05 | 1.67E-04 | 0.583 | 0.6046 | C | T |
| rs1881146 | 1.02E-04 | 1.77E-04 | 0.566 | 0.3129 | T | A |
| rs12464462 | -1.57E-04 | 1.66E-04 | 0.343 | 0.4176 | G | A |
| rs10801128 | 9.15E-05 | 1.80E-04 | 0.612 | 0.7099 | G | A |
| rs17623914 | -4.28E-04 | 2.66E-04 | 0.108 | 0.1085 | C | T |
| rs57209021 | -3.33E-04 | 1.96E-04 | 0.089 | 0.2300 | T | C |
| rs7130222 | 2.43E-04 | 1.75E-04 | 0.165 | 0.3251 | G | T |
| rs3184504 | 1.92E-04 | 1.64E-04 | 0.240 | 0.5180 | C | T |
| rs78325861 | 4.54E-04 | 4.14E-04 | 0.273 | 0.0405 | G | C |
| rs13259300 | 8.65E-05 | 1.69E-04 | 0.609 | 0.6065 | C | A |
| rs9385401 | -1.07E-04 | 1.64E-04 | 0.516 | 0.4539 | T | C |
| rs229527 | -3.01E-04 | 1.65E-04 | 0.069 | 0.4223 | A | C |
| rs34536443 | 4.03E-04 | 4.01E-04 | 0.315 | 0.0461 | C | G |
| rs10275896 | 1.49E-04 | 1.90E-04 | 0.435 | 0.2451 | C | T |
| rs2188962 | 2.28E-04 | 1.65E-04 | 0.168 | 0.4293 | T | C |
| rs6908236 | 2.35E-05 | 1.64E-04 | 0.886 | 0.5331 | C | A |
| rs663743 | 2.06E-05 | 1.73E-04 | 0.905 | 0.3417 | A | G |
| rs9517712 | 3.13E-04 | 1.89E-04 | 0.097 | 0.7495 | C | T |
| rs7936434 | 5.33E-05 | 1.64E-04 | 0.745 | 0.4776 | C | G |
| rs601338 | 7.19E-05 | 1.64E-04 | 0.661 | 0.5104 | A | G |
| rs2429557 | -2.55E-04 | 7.47E-04 | 0.733 | 0.0121 | A | T |
| rs112733823 | -3.18E-05 | 2.54E-04 | 0.900 | 0.1172 | T | C |
| rs4490209 | 1.80E-04 | 1.70E-04 | 0.290 | 0.3605 | G | C |
| rs2493411 | -4.76E-04 | 2.48E-04 | 0.055 | 0.1243 | C | T |
| rs3087243 | 1.26E-04 | 1.64E-04 | 0.445 | 0.4501 | A | G |
| rs28648882 | -9.68E-05 | 1.96E-04 | 0.622 | 0.2240 | A | G |
| rs1701704 | -3.65E-04 | 1.72E-04 | 0.034 | 0.3436 | G | T |
| rs855330 | -1.60E-04 | 1.94E-04 | 0.409 | 0.2319 | C | T |
| rs6908626 | -1.18E-04 | 2.14E-04 | 0.583 | 0.1798 | T | G |
| rs7668577 | -7.63E-07 | 1.76E-04 | 0.997 | 0.3123 | C | A |
| rs9468618 | -1.69E-04 | 2.86E-04 | 0.556 | 0.0895 | T | C |
| rs1350275 | 4.13E-04 | 1.83E-04 | 0.024 | 0.7229 | G | T |
| rs17106304 | -9.06E-07 | 1.72E-04 | 0.996 | 0.6553 | G | C |
| rs607703 | -4.82E-04 | 1.64E-04 | 0.003 | 0.4738 | T | C |
| rs231972 | -6.01E-04 | 2.48E-04 | 0.015 | 0.1247 | C | A |
| rs55993634 | 1.82E-04 | 3.02E-04 | 0.545 | 0.0827 | G | C |
| rs574384 | 7.64E-05 | 2.83E-04 | 0.787 | 0.9072 | A | C |
| rs12644686 | 1.00E-04 | 2.15E-04 | 0.641 | 0.1781 | G | C |
| rs1050979 | 6.54E-05 | 1.64E-04 | 0.690 | 0.5298 | G | A |
| rs722988 | 1.12E-04 | 1.71E-04 | 0.510 | 0.3647 | C | T |
| rs10751776 | 1.68E-04 | 1.64E-04 | 0.304 | 0.4993 | C | A |
| rs7511678 | -3.56E-04 | 2.04E-04 | 0.081 | 0.2017 | A | G |
| rs12128789 | -1.75E-04 | 2.38E-04 | 0.461 | 0.1380 | C | T |
| rs2111485 | -7.68E-06 | 1.67E-04 | 0.963 | 0.6096 | G | A |
| rs1947178 | 2.03E-04 | 1.98E-04 | 0.304 | 0.7778 | G | A |
| rs12257077 | -3.34E-05 | 4.41E-04 | 0.940 | 0.0357 | T | C |
| rs238873 | 2.66E-04 | 7.07E-04 | 0.706 | 0.0136 | G | A |
| rs1794269 | 1.07E-03 | 1.69E-04 | 0.000 | 0.3955 | T | C |
| rs4548024 | -4.35E-05 | 1.94E-04 | 0.822 | 0.2378 | C | T |
| rs61759532 | -3.78E-04 | 1.95E-04 | 0.053 | 0.2485 | T | C |
| rs8046043 | -1.24E-04 | 1.66E-04 | 0.455 | 0.4176 | C | G |
| rs7795896 | -4.89E-04 | 1.79E-04 | 0.006 | 0.7043 | T | C |
| rs1574285 | -1.93E-05 | 1.67E-04 | 0.908 | 0.6015 | T | G |
| rs3802214 | -3.26E-04 | 2.12E-04 | 0.125 | 0.8184 | C | T |
| rs7776597 | 1.77E-04 | 3.92E-04 | 0.652 | 0.9540 | G | A |
| rs2543537 | -5.36E-04 | 1.66E-04 | 0.001 | 0.4461 | T | C |
| rs11203203 | 1.03E-04 | 1.70E-04 | 0.545 | 0.3657 | A | G |
| rs7237497 | -1.61E-04 | 2.20E-04 | 0.465 | 0.8340 | C | T |

**Supplementary Table 15**

Summary information of genetic instruments associated with malignant melanoma

| **SNP** | **Beta** | **SE** | **p-value** | **EAF** | **EA** | **NEA** |
| --- | --- | --- | --- | --- | --- | --- |
| rs17623914 | 2.79E-04 | 2.96E-04 | 0.35 | 0.1089 | C | T |
| rs12464462 | 2.28E-04 | 1.86E-04 | 0.22 | 0.4142 | G | A |
| rs3802214 | 6.04E-06 | 2.37E-04 | 0.98 | 0.8187 | C | T |
| rs8046043 | 1.64E-06 | 1.86E-04 | 0.99 | 0.4158 | C | G |
| rs10224046 | 9.60E-05 | 1.98E-04 | 0.63 | 0.3101 | G | T |
| rs2188962 | -8.60E-05 | 1.85E-04 | 0.64 | 0.4277 | T | C |
| rs9517712 | 1.22E-04 | 2.11E-04 | 0.56 | 0.7491 | C | T |
| rs1701704 | -1.62E-04 | 1.92E-04 | 0.4 | 0.3413 | G | T |
| rs1050979 | 3.53E-04 | 1.82E-04 | 0.053 | 0.5283 | G | A |
| rs7795896 | 2.75E-04 | 2.01E-04 | 0.17 | 0.7052 | T | C |
| rs113374757 | 1.70E-04 | 2.48E-04 | 0.49 | 0.1637 | T | C |
| rs601338 | -2.14E-04 | 1.82E-04 | 0.24 | 0.5078 | A | G |
| rs202535 | 6.10E-04 | 2.49E-04 | 0.014 | 0.8402 | A | C |
| rs6908626 | -6.50E-04 | 2.40E-04 | 0.007 | 0.1777 | T | G |
| rs12128789 | -2.01E-04 | 2.66E-04 | 0.45 | 0.1369 | C | T |
| rs1881146 | 3.06E-05 | 1.98E-04 | 0.88 | 0.3126 | T | A |
| rs61839660 | 4.42E-04 | 3.07E-04 | 0.15 | 0.0982 | T | C |
| rs722988 | 2.09E-04 | 1.90E-04 | 0.27 | 0.3667 | C | T |
| rs13259300 | -4.55E-05 | 1.89E-04 | 0.81 | 0.6103 | C | A |
| rs238265 | -3.45E-04 | 2.00E-04 | 0.085 | 0.7023 | G | T |
| rs7301381 | 2.43E-04 | 1.83E-04 | 0.18 | 0.4652 | C | T |
| rs10844597 | 2.39E-05 | 1.82E-04 | 0.9 | 0.4969 | A | G |
| rs689 | 1.95E-04 | 2.03E-04 | 0.34 | 0.7108 | T | A |
| rs17323934 | -2.50E-04 | 2.16E-04 | 0.25 | 0.2343 | G | C |
| rs9385401 | -2.77E-04 | 1.83E-04 | 0.13 | 0.4558 | T | C |
| rs34593439 | 8.95E-04 | 2.99E-04 | 0.003 | 0.1042 | A | G |
| rs7237497 | 6.13E-04 | 2.47E-04 | 0.013 | 0.8354 | C | T |
| rs7668577 | 6.30E-08 | 1.97E-04 | 1 | 0.311 | C | A |
| rs55893453 | -4.86E-04 | 2.23E-04 | 0.029 | 0.2132 | G | A |
| rs12742756 | 7.77E-05 | 1.85E-04 | 0.67 | 0.427 | G | A |
| rs855330 | 2.16E-04 | 2.17E-04 | 0.32 | 0.2322 | C | T |
| rs2493411 | -4.35E-04 | 2.76E-04 | 0.12 | 0.1249 | C | T |
| rs10751776 | -2.74E-04 | 1.83E-04 | 0.13 | 0.5004 | C | A |
| rs6434435 | 2.45E-04 | 2.44E-04 | 0.31 | 0.1755 | A | G |
| rs2303137 | 2.05E-04 | 1.84E-04 | 0.27 | 0.4335 | T | A |
| rs6908236 | -2.86E-04 | 1.83E-04 | 0.12 | 0.5304 | C | A |
| rs229527 | 2.22E-04 | 1.85E-04 | 0.23 | 0.4234 | A | C |
| rs7511678 | 2.32E-05 | 2.28E-04 | 0.92 | 0.2022 | A | G |
| rs28648882 | -2.35E-04 | 2.19E-04 | 0.28 | 0.2249 | A | G |
| rs4820827 | 2.18E-04 | 1.87E-04 | 0.24 | 0.6061 | C | T |
| rs3184504 | 1.71E-04 | 1.83E-04 | 0.35 | 0.5173 | C | T |
| rs7130222 | 2.34E-05 | 1.95E-04 | 0.9 | 0.3253 | G | T |
| rs2543537 | 2.00E-04 | 1.86E-04 | 0.28 | 0.444 | T | C |
| rs12644686 | -1.95E-04 | 2.40E-04 | 0.42 | 0.1791 | G | C |
| rs13147049 | 3.39E-05 | 1.94E-04 | 0.86 | 0.6714 | G | A |
| rs10275896 | -2.24E-04 | 2.12E-04 | 0.29 | 0.2448 | C | T |
| rs1947178 | 3.05E-04 | 2.19E-04 | 0.16 | 0.7745 | G | A |
| rs4490209 | -1.99E-05 | 1.90E-04 | 0.92 | 0.3623 | G | C |
| rs2111485 | -1.10E-04 | 1.87E-04 | 0.56 | 0.6067 | G | A |
| rs56994090 | 7.21E-05 | 1.85E-04 | 0.7 | 0.415 | C | T |
| rs61759532 | -3.22E-04 | 2.18E-04 | 0.14 | 0.2463 | T | C |
| rs57209021 | -3.19E-05 | 2.18E-04 | 0.88 | 0.2311 | T | C |
| rs7936434 | -1.83E-04 | 1.83E-04 | 0.32 | 0.4781 | C | G |
| rs1350275 | 6.05E-04 | 2.04E-04 | 0.003 | 0.722 | G | T |
| rs17106304 | -1.25E-04 | 1.92E-04 | 0.52 | 0.6562 | G | C |
| rs2611211 | -9.20E-05 | 2.51E-04 | 0.71 | 0.8426 | T | C |
| rs12927355 | 1.93E-04 | 1.95E-04 | 0.32 | 0.3226 | T | C |
| rs10801128 | -1.34E-04 | 2.01E-04 | 0.5 | 0.71 | G | A |
| rs1574285 | -2.87E-04 | 1.87E-04 | 0.12 | 0.5988 | T | G |
| rs6679677 | -3.86E-04 | 3.03E-04 | 0.2 | 0.1008 | A | C |
| rs3087243 | 2.17E-04 | 1.83E-04 | 0.24 | 0.4507 | A | G |
| rs3135348 | -4.62E-04 | 1.84E-04 | 0.012 | 0.5593 | G | A |
| rs231972 | 1.64E-04 | 2.76E-04 | 0.55 | 0.1254 | C | A |
| rs11203203 | -1.74E-04 | 1.89E-04 | 0.36 | 0.3674 | A | G |
| rs7068821 | 2.14E-04 | 2.09E-04 | 0.31 | 0.2574 | T | G |
| rs663743 | 3.06E-04 | 1.93E-04 | 0.11 | 0.3405 | A | G |
| rs607703 | 1.99E-05 | 1.83E-04 | 0.91 | 0.4728 | T | C |
| rs35327136 | -3.71E-04 | 2.36E-04 | 0.11 | 0.1851 | A | C |
| rs3024493 | -2.08E-04 | 2.53E-04 | 0.41 | 0.1541 | A | C |
| rs7752257 | 1.80E-04 | 2.12E-04 | 0.39 | 0.7514 | G | T |
| rs28752526 | -3.29E-04 | 1.90E-04 | 0.084 | 0.3583 | G | A |
| rs1611236 | 1.54E-05 | 1.95E-04 | 0.94 | 0.3244 | A | G |
| rs112733823 | -7.59E-05 | 2.86E-04 | 0.79 | 0.1153 | T | C |
| rs1808094 | 2.84E-04 | 1.83E-04 | 0.12 | 0.5246 | C | T |

**Supplementary Table 16**

Summary information of genetic instruments associated with malignant neoplasm of kidney

| **SNP** | **Beta** | **SE** | **p-value** | **EAF** | **EA** | **NEA** |
| --- | --- | --- | --- | --- | --- | --- |
| rs601338 | -1.70E-05 | 0.0001 | 0.870 | 0.5078 | A | G |
| rs7301381 | 6.65E-05 | 0.0001 | 0.520 | 0.4652 | C | T |
| rs7130222 | 7.10E-05 | 0.0001 | 0.510 | 0.3253 | G | T |
| rs3184504 | 5.64E-05 | 0.0001 | 0.580 | 0.5173 | C | T |
| rs12464462 | 1.00E-04 | 0.0001 | 0.330 | 0.4142 | G | A |
| rs4820827 | -2.35E-05 | 0.0001 | 0.820 | 0.6061 | C | T |
| rs2303137 | 1.14E-04 | 0.0001 | 0.270 | 0.4335 | T | A |
| rs9385401 | 6.87E-05 | 0.0001 | 0.500 | 0.4558 | T | C |
| rs1050979 | -7.49E-05 | 0.0001 | 0.460 | 0.5283 | G | A |
| rs2188962 | -1.89E-04 | 0.0001 | 0.067 | 0.4277 | T | C |
| rs17106304 | 7.79E-05 | 0.0001 | 0.470 | 0.6562 | G | C |
| rs10751776 | 3.72E-05 | 0.0001 | 0.710 | 0.5004 | C | A |
| rs4490209 | -1.36E-04 | 0.0001 | 0.200 | 0.3623 | G | C |
| rs1701704 | -1.27E-04 | 0.0001 | 0.240 | 0.3413 | G | T |
| rs663743 | -1.22E-04 | 0.0001 | 0.260 | 0.3405 | A | G |
| rs7936434 | 1.43E-05 | 0.0001 | 0.890 | 0.4781 | C | G |
| rs10844597 | -1.59E-04 | 0.0001 | 0.120 | 0.4969 | A | G |
| rs1574285 | -2.39E-04 | 0.0001 | 0.021 | 0.5988 | T | G |
| rs3087243 | -5.92E-05 | 0.0001 | 0.560 | 0.4507 | A | G |
| rs3135348 | 2.78E-05 | 0.0001 | 0.790 | 0.5593 | G | A |
| rs13259300 | 7.88E-05 | 0.0001 | 0.450 | 0.6103 | C | A |
| rs56994090 | -1.54E-04 | 0.0001 | 0.140 | 0.4150 | C | T |
| rs13147049 | 1.49E-04 | 0.0001 | 0.170 | 0.6714 | G | A |
| rs229527 | -2.28E-04 | 0.0001 | 0.027 | 0.4234 | A | C |
| rs11203203 | 1.20E-04 | 0.0001 | 0.250 | 0.3674 | A | G |
| rs8046043 | 5.05E-05 | 0.0001 | 0.630 | 0.4158 | C | G |
| rs2111485 | -3.29E-04 | 0.0001 | 0.002 | 0.6067 | G | A |
| rs607703 | 1.39E-04 | 0.0001 | 0.170 | 0.4728 | T | C |
| rs2543537 | 9.38E-05 | 0.0001 | 0.360 | 0.4440 | T | C |
| rs12742756 | 1.23E-04 | 0.0001 | 0.230 | 0.4270 | G | A |
| rs722988 | -1.22E-04 | 0.0001 | 0.250 | 0.3667 | C | T |
| rs12927355 | 2.49E-04 | 0.0001 | 0.022 | 0.3226 | T | C |
| rs6908236 | 2.82E-05 | 0.0001 | 0.780 | 0.5304 | C | A |
| rs28752526 | 3.81E-05 | 0.0001 | 0.720 | 0.3583 | G | A |
| rs1611236 | 3.13E-05 | 0.0001 | 0.770 | 0.3244 | A | G |
| rs7795896 | 5.52E-05 | 0.0001 | 0.610 | 0.3161 | C | T |
| rs10224046 | 2.38E-05 | 0.0001 | 0.830 | 0.3159 | G | T |
| rs689 | -6.88E-05 | 0.0001 | 0.530 | 0.6856 | T | A |
| rs1808094 | -2.13E-05 | 0.0001 | 0.830 | 0.5246 | C | T |

**Supplementary Table 17**

Summary information of genetic instruments associated with multiple myeloma

| **SNP** | **Beta** | **SE** | **p-value** | **EAF** | **EA** | **NEA** |
| --- | --- | --- | --- | --- | --- | --- |
| rs9296062 | -6.46E-05 | 2.23E-04 | 0.770 | 0.0456 | C | G |
| rs10774624 | 2.39E-05 | 9.40E-05 | 0.800 | 0.5127 | A | G |
| rs9468618 | -2.58E-04 | 1.63E-04 | 0.110 | 0.0895 | T | C |
| rs12722495 | 8.12E-05 | 1.49E-04 | 0.590 | 0.1091 | C | T |
| rs689 | 7.25E-05 | 1.03E-04 | 0.480 | 0.7109 | T | A |
| rs1869449 | -1.37E-05 | 1.02E-04 | 0.890 | 0.2987 | A | G |
| rs34536443 | -1.38E-04 | 2.31E-04 | 0.550 | 0.0451 | C | G |
| rs741172 | 1.38E-05 | 9.97E-05 | 0.890 | 0.3209 | T | C |
| rs194749 | -1.22E-04 | 1.09E-04 | 0.260 | 0.2421 | C | T |
| rs4566101 | -8.20E-05 | 1.05E-04 | 0.440 | 0.2673 | C | T |
| rs6719660 | 1.15E-04 | 2.03E-04 | 0.570 | 0.9428 | G | A |
| rs10760335 | 1.19E-04 | 9.95E-05 | 0.230 | 0.3232 | G | A |
| rs6909461 | -6.18E-05 | 1.11E-04 | 0.580 | 0.2275 | C | A |
| rs2144013 | -1.33E-04 | 1.15E-04 | 0.250 | 0.2054 | G | A |
| rs17125653 | 4.08E-04 | 1.72E-04 | 0.018 | 0.0797 | A | T |
| rs55996894 | -3.57E-05 | 1.22E-04 | 0.770 | 0.1787 | C | G |
| rs1131017 | 1.36E-05 | 9.40E-05 | 0.890 | 0.5711 | G | C |
| rs79075295 | -5.11E-04 | 2.67E-04 | 0.056 | 0.0726 | A | G |
| rs9273363 | 1.44E-05 | 1.02E-04 | 0.890 | 0.3005 | A | C |
| rs6679677 | 3.23E-05 | 1.54E-04 | 0.830 | 0.1011 | A | C |
| rs34954 | -5.80E-05 | 3.47E-04 | 0.870 | 0.0327 | A | C |
| rs2269247 | -8.96E-05 | 1.21E-04 | 0.460 | 0.1819 | T | C |
| rs8056814 | 4.10E-04 | 1.68E-04 | 0.015 | 0.0840 | A | G |
| rs13182737 | 2.61E-05 | 1.06E-04 | 0.810 | 0.2593 | A | G |
| rs10830227 | 4.70E-05 | 9.41E-05 | 0.620 | 0.5760 | A | G |
| rs34954 | 1.61E-04 | 1.66E-04 | 0.330 | 0.0856 | G | C |
| rs11571297 | -3.17E-05 | 9.36E-05 | 0.730 | 0.4884 | C | T |
| rs202520 | 2.98E-05 | 1.04E-04 | 0.770 | 0.7199 | G | A |
| rs10911399 | -6.31E-05 | 2.75E-04 | 0.820 | 0.0294 | G | A |
| rs17863786 | -7.70E-04 | 2.97E-04 | 0.009 | 0.0259 | G | A |
| rs62410259 | 3.16E-04 | 1.78E-04 | 0.075 | 0.0756 | A | G |
| rs506770 | -3.08E-04 | 1.09E-04 | 0.005 | 0.7641 | C | G |
| rs185774696 | 5.35E-06 | 1.19E-04 | 0.960 | 0.2966 | T | C |
| rs231971 | -2.40E-05 | 1.71E-04 | 0.890 | 0.0806 | G | A |
| rs201417739 | 3.83E-05 | 3.12E-04 | 0.900 | 0.0546 | C | A |
| rs2071647 | 1.76E-04 | 1.05E-04 | 0.092 | 0.2716 | A | T |
| rs77523242 | -1.30E-04 | 2.02E-04 | 0.520 | 0.0590 | C | T |
| rs10183097 | 2.41E-05 | 1.35E-04 | 0.860 | 0.1397 | C | T |
| rs10865468 | 5.33E-05 | 1.08E-04 | 0.620 | 0.2541 | C | G |
| rs2111485 | -5.86E-05 | 9.52E-05 | 0.540 | 0.6076 | G | A |

**Supplementary Table 18**

Summary information of genetic instruments associated with ovarian cancer

| **SNP** | **Beta** | **SE** | **p-value** | **EAF** | **EA** | **NEA** |
| --- | --- | --- | --- | --- | --- | --- |
| rs3024493 | 1.11E-03 | 1.81E-02 | 0.951 | 0.162 | A | C |
| rs6679677 | 3.71E-02 | 2.25E-02 | 0.099 | 0.086 | A | C |
| rs7668577 | 1.60E-02 | 1.44E-02 | 0.267 | 0.323 | C | A |
| rs12742756 | -4.65E-03 | 1.42E-02 | 0.743 | 0.423 | G | A |
| rs12128789 | 5.34E-03 | 1.97E-02 | 0.787 | 0.134 | C | T |
| rs4490209 | -1.55E-02 | 1.39E-02 | 0.268 | 0.337 | G | C |
| rs2303137 | -4.97E-03 | 1.34E-02 | 0.710 | 0.439 | T | A |
| rs28752526 | 4.19E-03 | 1.40E-02 | 0.765 | 0.317 | G | A |
| rs17323934 | 8.83E-03 | 1.58E-02 | 0.577 | 0.220 | G | C |
| rs6908236 | 3.84E-03 | 1.32E-02 | 0.772 | 0.512 | C | A |
| rs238873 | -9.22E-02 | 6.09E-02 | 0.130 | 0.012 | G | A |
| rs12644686 | 3.42E-02 | 1.84E-02 | 0.063 | 0.173 | G | C |
| rs2188962 | 9.30E-03 | 1.34E-02 | 0.487 | 0.424 | T | C |
| rs7795896 | 3.41E-02 | 1.44E-02 | 0.018 | 0.693 | T | C |
| rs12257077 | 2.24E-02 | 3.35E-02 | 0.504 | 0.043 | T | C |
| rs1808094 | 3.26E-03 | 1.32E-02 | 0.805 | 0.526 | C | T |
| rs34593439 | -4.27E-02 | 2.23E-02 | 0.056 | 0.109 | A | G |
| rs55993634 | 2.90E-02 | 2.42E-02 | 0.232 | 0.096 | G | C |
| rs855330 | 1.71E-02 | 1.59E-02 | 0.282 | 0.233 | C | T |
| rs7776597 | 7.25E-03 | 3.29E-02 | 0.826 | 0.951 | G | A |
| rs4548024 | 1.66E-04 | 1.65E-02 | 0.992 | 0.216 | C | T |
| rs73432769 | -1.73E-02 | 4.52E-02 | 0.701 | 0.025 | T | C |
| rs7068821 | 4.49E-03 | 1.52E-02 | 0.767 | 0.258 | T | G |
| rs689 | -2.14E-03 | 1.47E-02 | 0.884 | 0.724 | T | A |
| rs61839660 | 3.57E-03 | 2.30E-02 | 0.877 | 0.096 | T | C |
| rs12927355 | 1.24E-02 | 1.42E-02 | 0.382 | 0.308 | T | C |
| rs231972 | 3.40E-04 | 2.12E-02 | 0.987 | 0.116 | C | A |
| rs229527 | -2.26E-02 | 1.34E-02 | 0.092 | 0.417 | A | C |
| rs3802214 | -2.37E-02 | 1.72E-02 | 0.167 | 0.826 | C | T |
| rs57209021 | -6.18E-04 | 1.59E-02 | 0.969 | 0.235 | T | C |
| rs238265 | -2.76E-02 | 1.46E-02 | 0.058 | 0.697 | G | T |
| rs574384 | 1.58E-02 | 2.21E-02 | 0.475 | 0.905 | A | C |
| rs2429557 | -5.72E-02 | 6.49E-02 | 0.378 | 0.009 | A | T |
| rs7752257 | 1.84E-02 | 1.54E-02 | 0.231 | 0.756 | G | T |
| rs6434435 | 3.48E-02 | 1.82E-02 | 0.057 | 0.167 | A | G |
| rs13259300 | 1.14E-02 | 1.39E-02 | 0.411 | 0.618 | C | A |
| rs6908626 | -2.46E-02 | 1.99E-02 | 0.215 | 0.169 | T | G |
| rs9385401 | -1.87E-02 | 1.32E-02 | 0.157 | 0.455 | T | C |
| rs2493411 | -4.07E-03 | 1.99E-02 | 0.838 | 0.127 | C | T |
| rs12464462 | 3.16E-03 | 1.35E-02 | 0.815 | 0.419 | G | A |
| rs114378220 | -9.92E-03 | 3.22E-02 | 0.758 | 0.066 | T | C |
| rs35327136 | 1.20E-01 | 1.70E-02 | 0.000 | 0.181 | A | C |
| rs4820827 | -6.99E-03 | 1.36E-02 | 0.608 | 0.623 | C | T |
| rs607703 | 1.33E-02 | 1.33E-02 | 0.320 | 0.469 | T | C |
| rs34536443 | 1.07E-02 | 3.34E-02 | 0.749 | 0.047 | C | G |
| rs11203203 | -1.03E-02 | 1.39E-02 | 0.458 | 0.357 | A | G |
| rs202535 | -1.52E-02 | 1.78E-02 | 0.392 | 0.824 | A | C |
| rs8046043 | -1.14E-02 | 1.36E-02 | 0.403 | 0.399 | C | G |
| rs28648882 | 8.51E-03 | 1.56E-02 | 0.585 | 0.221 | A | G |
| rs1881146 | -2.82E-02 | 1.45E-02 | 0.051 | 0.317 | T | A |
| rs13147049 | -6.98E-03 | 1.39E-02 | 0.616 | 0.651 | G | A |
| rs3087243 | 1.39E-02 | 1.35E-02 | 0.305 | 0.422 | A | G |
| rs9517712 | -4.12E-02 | 1.57E-02 | 0.009 | 0.756 | C | T |
| rs1947178 | 2.68E-03 | 1.61E-02 | 0.868 | 0.769 | G | A |
| rs17106304 | 1.42E-02 | 1.41E-02 | 0.313 | 0.670 | G | C |
| rs663743 | 2.04E-02 | 1.40E-02 | 0.146 | 0.328 | A | G |
| rs9260802 | 2.50E-02 | 3.51E-02 | 0.477 | 0.037 | G | A |
| rs1794269 | -1.17E-02 | 1.36E-02 | 0.392 | 0.375 | T | C |
| rs78325861 | -1.51E-02 | 3.56E-02 | 0.672 | 0.039 | G | C |
| rs7936434 | 3.74E-03 | 1.34E-02 | 0.779 | 0.478 | C | G |
| rs61759532 | -9.61E-03 | 1.75E-02 | 0.582 | 0.225 | T | C |
| rs113374757 | 4.22E-02 | 1.82E-02 | 0.020 | 0.165 | T | C |
| rs10751776 | 1.01E-02 | 1.34E-02 | 0.450 | 0.501 | C | A |
| rs17623914 | 2.26E-02 | 2.14E-02 | 0.290 | 0.105 | C | T |
| rs7511678 | -1.28E-02 | 1.62E-02 | 0.430 | 0.205 | A | G |
| rs10801128 | -2.38E-02 | 1.47E-02 | 0.106 | 0.707 | G | A |
| rs2111485 | -1.71E-02 | 1.37E-02 | 0.213 | 0.616 | G | A |
| rs55893453 | 1.59E-02 | 1.68E-02 | 0.343 | 0.197 | G | A |
| rs10224046 | 1.14E-03 | 1.42E-02 | 0.936 | 0.315 | G | T |
| rs2611211 | -1.75E-02 | 1.81E-02 | 0.335 | 0.833 | T | C |
| rs1050979 | 7.44E-03 | 1.32E-02 | 0.574 | 0.491 | G | A |
| rs7237497 | 6.74E-03 | 1.80E-02 | 0.708 | 0.845 | C | T |
| rs1350275 | 2.67E-02 | 1.46E-02 | 0.068 | 0.723 | G | T |
| rs56994090 | 1.77E-02 | 1.45E-02 | 0.223 | 0.419 | C | T |
| rs7130222 | -1.46E-02 | 1.41E-02 | 0.302 | 0.302 | G | T |
| rs1574285 | 9.38E-03 | 1.38E-02 | 0.495 | 0.574 | T | G |
| rs722988 | -7.39E-03 | 1.37E-02 | 0.590 | 0.367 | C | T |
| rs41295159 | 4.05E-03 | 6.70E-02 | 0.952 | 0.009 | G | C |
| rs2543537 | 2.42E-02 | 1.41E-02 | 0.086 | 0.431 | T | C |
| rs601338 | 2.34E-02 | 1.33E-02 | 0.078 | 0.460 | A | G |
| rs1611236 | 2.19E-02 | 1.40E-02 | 0.119 | 0.324 | A | G |
| rs112733823 | -2.15E-02 | 2.01E-02 | 0.285 | 0.127 | T | C |
| rs10275896 | 8.75E-03 | 1.54E-02 | 0.570 | 0.228 | C | T |

**Supplementary Table 19**

Summary information of genetic instruments associated with pancreatic cancer

| **SNP** | **Beta** | **SE** | **p-value** | **EAF** | **EA** | **NEA** |
| --- | --- | --- | --- | --- | --- | --- |
| rs574384 | -6.48E-02 | 5.19E-02 | 0.212 | 0.7204 | A | C |
| rs55893453 | -2.60E-02 | 4.98E-02 | 0.602 | 0.1573 | G | A |
| rs1050979 | -3.00E-03 | 3.53E-02 | 0.931 | 0.4674 | G | A |
| rs2303137 | -2.44E-02 | 3.60E-02 | 0.499 | 0.5196 | T | A |
| rs1808094 | 4.83E-02 | 3.92E-02 | 0.218 | 0.5335 | C | T |
| rs10224046 | -3.60E-03 | 4.38E-02 | 0.935 | 0.3296 | G | T |
| rs7776597 | 1.70E-01 | 1.05E-01 | 0.107 | 0.9600 | G | A |
| rs1701704 | -2.57E-02 | 3.84E-02 | 0.503 | 0.2993 | G | T |
| rs113374757 | -2.03E-02 | 5.11E-02 | 0.691 | 0.1804 | T | C |
| rs1881146 | -4.60E-02 | 4.17E-02 | 0.270 | 0.3353 | T | A |
| rs7795896 | -1.68E-02 | 4.44E-02 | 0.704 | 0.7666 | T | C |
| rs6434435 | 4.08E-02 | 4.85E-02 | 0.400 | 0.1551 | A | G |
| rs2111485 | -3.95E-02 | 3.75E-02 | 0.292 | 0.4958 | G | A |
| rs238873 | 3.85E-01 | 1.73E-01 | 0.026 | 0.0143 | G | A |
| rs9260802 | -3.45E-02 | 1.10E-01 | 0.754 | 0.0358 | G | A |
| rs9468618 | 2.09E-02 | 6.76E-02 | 0.758 | 0.0724 | T | C |
| rs28752526 | 5.04E-02 | 3.78E-02 | 0.183 | 0.3391 | G | A |
| rs12644686 | -1.90E-02 | 4.16E-02 | 0.648 | 0.2876 | G | C |
| rs2188962 | -6.55E-02 | 4.23E-02 | 0.121 | 0.2978 | T | C |
| rs10844597 | 1.10E-02 | 3.51E-02 | 0.753 | 0.4678 | A | G |
| rs9517712 | 8.61E-02 | 4.16E-02 | 0.038 | 0.7671 | C | T |
| rs12257077 | 3.16E-02 | 1.21E-01 | 0.794 | 0.0307 | T | C |
| rs1574285 | 1.26E-02 | 3.52E-02 | 0.720 | 0.5829 | T | G |
| rs13259300 | -4.37E-02 | 3.54E-02 | 0.217 | 0.5570 | C | A |
| rs7130222 | 3.01E-02 | 3.82E-02 | 0.431 | 0.2940 | G | T |
| rs55993634 | 1.58E-01 | 5.40E-02 | 0.003 | 0.1237 | G | C |
| rs8046043 | -4.00E-02 | 3.67E-02 | 0.276 | 0.3571 | C | G |
| rs35327136 | 1.06E-01 | 5.97E-02 | 0.075 | 0.1472 | A | C |
| rs663743 | -2.41E-02 | 3.79E-02 | 0.525 | 0.3153 | A | G |
| rs202535 | -1.34E-01 | 5.41E-02 | 0.013 | 0.8254 | A | C |
| rs229527 | 4.15E-02 | 3.55E-02 | 0.242 | 0.4758 | A | C |
| rs7511678 | -2.58E-02 | 3.89E-02 | 0.506 | 0.2889 | A | G |
| rs7668577 | 7.23E-02 | 4.44E-02 | 0.104 | 0.3117 | C | A |
| rs12464462 | -3.43E-02 | 4.18E-02 | 0.413 | 0.3069 | G | A |
| rs3135348 | 9.74E-02 | 4.35E-02 | 0.025 | 0.5523 | G | A |
| rs112733823 | 8.75E-02 | 5.30E-02 | 0.098 | 0.1299 | T | C |
| rs1611236 | 6.20E-03 | 3.87E-02 | 0.873 | 0.2902 | A | G |
| rs17623914 | 5.07E-02 | 7.01E-02 | 0.470 | 0.0977 | C | T |
| rs17323934 | -3.15E-02 | 4.51E-02 | 0.485 | 0.1901 | G | C |
| rs56994090 | -4.33E-02 | 3.51E-02 | 0.218 | 0.4283 | C | T |
| rs722988 | -1.08E-02 | 3.68E-02 | 0.770 | 0.4390 | C | T |
| rs11203203 | 2.80E-03 | 4.22E-02 | 0.947 | 0.2653 | A | G |
| rs2543537 | -8.90E-03 | 3.50E-02 | 0.800 | 0.4572 | T | C |
| rs7068821 | 3.08E-02 | 4.01E-02 | 0.442 | 0.2494 | T | G |
| rs78325861 | 1.33E-01 | 1.09E-01 | 0.222 | 0.0383 | G | C |
| rs238265 | 1.11E-02 | 3.89E-02 | 0.775 | 0.7164 | G | T |
| rs607703 | -4.71E-02 | 3.48E-02 | 0.175 | 0.4691 | T | C |
| rs601338 | -7.40E-03 | 4.17E-02 | 0.859 | 0.4719 | A | G |
| rs12742756 | -5.70E-03 | 3.54E-02 | 0.872 | 0.4099 | G | A |
| rs855330 | -8.40E-03 | 3.96E-02 | 0.831 | 0.2639 | C | T |
| rs3087243 | 1.70E-03 | 3.63E-02 | 0.962 | 0.3797 | A | G |
| rs28648882 | 3.31E-02 | 5.92E-02 | 0.576 | 0.2241 | A | G |
| rs4490209 | -2.79E-02 | 3.99E-02 | 0.485 | 0.4058 | G | C |
| rs4548024 | -3.02E-02 | 4.44E-02 | 0.496 | 0.2011 | C | T |
| rs1794269 | 2.36E-02 | 3.57E-02 | 0.510 | 0.4249 | T | C |
| rs6908236 | 2.00E-04 | 3.48E-02 | 0.996 | 0.5193 | C | A |
| rs114378220 | 1.09E-01 | 8.38E-02 | 0.192 | 0.0693 | T | C |
| rs2429557 | 6.06E-02 | 1.84E-01 | 0.742 | 0.0090 | A | T |
| rs1947178 | 6.40E-02 | 4.49E-02 | 0.154 | 0.8126 | G | A |
| rs689 | -4.39E-02 | 4.54E-02 | 0.333 | 0.7910 | T | A |
| rs12927355 | 6.04E-02 | 3.95E-02 | 0.126 | 0.2769 | T | C |
| rs41295159 | -3.64E-01 | 2.32E-01 | 0.116 | 0.0085 | G | C |
| rs61839660 | 8.00E-02 | 7.55E-02 | 0.289 | 0.0826 | T | C |
| rs231972 | 4.86E-02 | 6.46E-02 | 0.452 | 0.1154 | C | A |
| rs57209021 | -2.43E-02 | 4.96E-02 | 0.624 | 0.2242 | T | C |
| rs7237497 | -3.01E-02 | 4.93E-02 | 0.542 | 0.8534 | C | T |
| rs10275896 | -3.71E-02 | 4.46E-02 | 0.406 | 0.1964 | C | T |
| rs7936434 | -4.93E-02 | 4.13E-02 | 0.233 | 0.4603 | C | G |
| rs7301381 | -1.90E-02 | 3.46E-02 | 0.583 | 0.4707 | C | T |
| rs3184504 | -1.02E-02 | 4.14E-02 | 0.806 | 0.6547 | C | T |
| rs34536443 | 1.28E-01 | 1.06E-01 | 0.227 | 0.0418 | C | G |
| rs4820827 | -1.33E-02 | 3.64E-02 | 0.715 | 0.5443 | C | T |
| rs2493411 | 8.29E-02 | 5.63E-02 | 0.141 | 0.1119 | C | T |
| rs12128789 | 6.00E-04 | 6.11E-02 | 0.993 | 0.1311 | C | T |
| rs10801128 | 4.15E-02 | 3.76E-02 | 0.269 | 0.6789 | G | A |
| rs3024493 | 5.95E-02 | 5.56E-02 | 0.285 | 0.1193 | A | C |
| rs6679677 | -8.22E-02 | 6.45E-02 | 0.203 | 0.1152 | A | C |
| rs34593439 | 8.78E-02 | 5.95E-02 | 0.140 | 0.0973 | A | G |
| rs17106304 | -4.97E-02 | 3.68E-02 | 0.177 | 0.6676 | G | C |
| rs10751776 | -4.40E-03 | 3.49E-02 | 0.900 | 0.5394 | C | A |
| rs3802214 | 1.63E-02 | 3.99E-02 | 0.682 | 0.7280 | C | T |
| rs9385401 | -9.40E-03 | 4.91E-02 | 0.848 | 0.6257 | T | C |
| rs2611211 | -7.00E-04 | 4.16E-02 | 0.986 | 0.7704 | T | C |
| rs13147049 | 8.00E-04 | 3.57E-02 | 0.981 | 0.6009 | G | A |
| rs3135348 | -9.64E-01 | 1.24E+00 | 0.438 | 0.0010 | T | A |
| rs7752257 | -4.27E-02 | 4.14E-02 | 0.302 | 0.7631 | G | T |
| rs6908626 | 6.34E-02 | 5.68E-02 | 0.264 | 0.1610 | T | G |
| rs73432769 | 1.48E-01 | 9.43E-02 | 0.117 | 0.0328 | T | C |
| rs61759532 | -1.26E-02 | 4.75E-02 | 0.791 | 0.1905 | T | C |
| rs1350275 | -2.42E-02 | 3.68E-02 | 0.510 | 0.6255 | G | T |

**Supplementary Table 20**

Summary information of genetic instruments associated with prostate cancer

| **SNP** | **Beta** | **SE** | **p-value** | **EAF** | **EA** | **NEA** |
| --- | --- | --- | --- | --- | --- | --- |
| rs855330 | 3.70E-03 | 9.60E-03 | 0.696 | 0.244 | C | T |
| rs6434435 | 4.30E-03 | 1.15E-02 | 0.706 | 0.165 | A | G |
| rs7511678 | 6.40E-03 | 9.70E-03 | 0.512 | 0.218 | A | G |
| rs1574285 | -2.18E-02 | 8.50E-03 | 0.010 | 0.580 | T | G |
| rs114378220 | -2.20E-03 | 1.96E-02 | 0.910 | 0.058 | T | C |
| rs9385401 | 9.00E-03 | 8.00E-03 | 0.260 | 0.463 | T | C |
| rs7068821 | 2.10E-02 | 9.10E-03 | 0.022 | 0.251 | T | G |
| rs10844597 | -1.30E-03 | 8.10E-03 | 0.876 | 0.497 | A | G |
| rs7237497 | -2.20E-03 | 1.09E-02 | 0.844 | 0.837 | C | T |
| rs202535 | 2.20E-03 | 1.09E-02 | 0.841 | 0.830 | A | C |
| rs9517712 | -1.49E-02 | 9.40E-03 | 0.112 | 0.753 | C | T |
| rs34593439 | 3.80E-03 | 1.38E-02 | 0.782 | 0.107 | A | G |
| rs1611236 | -4.27E-02 | 8.60E-03 | 0.000 | 0.309 | A | G |
| rs11203203 | 7.00E-03 | 8.60E-03 | 0.417 | 0.349 | A | G |
| rs2543537 | 5.50E-03 | 8.70E-03 | 0.525 | 0.430 | T | C |
| rs229527 | -3.00E-03 | 8.20E-03 | 0.712 | 0.416 | A | C |
| rs57209021 | -1.02E-02 | 1.01E-02 | 0.315 | 0.219 | T | C |
| rs4490209 | -8.50E-03 | 8.70E-03 | 0.332 | 0.356 | G | C |
| rs2611211 | -7.00E-04 | 1.08E-02 | 0.949 | 0.820 | T | C |
| rs28648882 | -9.80E-03 | 9.50E-03 | 0.304 | 0.228 | A | G |
| rs2303137 | 1.80E-03 | 8.00E-03 | 0.818 | 0.439 | T | A |
| rs12644686 | 8.80E-03 | 1.13E-02 | 0.436 | 0.188 | G | C |
| rs238873 | 6.76E-02 | 3.61E-02 | 0.061 | 0.012 | G | A |
| rs10275896 | -2.58E-02 | 9.40E-03 | 0.006 | 0.233 | C | T |
| rs10224046 | 4.28E-02 | 8.60E-03 | 0.000 | 0.325 | G | T |
| rs9260802 | -1.21E-02 | 2.13E-02 | 0.568 | 0.036 | G | A |
| rs2429557 | -3.50E-03 | 4.01E-02 | 0.931 | 0.010 | A | T |
| rs1794269 | -1.35E-02 | 8.10E-03 | 0.097 | 0.394 | T | C |
| rs1050979 | -1.66E-02 | 8.00E-03 | 0.037 | 0.492 | G | A |
| rs3184504 | 1.87E-02 | 8.20E-03 | 0.022 | 0.519 | C | T |
| rs7936434 | -2.88E-02 | 8.10E-03 | 0.000 | 0.467 | C | G |
| rs78325861 | 1.11E-02 | 2.18E-02 | 0.611 | 0.041 | G | C |
| rs61839660 | -2.05E-02 | 1.43E-02 | 0.151 | 0.087 | T | C |
| rs1701704 | 1.04E-02 | 8.50E-03 | 0.220 | 0.326 | G | T |
| rs7301381 | 1.71E-02 | 8.10E-03 | 0.035 | 0.454 | C | T |
| rs55993634 | -9.30E-03 | 1.53E-02 | 0.545 | 0.090 | G | C |
| rs4820827 | 2.80E-02 | 8.30E-03 | 0.001 | 0.623 | C | T |
| rs231972 | 1.31E-02 | 1.36E-02 | 0.334 | 0.119 | C | A |
| rs34536443 | 3.98E-02 | 2.21E-02 | 0.073 | 0.039 | C | G |
| rs3802214 | 4.40E-03 | 1.03E-02 | 0.665 | 0.806 | C | T |
| rs722988 | -4.60E-03 | 8.40E-03 | 0.582 | 0.365 | C | T |
| rs41295159 | -6.22E-02 | 4.14E-02 | 0.133 | 0.011 | G | C |
| rs9468618 | 2.37E-02 | 1.43E-02 | 0.097 | 0.083 | T | C |
| rs3135348 | 2.01E-02 | 8.00E-03 | 0.012 | 0.573 | G | A |
| rs73432769 | -1.52E-02 | 2.75E-02 | 0.580 | 0.022 | T | C |
| rs2493411 | -2.47E-02 | 1.20E-02 | 0.040 | 0.130 | C | T |
| rs1881146 | -2.10E-03 | 9.00E-03 | 0.812 | 0.310 | T | A |
| rs10801128 | -2.06E-02 | 9.10E-03 | 0.023 | 0.719 | G | A |
| rs13147049 | 2.60E-03 | 8.30E-03 | 0.755 | 0.646 | G | A |
| rs2111485 | 4.00E-03 | 8.40E-03 | 0.636 | 0.601 | G | A |
| rs2188962 | 7.90E-03 | 8.10E-03 | 0.328 | 0.407 | T | C |
| rs61759532 | 8.60E-03 | 1.05E-02 | 0.413 | 0.216 | T | C |
| rs35327136 | -2.90E-02 | 1.08E-02 | 0.007 | 0.167 | A | C |
| rs113374757 | -1.00E-04 | 1.12E-02 | 0.990 | 0.160 | T | C |
| rs12464462 | 6.10E-03 | 8.20E-03 | 0.455 | 0.405 | G | A |
| rs7668577 | -5.10E-03 | 8.80E-03 | 0.563 | 0.310 | C | A |
| rs4548024 | -1.59E-02 | 1.01E-02 | 0.116 | 0.228 | C | T |
| rs112733823 | 5.13E-02 | 1.15E-02 | 0.000 | 0.138 | T | C |
| rs12257077 | 3.46E-02 | 2.11E-02 | 0.101 | 0.038 | T | C |
| rs6908236 | 7.70E-03 | 7.90E-03 | 0.333 | 0.509 | C | A |
| rs663743 | 6.00E-04 | 8.60E-03 | 0.942 | 0.342 | A | G |
| rs238265 | -2.17E-02 | 8.80E-03 | 0.014 | 0.701 | G | T |
| rs13259300 | -6.10E-03 | 8.40E-03 | 0.463 | 0.623 | C | A |
| rs17106304 | -8.00E-04 | 8.50E-03 | 0.928 | 0.663 | G | C |
| rs7130222 | 1.54E-02 | 8.70E-03 | 0.076 | 0.314 | G | T |
| rs1350275 | 1.74E-02 | 8.80E-03 | 0.048 | 0.698 | G | T |
| rs601338 | 9.00E-03 | 8.20E-03 | 0.270 | 0.462 | A | G |
| rs12927355 | 4.40E-03 | 8.80E-03 | 0.613 | 0.321 | T | C |
| rs6679677 | 1.60E-02 | 1.34E-02 | 0.235 | 0.100 | A | C |
| rs3087243 | -1.80E-03 | 8.20E-03 | 0.830 | 0.429 | A | G |
| rs3135348 | 9.00E-03 | 8.33E-02 | 0.914 | 0.002 | T | A |
| rs7752257 | -3.88E-02 | 9.10E-03 | 0.000 | 0.747 | G | T |
| rs28752526 | 1.70E-02 | 8.30E-03 | 0.042 | 0.336 | G | A |
| rs55893453 | 1.76E-02 | 1.06E-02 | 0.097 | 0.198 | G | A |
| rs10751776 | -1.28E-02 | 8.20E-03 | 0.119 | 0.509 | C | A |
| rs574384 | -5.70E-03 | 1.32E-02 | 0.668 | 0.888 | A | C |
| rs12742756 | 4.30E-03 | 8.70E-03 | 0.617 | 0.430 | G | A |
| rs3024493 | -6.30E-03 | 1.10E-02 | 0.565 | 0.156 | A | C |
| rs17623914 | -3.20E-02 | 1.34E-02 | 0.017 | 0.102 | C | T |
| rs12128789 | 1.56E-02 | 1.23E-02 | 0.206 | 0.136 | C | T |
| rs7795896 | -2.12E-02 | 8.70E-03 | 0.015 | 0.692 | T | C |
| rs17323934 | -2.72E-02 | 9.60E-03 | 0.005 | 0.225 | G | C |
| rs6908626 | -3.60E-03 | 1.22E-02 | 0.767 | 0.159 | T | G |
| rs1947178 | -1.48E-02 | 1.01E-02 | 0.143 | 0.783 | G | A |
| rs689 | -4.51E-02 | 9.00E-03 | 0.000 | 0.725 | T | A |
| rs56994090 | -2.60E-03 | 8.90E-03 | 0.766 | 0.422 | C | T |
| rs1808094 | 9.00E-03 | 8.10E-03 | 0.264 | 0.525 | C | T |
| rs8046043 | 1.60E-03 | 8.20E-03 | 0.846 | 0.392 | C | G |
| rs7776597 | 8.60E-03 | 2.05E-02 | 0.677 | 0.959 | G | A |
| rs607703 | 5.10E-03 | 8.10E-03 | 0.533 | 0.477 | T | C |

**Supplementary Table 21**

Summary information of genetic instruments associated with non-melanoma skin cancer

| **SNP** | **Beta** | **SE** | **p-value** | **EAF** | **EA** | **NEA** |
| --- | --- | --- | --- | --- | --- | --- |
| rs1701704 | -2.15E-04 | 0.000122 | 0.077 | 0.341 | G | T |
| rs56994090 | 3.89E-05 | 0.000117 | 0.740 | 0.415 | C | T |
| rs12742756 | -3.78E-05 | 0.000117 | 0.750 | 0.427 | G | A |
| rs7668577 | -1.49E-05 | 0.000125 | 0.910 | 0.311 | C | A |
| rs6908236 | -1.02E-04 | 0.000116 | 0.380 | 0.530 | C | A |
| rs3135348 | 1.27E-04 | 0.000116 | 0.280 | 0.559 | G | A |
| rs61759532 | -1.54E-04 | 0.000138 | 0.260 | 0.246 | T | C |
| rs663743 | 2.16E-04 | 0.000122 | 0.077 | 0.341 | A | G |
| rs7068821 | -2.62E-04 | 0.000132 | 0.048 | 0.257 | T | G |
| rs2188962 | 2.62E-04 | 0.000117 | 0.025 | 0.428 | T | C |
| rs1050979 | 3.07E-04 | 0.000115 | 0.008 | 0.528 | G | A |
| rs9385401 | -1.64E-05 | 0.000116 | 0.890 | 0.456 | T | C |
| rs10275896 | 9.12E-05 | 0.000135 | 0.500 | 0.245 | C | T |
| rs11203203 | -1.25E-04 | 0.00012 | 0.300 | 0.367 | A | G |
| rs10751776 | -1.92E-04 | 0.000116 | 0.097 | 0.500 | C | A |
| rs1881146 | -9.02E-05 | 0.000126 | 0.470 | 0.313 | T | A |
| rs2111485 | 3.80E-05 | 0.000118 | 0.750 | 0.607 | G | A |
| rs3087243 | 2.02E-04 | 0.000116 | 0.082 | 0.451 | A | G |
| rs2303137 | -1.23E-06 | 0.000117 | 0.990 | 0.434 | T | A |
| rs9517712 | -1.46E-04 | 0.000134 | 0.280 | 0.749 | C | T |
| rs7130222 | 8.87E-05 | 0.000124 | 0.470 | 0.325 | G | T |
| rs4820827 | -3.52E-05 | 0.000118 | 0.770 | 0.606 | C | T |
| rs238265 | 2.55E-04 | 0.000127 | 0.044 | 0.702 | G | T |
| rs3184504 | 2.59E-04 | 0.000116 | 0.025 | 0.517 | C | T |
| rs12927355 | -1.03E-04 | 0.000124 | 0.400 | 0.323 | T | C |
| rs17106304 | -1.92E-05 | 0.000122 | 0.870 | 0.656 | G | C |
| rs689 | -6.84E-05 | 0.000128 | 0.590 | 0.711 | T | A |
| rs7936434 | 5.19E-05 | 0.000116 | 0.650 | 0.478 | C | G |
| rs607703 | -1.51E-04 | 0.000116 | 0.190 | 0.473 | T | C |
| rs2543537 | 1.12E-04 | 0.000117 | 0.340 | 0.444 | T | C |
| rs7795896 | -2.33E-04 | 0.000127 | 0.067 | 0.705 | T | C |
| rs13259300 | 2.30E-04 | 0.00012 | 0.054 | 0.610 | C | A |
| rs13147049 | -7.45E-06 | 0.000123 | 0.950 | 0.671 | G | A |
| rs12464462 | 2.52E-04 | 0.000118 | 0.032 | 0.414 | G | A |
| rs4490209 | 1.60E-04 | 0.00012 | 0.180 | 0.362 | G | C |
| rs10801128 | -1.20E-04 | 0.000127 | 0.350 | 0.710 | G | A |
| rs601338 | -3.47E-04 | 0.000115 | 0.003 | 0.508 | A | G |
| rs229527 | -2.74E-05 | 0.000117 | 0.810 | 0.423 | A | C |
| rs722988 | 7.22E-05 | 0.00012 | 0.550 | 0.367 | C | T |
| rs1574285 | -2.31E-05 | 0.000118 | 0.840 | 0.599 | T | G |
| rs10224046 | -9.37E-05 | 0.000125 | 0.450 | 0.310 | G | T |
| rs8046043 | -3.05E-04 | 0.000117 | 0.009 | 0.416 | C | G |
| rs10844597 | 2.02E-05 | 0.000116 | 0.860 | 0.497 | A | G |
| rs1350275 | 1.88E-04 | 0.000129 | 0.150 | 0.722 | G | T |
| rs7301381 | -2.08E-06 | 0.000116 | 0.990 | 0.465 | C | T |
| rs7752257 | 1.18E-04 | 0.000134 | 0.380 | 0.751 | G | T |
| rs28752526 | -2.44E-04 | 0.000121 | 0.043 | 0.358 | G | A |
| rs1611236 | 2.06E-05 | 0.000123 | 0.870 | 0.324 | A | G |
| rs1808094 | 2.03E-04 | 0.000116 | 0.080 | 0.525 | C | T |

**Supplementary Table 22**

Summary information of genetic instruments associated with thyroid cancer

| **SNP** | **Beta** | **SE** | **p-value** | **EAF** | **EA** | **NEA** |
| --- | --- | --- | --- | --- | --- | --- |
| rs3024493 | 2.10E-02 | 5.95E-02 | 0.723 | 0.1172 | A | C |
| rs2111485 | 5.90E-03 | 4.05E-02 | 0.884 | 0.4895 | G | A |
| rs1611236 | 6.10E-03 | 4.32E-02 | 0.888 | 0.2907 | A | G |
| rs2303137 | -3.06E-02 | 3.91E-02 | 0.433 | 0.5238 | T | A |
| rs7776597 | -2.92E-01 | 1.17E-01 | 0.012 | 0.9597 | G | A |
| rs10224046 | -9.50E-03 | 4.63E-02 | 0.837 | 0.3289 | G | T |
| rs9260802 | 1.52E-01 | 1.17E-01 | 0.195 | 0.0358 | G | A |
| rs2429557 | -1.65E-01 | 2.12E-01 | 0.436 | 0.0090 | A | T |
| rs112733823 | -3.33E-02 | 5.57E-02 | 0.549 | 0.1281 | T | C |
| rs13259300 | -3.20E-02 | 3.86E-02 | 0.408 | 0.5550 | C | A |
| rs2188962 | -2.63E-02 | 4.55E-02 | 0.563 | 0.2925 | T | C |
| rs11203203 | -7.40E-03 | 4.57E-02 | 0.872 | 0.2613 | A | G |
| rs113374757 | -1.62E-02 | 6.13E-02 | 0.791 | 0.1812 | T | C |
| rs3184504 | 7.30E-02 | 4.42E-02 | 0.099 | 0.6614 | C | T |
| rs722988 | 9.10E-03 | 4.04E-02 | 0.822 | 0.4449 | C | T |
| rs61839660 | -1.43E-01 | 8.54E-02 | 0.095 | 0.0831 | T | C |
| rs7936434 | 1.80E-03 | 4.41E-02 | 0.967 | 0.4607 | C | G |
| rs55993634 | -4.39E-02 | 5.96E-02 | 0.462 | 0.1261 | G | C |
| rs12742756 | 3.35E-02 | 3.85E-02 | 0.384 | 0.4080 | G | A |
| rs1881146 | 2.35E-02 | 5.00E-02 | 0.639 | 0.3361 | T | A |
| rs10801128 | 2.32E-02 | 4.14E-02 | 0.574 | 0.6762 | G | A |
| rs1050979 | 1.77E-02 | 3.84E-02 | 0.645 | 0.4649 | G | A |
| rs17323934 | 2.63E-02 | 4.94E-02 | 0.595 | 0.1887 | G | C |
| rs607703 | 1.33E-02 | 3.80E-02 | 0.727 | 0.4676 | T | C |
| rs3135348 | -7.06E-02 | 4.77E-02 | 0.139 | 0.5526 | G | A |
| rs1794269 | 1.91E-02 | 3.92E-02 | 0.626 | 0.4237 | T | C |
| rs1350275 | 8.30E-03 | 3.97E-02 | 0.835 | 0.6223 | G | T |
| rs57209021 | -5.33E-02 | 5.32E-02 | 0.316 | 0.2243 | T | C |
| rs601338 | 2.79E-02 | 4.45E-02 | 0.531 | 0.4735 | A | G |
| rs574384 | -4.87E-02 | 5.64E-02 | 0.388 | 0.7099 | A | C |
| rs2493411 | 9.38E-02 | 5.94E-02 | 0.115 | 0.1102 | C | T |
| rs238873 | 1.99E-01 | 1.82E-01 | 0.274 | 0.0143 | G | A |
| rs9468618 | 2.16E-02 | 7.55E-02 | 0.775 | 0.0721 | T | C |
| rs6908236 | 1.19E-02 | 3.80E-02 | 0.755 | 0.5206 | C | A |
| rs2611211 | 6.90E-03 | 4.51E-02 | 0.878 | 0.7677 | T | C |
| rs28648882 | -6.58E-02 | 7.23E-02 | 0.363 | 0.2241 | A | G |
| rs4490209 | -1.77E-02 | 4.79E-02 | 0.713 | 0.4079 | G | C |
| rs17106304 | 5.08E-02 | 3.99E-02 | 0.204 | 0.6684 | G | C |
| rs56994090 | -2.85E-02 | 3.81E-02 | 0.455 | 0.4268 | C | T |
| rs10844597 | 3.39E-02 | 3.83E-02 | 0.377 | 0.4650 | A | G |
| rs7301381 | -8.90E-03 | 3.79E-02 | 0.815 | 0.4711 | C | T |
| rs1574285 | 1.55E-02 | 3.84E-02 | 0.687 | 0.5828 | T | G |
| rs8046043 | 6.12E-02 | 4.02E-02 | 0.128 | 0.3561 | C | G |
| rs202535 | -5.43E-02 | 5.65E-02 | 0.337 | 0.8259 | A | C |
| rs229527 | 1.60E-02 | 3.87E-02 | 0.679 | 0.4799 | A | C |
| rs55893453 | 1.11E-02 | 5.43E-02 | 0.839 | 0.1549 | G | A |
| rs6434435 | 8.04E-02 | 5.43E-02 | 0.139 | 0.1556 | A | G |
| rs114378220 | 5.37E-02 | 9.02E-02 | 0.552 | 0.0695 | T | C |
| rs7668577 | 6.77E-02 | 4.73E-02 | 0.153 | 0.3117 | C | A |
| rs7511678 | -3.59E-02 | 4.19E-02 | 0.393 | 0.2914 | A | G |
| rs12464462 | -3.56E-02 | 4.47E-02 | 0.425 | 0.3007 | G | A |
| rs7795896 | -8.06E-02 | 4.69E-02 | 0.086 | 0.7717 | T | C |
| rs10275896 | 2.95E-02 | 4.88E-02 | 0.546 | 0.1949 | C | T |
| rs9385401 | 6.00E-04 | 5.99E-02 | 0.992 | 0.6336 | T | C |
| rs4548024 | 4.60E-02 | 4.82E-02 | 0.340 | 0.1994 | C | T |
| rs689 | 1.37E-02 | 4.96E-02 | 0.783 | 0.7941 | T | A |
| rs7752257 | 3.16E-02 | 4.53E-02 | 0.486 | 0.7642 | G | T |
| rs41295159 | 4.54E-01 | 2.73E-01 | 0.097 | 0.0086 | G | C |
| rs663743 | -5.33E-02 | 4.11E-02 | 0.194 | 0.3128 | A | G |
| rs4820827 | 8.70E-03 | 3.99E-02 | 0.827 | 0.5387 | C | T |
| rs1808094 | 5.39E-02 | 4.71E-02 | 0.252 | 0.5343 | C | T |
| rs34536443 | -2.09E-01 | 1.16E-01 | 0.071 | 0.0419 | C | G |
| rs35327136 | -2.20E-02 | 6.84E-02 | 0.747 | 0.1484 | A | C |
| rs34593439 | 2.43E-02 | 6.37E-02 | 0.702 | 0.0963 | A | G |
| rs855330 | 1.49E-02 | 4.26E-02 | 0.726 | 0.2628 | C | T |
| rs3087243 | -2.18E-02 | 3.98E-02 | 0.583 | 0.3783 | A | G |
| rs10751776 | -1.30E-02 | 3.81E-02 | 0.732 | 0.5405 | C | A |
| rs17623914 | -8.91E-02 | 7.70E-02 | 0.247 | 0.0980 | C | T |
| rs6679677 | 1.34E-01 | 6.66E-02 | 0.044 | 0.1148 | A | C |
| rs12644686 | -3.36E-02 | 4.51E-02 | 0.457 | 0.2927 | G | C |
| rs12128789 | 5.38E-02 | 6.59E-02 | 0.415 | 0.1313 | C | T |
| rs28752526 | -7.63E-02 | 4.22E-02 | 0.071 | 0.3388 | G | A |
| rs1947178 | 4.50E-03 | 4.98E-02 | 0.927 | 0.8129 | G | A |
| rs13147049 | -7.65E-02 | 3.87E-02 | 0.048 | 0.5998 | G | A |
| rs3135348 | -1.04E+00 | 1.01E+00 | 0.301 | 0.0010 | T | A |
| rs73432769 | 1.65E-02 | 1.07E-01 | 0.877 | 0.0337 | T | C |
| rs12257077 | -8.60E-03 | 1.34E-01 | 0.948 | 0.0309 | T | C |
| rs3802214 | -6.24E-02 | 4.33E-02 | 0.150 | 0.7247 | C | T |
| rs6908626 | 2.79E-02 | 6.21E-02 | 0.653 | 0.1616 | T | G |
| rs238265 | -1.22E-02 | 4.22E-02 | 0.772 | 0.7181 | G | T |
| rs9517712 | 3.27E-02 | 4.49E-02 | 0.466 | 0.7690 | C | T |
| rs7130222 | -9.20E-03 | 4.19E-02 | 0.826 | 0.2937 | G | T |
| rs1701704 | -4.57E-02 | 4.18E-02 | 0.274 | 0.2971 | G | T |
| rs12927355 | -3.53E-02 | 4.29E-02 | 0.411 | 0.2746 | T | C |
| rs231972 | -3.15E-02 | 7.04E-02 | 0.654 | 0.1156 | C | A |
| rs7237497 | -2.50E-03 | 5.42E-02 | 0.964 | 0.8540 | C | T |
| rs61759532 | -2.50E-03 | 5.15E-02 | 0.961 | 0.1884 | T | C |
| rs7068821 | -4.34E-02 | 4.40E-02 | 0.324 | 0.2498 | T | G |
| rs78325861 | -1.84E-01 | 1.17E-01 | 0.116 | 0.0384 | G | C |
| rs2543537 | 1.43E-02 | 3.82E-02 | 0.708 | 0.4562 | T | C |

**Supplementary Table 23**

Summary information of IVs associated with bladder cancer.

| **SNP** | **Beta** | **SE** | **p-value** | **EAF** | **EA** | **NEA** |
| --- | --- | --- | --- | --- | --- | --- |
| rs10844597 | -0.0002 | 0.0001 | 0.057 | 0.4969 | A | G |
| rs607703 | 0 | 0.0001 | 0.93 | 0.4728 | T | C |
| rs4490209 | -0.0001 | 0.0001 | 0.51 | 0.3623 | G | C |
| rs12927355 | 0.0001 | 0.0001 | 0.41 | 0.3226 | T | C |
| rs17106304 | -0.0002 | 0.0001 | 0.084 | 0.6562 | G | C |
| rs7301381 | 0.0001 | 0.0001 | 0.19 | 0.4652 | C | T |
| rs2188962 | 0 | 0.0001 | 0.77 | 0.4277 | T | C |
| rs1050979 | 0 | 0.0001 | 0.75 | 0.5283 | G | A |
| rs6908236 | -0.0001 | 0.0001 | 0.3 | 0.5304 | C | A |
| rs3087243 | 0 | 0.0001 | 0.97 | 0.4507 | A | G |
| rs4820827 | 0 | 0.0001 | 0.91 | 0.6061 | C | T |
| rs663743 | 0 | 0.0001 | 0.73 | 0.3405 | A | G |
| rs3135348 | 0 | 0.0001 | 0.73 | 0.5593 | G | A |
| rs9385401 | 0 | 0.0001 | 0.75 | 0.4558 | T | C |
| rs722988 | 0 | 0.0001 | 0.98 | 0.3667 | C | T |
| rs1574285 | -0.0001 | 0.0001 | 0.29 | 0.5988 | T | G |
| rs3184504 | 0 | 0.0001 | 0.93 | 0.5173 | C | T |
| rs8046043 | 0 | 0.0001 | 0.93 | 0.4158 | C | G |
| rs229527 | 0.0001 | 0.0001 | 0.45 | 0.4234 | A | C |
| rs1701704 | -0.0001 | 0.0001 | 0.28 | 0.3413 | G | T |
| rs12742756 | 0 | 0.0001 | 0.7 | 0.427 | G | A |
| rs7130222 | 0.0002 | 0.0001 | 0.024 | 0.3253 | G | T |
| rs13147049 | 0.0001 | 0.0001 | 0.23 | 0.6714 | G | A |
| rs13259300 | -0.0001 | 0.0001 | 0.31 | 0.6103 | C | A |
| rs12464462 | -0.0001 | 0.0001 | 0.55 | 0.4142 | G | A |
| rs2543537 | 0.0002 | 0.0001 | 0.072 | 0.444 | T | C |
| rs601338 | 0 | 0.0001 | 0.93 | 0.5078 | A | G |
| rs2303137 | -0.0001 | 0.0001 | 0.44 | 0.4335 | T | A |
| rs11203203 | 0 | 0.0001 | 0.96 | 0.3674 | A | G |
| rs56994090 | -0.0002 | 0.0001 | 0.017 | 0.415 | C | T |
| rs7936434 | -0.0002 | 0.0001 | 0.084 | 0.4781 | C | G |
| rs10751776 | 0.0002 | 0.0001 | 0.11 | 0.5004 | C | A |
| rs2111485 | -0.0001 | 0.0001 | 0.49 | 0.6067 | G | A |
| rs28752526 | 0 | 0.0001 | 0.91 | 0.3583 | G | A |
| rs1611236 | -0.0001 | 0.0001 | 0.41 | 0.3244 | A | G |
| rs1808094 | -0.0002 | 0.0001 | 0.056 | 0.5246 | C | T |

Abbreviations: SNPs: single nucleotide polymorphisms; NEA: non-effect allele; EAF:effect allele frequency; EA: effect allele; SE: standard error.

**Supplementary Table 24**

Steiger directionality test from type 1 diabetes to 22 site-specific cancer

| **Exposure** | **Outcome** | **SNP-R2.exposure** | **SNP**  **_R2.outcome** | **Correct causal direction** | **Steiger P value** |
| --- | --- | --- | --- | --- | --- |
| Type 1 diabetes | Colorectal cancer | 0.020552569 | 0.003481282 | TRUE | 1.04E-49 |
|  | Pancreatic cancer | 0.022367879 | 0.000207808 | TRUE | 0.00E+00 |
|  | Gastric cancer | 0.022367879 | 0.000244938 | TRUE | 0.00E+00 |
|  | Hepatic cancer | 0.022367879 | 0.000223839 | TRUE | 0.00E+00 |
|  | Cholangiocarcinoma | 0.022367879 | 0.000190983 | TRUE | 0.00E+00 |
|  | Esophageal cancer | 0.016341286 | 0.000208532 | TRUE | 0.00E+00 |
|  | Breast cancer | 0.020866641 | 0.001301942 | TRUE | 1.43E-189 |
|  | Ovarian cancer | 0.019114432 | 0.001446709 | TRUE | 5.57E-133 |
|  | Cervical cancer | 0.017548028 | 0.000312277 | TRUE | 0.00E+00 |
|  | Endometrial cancer | 0.021094924 | 0.000923978 | TRUE | 2.65E-290 |
|  | Thyroid cancer | 0.022367879 | 0.000158481 | TRUE | 0.00E+00 |
|  | Lung cancer | 0.019289414 | 0.00228377 | TRUE | 2.21E-137 |
|  | Kidney cancer | 0.010645602 | 0.000115952 | TRUE | 0.00E+00 |
|  | Bladder cancer | 0.008703294 | 9.05E-05 | TRUE | 0.00E+00 |
|  | Lymphoma | 0.020553509 | 0.000369118 | TRUE | 0.00E+00 |
|  | Multiple myeloma | 0.165984472 | 0.000127483 | TRUE | 0.00E+00 |
|  | Prostate cancer | 0.015710241 | 0.001033215 | TRUE | 1.26E-213 |
|  | Brain tumor | 0.022367879 | 0.000151855 | TRUE | 0.00E+00 |
|  | Malignant melanoma | 0.018250519 | 0.000256286 | TRUE | 0.00E+00 |
|  | Skin cancer | 0.011128689 | 0.000141589 | TRUE | 0.00E+00 |
|  | Leukeamia | 0.016036775 | 0.000210373 | TRUE | 0.00E+00 |
|  | Head and neck cancer | 0.018783815 | 0.000204069 | TRUE | 0.00E+00 |

Abbreviations: SNPs: single nucleotide polymorphisms; R2: R^2^:Proportion of variance
