# Supplementary material for: A two-sample Mendelian randomization study of type 1 diabetes and the risk of 22 site-specific cancers
Source: Sci Rep. 2025 Apr 3;15:11371. doi: 10.1038/s41598-025-89288-3 (PMC11965372; doi:10.1038/s41598-025-89288-3)
Supplement: Supplementary file 3 — Supplementary Information 3. [file 41598_2025_89288_MOESM3_ESM.docx]

**STROBE-MR checklist.**

| **Item No.** | **Section** | **Checklist item** | **Page No.** | **Relevant text from manuscript** |
| --- | --- | --- | --- | --- |
| 1 | **Title and abstract** | Indicate Mendelian randomization (MR) as the study’s design in the title and/or the abstract if that is a main purpose of the study | 1，2 | Causal relationships between type 1 diabetes and risk of 22 site-specific cancers: A two-sample Mendelian randomization study |
|  | **Introduction** |  |  |  |
| 2 | **Background** | Explain the scientific background and rationale for the reported study. What is the exposure? Is a potential causal relationship between exposure and outcome plausible? Justify why MR is a helpful method to address the study question | 3，4 | The previous studies have limitations due to their non-randomized design, making them susceptible to confounding factors, reverse causation, and inherent biases in observational research design. [10] Therefore, there is a compelling need for rigorous causal inference methods to comprehensively understand the nature of the association between T1D and cancer for effective prevention and management, leading to a significant reduction in disease burden.  Mendelian randomization (MR) emerges as a powerful tool in this context, to assess causal relationships using genetic variants as instrumental variables. It overcomes the methdological limitation of traditional observational study using genetic variants that are robustly associated with exposures like T1D as proxies. As genetic variants are randomly assorted at conception and fixed for life time, MR minimizes confounding and reverse causation, providing more reliable estimates of causal effects. [11,12] Therefore, the MR approach is conceptually similar to a randomized controlled trial (RCT) but being more feasible and cost-effective. [13] |
| 3 | **Objectives** | State specific objectives clearly, including pre-specified causal hypotheses (if any). State that MR is a method that, under specific assumptions, intends to estimate causal effects | 4,5 | This study aimed to investigate the causal association between T1D and 22 site-specific cancers using large-scale genome-wide association studies (GWAS) data. |
|  | **METHODS** |  |  |  |
| 4 | **Study design and data sources** | Present key elements of the study design early in the article. Consider including a table listing sources of data for all phases of the study. For each data source contributing to the analysis, describe the following: | 6-9 | See Figure 1 and Table 1 |
|  | a) | Setting: Describe the study design and the underlying population, if possible. Describe the setting, locations, and relevant dates, including periods of recruitment, exposure, follow-up, and data collection, when available. | 5, 6 | We conducted a two-sample MR study using a single-nucleotide polymorphisms (SNPs) with T1D as instrument variable to assess the causal relationships with 22 site-specific cancer. [12] The study design is illustrated in Figure 1 below. |
|  | b) | Participants: Give the eligibility criteria, and the sources and methods of selection of participants. Report the sample size, and whether any power or sample size calculations were carried out prior to the main analysis | 7，8 | We used publicly available large summary data from genome-wide association study (GWAS) database. All identified genetic variants of T1D used the data from the largest and latest GWAS study, in which 18,942 T1D patients and 501,638 control participants of European ancestry (Finland, Republic of Ireland, U.K.) from nine cohorts were enrolled. The total sample size was 520,580 participants with 60 million number of SNPs. 16 To our knowledge it is the largest and latest GWAS summary data available for T1D.  We have identified 22 site-specific cancer for which summary level data acquired from openGWAS database accessed on April 4, 2024. [17–19] The 22 site-specfic cancer includes： colorectal cancer, pancreatic cancer, gastric cancer, hepatic cancer, cholangiocarcinoma, esophageal cancer, breast cancer, ovarian cancer, cervical cancer, endometrial cancer, brain cancer, thyroid cancer, lung cancer, malignant neoplasm of kidney, bladder cancer, lymphoma, leukaemia, head and neck cancer, multiple myeloma, prostate cancer, malignant melanoma, and non-melanoma skin cancer. The total sample size of site-specific cancer data ranged from 32,072 to 491,974.  We employed the following inclusion criteria for data selection: (1) When multiple datasets shared the same disease feature, we selected the dataset with the highest number of cases. If specific sample size for cases were unavailable in the current dataset, we chose the dataset with the largest overall sample size. (2) The GWAS cohort consisted entirely of individuals of European ancestry, and there was no overlap between the exposure and outcome cohorts. The details of included GWAS dataset is avialable on Table 1. |
|  | c) | Describe measurement, quality control and selection of genetic variants | 9, 10 | We have undertaken multiple steps to select the eligible genetic variants as IVs. First, single nucleotide polymorphisms (SNPs) influencing T1D were identified from openGWAS (n = 520,580). To satisfy the first MR assumption, we selected IVs associated with T1D at genome-wide statistical significance treshold (p < 5 × 10^−8^ ). 20 Subsquently, we retained only the independent SNPs by excluding those in linkage disequilibrium (LD) with an r2 value of 0.001 and a window size of 10,000 kilobase (kb). [21] Second, we computed F-statistics to assess the strength of the IVs on T1D ensuring they exceeded the minimal threshold value (F > 10) to reduce weak instrument bias. **[**22] For genetic variants not available in the outcome dataset, we used the LDproxy function in LD link (accessed on 20^th^ April, 2024) to select proxy SNPs (with an r^2^ > 0.8). [23]  Different types of sensitivity analysis were employed to assess possible violation of the MR assumptions. |
|  | d) | For each exposure, outcome, and other relevant variables, describe methods of assessment and diagnostic criteria for diseases | 5 | T1D: T1D diagnosis, insulin treatment within a year of diagnosis, no T2D diagnosis.  Cancer : Diagnosis was confirmed in all patients based on histopathology reports and clinical examination. |
|  | e) | Provide details of ethics committee approval and participant informed consent, if relevant |  | Not applicable |
| 5 | **Assumptions** | Explicitly state the three core IV assumptions for the main analysis (relevance, independence and exclusion restriction) as well assumptions for any additional or sensitivity analysis | 6, 7 | MR design was built based on three major assumptions: (1) A strong association of IVs with exposure (T1D); (2) No associations among IVs and potential confounding factors; (3) IV only affect outcomes through exposure. [_15_] |
| 6 | **Statistical methods: main analysis** | Describe statistical methods and statistics used |  |  |
|  | a) | Describe how quantitative variables were handled in the analyses (i.e., scale, units, model) | 12 | Using MR methods and OR |
|  | b) | Describe how genetic variants were handled in the analyses and, if applicable, how their weights were selected | 9 | 1. SNPs must reach genome-wide significance P < 5×10^−8^ 2. Clumping technique 3. Harmonise |
|  | c) | Describe the MR estimator (e.g. two-stage least squares, Wald ratio) and related statistics. Detail the included covariates and, in case of two-sample MR, whether the same covariate set was used for adjustment in the two samples | 14 | Three robust methods, namely MR-Egger, weighted median, and MR-PRESSO were utilized for sensitivity analyses to investigate potential pleiotropic effects that could bias the MR causal estimates. [29]  The second MR assumption requires no associations among IVs and potential confounding factors. In this study, we explored genetic variants (IVs) associated with common risk factors associated with cancer, such as body mass index (BMI), T2D, smoking, alcohol consumption and physical activity, using GWAS summary data. [6] |
|  | d) | Explain how missing data were addressed |  | N/A |
|  | e) | If applicable, indicate how multiple testing was addressed |  | N/A |
| 7 | **Assessment of assumptions** | Describe any methods or prior knowledge used to assess the assumptions or justify their validity | 14 | To satisfy the first MR assumption, we selected IVs associated with T1D at genome-wide statistical significance threshold (p < 5 × 10^−8^ ). To satisfy second MR assumption, we used the Ldlink, a web-based collection of bioinformatic modules to identify genetic variants associated with the outcome that might act as potential confounders. |
| 8 | **Sensitivity analyses and additional analyses** | Describe any sensitivity analyses or additional analyses performed (e.g. comparison of effect estimates from different approaches, independent replication, bias analytic techniques, validation of instruments, simulations) | 15 | We used IVWs and MR-Egger to generate Cochran's Q statistic to check for heterogeneity, which represents a possible violation of modeling assumptions. This study used the MR-Egger regression intercept examination to estimate the potential pleiotropy between exposure and outcome. A p value of < 0.05 represented the existence of pleiotropy. Once heterogeneity or horizontal pleiotropy was noteworthy, we used MR-Pleiotropy Residual Sum and Outlier (MR-PRESSO) to remove outlier SNPs. Moreover, we conducted “leave-one-out” test to determine whether a single SNP had a significant independent effect on MR estimates. |
| 9 | **Software and per-registration** |  |  |  |
|  | a) | Name statistical software and package(s), including version and settings used | 12 | Statistical analyses were conducted using packages such as “MendelianRandomization” (v.0.9.0), “TwoSampleMR” (v.0.5.10), “MRInstruments” (v.0.3.2), “MR-PRESSO” (v.1.0) in R (v.4.3.3). |
|  | b) | State whether the study protocol and details were pre-registered (as well as when and where) |  | N/A |
|  | **RESULTS** |  |  |  |
| 10 | **Descriptive data** |  |  |  |
|  | a) | Report the numbers of individuals at each stage of included studies and reasons for exclusion. Consider use of a flow diagram | 9 | Table 1 presents the characteristics of populations included in GWAS data on exposure and outcome. |
|  | b) | Report summary statistics for phenotypic exposure(s), outcome(s), and other relevant variables (e.g. means, SDs, proportions) | 9 | These traits are detailed in previous studies. |
|  | c) | If the data sources include meta-analyses of previous studies, provide the assessments of heterogeneity across these studies | 7 | We compiled individual-level genotype data and summary statistics from 18,942 individuals with T1D and 501,638 control individuals of European ancestry from public sources, where T1D case cohorts were matched to population control cohorts on the basis of country of origin where possible (Finland, Republic of Ireland, U.K.)). For non-UK Biobank cohorts, we first applied individual and variant exclusion lists (where available) to remove low-quality, duplicate, or non-European ancestry samples for each cohort. For control cohorts, we also used phenotype files (where available) to remove individuals with T2D or autoimmune diseases. |
|  | d) | For two-sample MR:  i.  Provide justification of the similarity of the genetic variant-exposure associations between the exposure and outcome samples  ii.  Provide information on the number of individuals who overlap between the exposure and outcome studies | 8 | The maximum sample overlapping rate between exposure data and outcome data is less than 1% |
| 11 | **Main results** |  |  |  |
|  | a) | Report the associations between genetic variant and exposure, and between genetic variant and outcome, preferably on an interpretable scale | 14 | A total of 89 SNPs were used in the MR analysis to genetically predict the causal relationship between T1D and 22 site-specific cancers. The F-statistics indicated no weak instrument bias in this MR analysis. For further information please see Supplementary Table 1. |
|  | b) | Report MR estimates of the relationship between exposure and outcome, and the measures of uncertainty from the MR analysis, on an interpretable scale, such as odds ratio or relative risk per SD difference | 16 | The IVW results of the MR analysis showed an evidence of causal associations of T1D with an increased risks of lung cancer (OR = 1.018, 95%CI: 1.004-1.033, *p* = 0.011), colorectal cancer (OR = 1.022, 95%CI: 1.003 - 1.041, *p* = 0.019), prostate cancer (OR = 1.018, 95%CI: 1.005-1.030, *p* = 0.006) and decreased risks of breast cancer (OR = 0.989, 95%CI: 0.981-0.998, p =0.016), lymphoma (OR = 0.999, 95%CI: 0.974 – 0.999, *p* = 0.003), malignant melanoma (OR = 0.999, 95%CI:0.989 - 0.999, *p* = 0.001), and non-melanoma skin cancer (OR = 0.999, 95%CI:0.899 - 0.999, *p* = 0.003). (Figure 3) |
|  | c) | If relevant, consider translating estimates of relative risk into absolute risk for a meaningful time period |  | Not relevant |
|  | d) | Consider plots to visualize results (e.g. forest plot, scatterplot of associations between genetic variants and outcome versus between genetic variants and exposure) | 16, 19 | Figure 3 presents forest plot of MR study investigating the causal effects of T1D on 22 site-specific cancers.  Figure 4 presents scatter plot illustrating the MR estimate of T1D effect on different types of cancer exhibited a distinct linear trend. |
| 12 | **Assessment of assumptions** |  |  |  |
|  | a) | Report the assessment of the validity of the assumptions | 17, 18 | Additionally, we conducted several sensitivity analyses to determine potential heterogeneity and horizontal pleiotropy (Table 2). |
|  | b) | Report any additional statistics (e.g., assessments of heterogeneity across genetic variants, such as *I^2^*, Q statistic or E-value) | 17 | Cochran's Q-test revealed no heterogeneity among the instruments associated with 16 types of cancer. No horizontal pleiotropy in the primary analysis |
| 13 | **Sensitivity analyses and additional analyses** |  |  |  |
|  | a) | Report any sensitivity analyses to assess the robustness of the main results to violations of the assumptions | 18 | The sensitivity test revealed consistent results with primary MR analysis. According to the MR-Egger interecept (*P*interecept > 0.05), the sensitivity analysis provided no evidence of horizontal pleiotropy across all analyses. (Table 2). |
|  | b) | Report results from other sensitivity analyses or additional analyses | 20 | Outlier SNPs were identified and corrected using MR-PRESSO. In addition, T1D-associated SNPs showed no associations with potential confounders. |
|  | c) | Report any assessment of direction of causal relationship (e.g., bidirectional MR) | 16 | The MR Steiger’s directionality test revealed no indications of reverse causality between T1D and all types of cancer, as shown in the Supplementary Table 25. |
|  | d) | When relevant, report and compare with estimates from non-MR analyses |  | N/A |
|  | e) | Consider additional plots to visualize results (e.g., leave-one-out analyses) | 20 | Furthermore, Leave-one-out analyses reaffirmed the reliability of the causal effects of individual instruments on the overall MR estimate, as illustrated in Supplementary Figures 1-22. |
|  | **Discussion** |  |  |  |
| 14 | **Key results** | Summarize key results with reference to study objectives | 22 | Our MR study aimed to assess this relationship, thereby expanding knowledge in the field of diabetes and cancer research. The result of this study found that T1D was associated with an increased risks of lung, colorectal, and prostate cancers as well as decreased risks of breast cancer, lymphoma, malignant melanoma, and non-melanoma skin cancer. |
| 15 | **Limitations** | Discuss limitations of the study, taking into account the validity of the IV assumptions, other sources of potential bias, and imprecision. Discuss both direction and magnitude of any potential bias and any efforts to address them | 26 | Firstly, the GWAS data utilized in this study predominantly comprised participants of European ancestry, limiting the generalizability of our findings to other populations. Secondly, some heterogeneity was observed in a few site-specific cancers. Nonetheless, robust MR methods less sensitive to heterogeneity, such as the weighted median method or MR-Egger, were employed to provide more reliable estimates even in heterogeneous conditions. Furthermore, a multiplicative random effect IVW method was utilized to mitigate the impact of heterogeneity. Thirdly, despite employing various quality control methods to address pleiotropy, the potential bias from unknown pleiotropic effects cannot be entirely eliminated. Finally, due to the lack of detailed information on variables such as age, sex, and T1D severity in summary-level data, subgroup analysis was not feasible. |
| 16 | **Interpretation** |  |  |  |
|  | a) | Meaning: Give a cautious overall interpretation of results in the context of their limitations and in comparison with other studies | 26 | Despite employing robust methods to address heterogeneity and pleiotropy, potential biases from unknown pleiotropic effects cannot be fully ruled out, urging careful interpretation of the results. |
|  | b) | Mechanism: Discuss underlying biological mechanisms that could drive a potential causal relationship between the investigated exposure and the outcome, and whether the gene-environment equivalence assumption is reasonable. Use causal language carefully, clarifying that IV estimates may provide causal effects only under certain assumptions | 25 | Autoimmune processes in T1D may modulate these mechanisms, contributing to a decreased incidence of certain cancers. Furthermore, the similarity in the reduction of melanoma risk in both diabetes types might be attributed to diabetes management, which often involves monitoring blood glucose levels and treatments that can influence cellular pathways linked to cancer development.  The increased risk observed in our study could be attributed to factors such as chronic inflammation, hormonal imbalances, and hyperglycemia. |
|  | c) | Clinical relevance: Discuss whether the results have clinical or public policy relevance, and to what extent they inform effect sizes of possible interventions | 25 | The identification of specific cancer types associated with T1D has important implications for clinical practice. Given T1D is a lifelong disease, healthcare providers should be aware of the potential increased risk of lung, colorectal, and prostate cancers among T1D patients, which may necessitate targeted screening and early detection strategies. |
| 17 | **Generalizability** | Discuss the generalizability of the study results (a) to other populations, (b) across other exposure periods/timings, and (c) across other levels of exposure | 26 | However, there are still some limitations that warrant attention. Firstly, the GWAS data utilized in this study predominantly comprised participants of European ancestry, limiting the generalizability of our findings to other populations. |
|  | **Other information** |  |  |  |
| 18 | **Funding** | Describe sources of funding and the role of funders in the present study and, if applicable, sources of funding for the databases and original study or studies on which the present study is based | 27 | This study was supported by the National Natural Science Foundation of China (82104596), the Shenzhen Key Medical Discipline Construction Fund & Sanming Project of Medicine in Shenzhen (SZSM202111002), the Medicine-Engineering Interdisciplinary Research Foundation of Shenzhen University (2023YG019), and the Shenzhen Science and Technology Program (GJHZ20220913143005010). |
| 19 | **Data and data sharing** | Provide the data used to perform all analyses or report where and how the data can be accessed, and reference these sources in the article. Provide the statistical code needed to reproduce the results in the article, or report whether the code is publicly accessible and if so, where | 27 | The dataset(s) supporting the conclusions of this article were obtained from IEU OpenGWAS (<https://gwas.mrcieu.ac.uk/>). |
| 20 | **Conflicts of Interest** | All authors should declare all potential conflicts of interest | 28 | The authors declare that they have no known competing financial interests or personal relationships that could have appeared to influence the work reported in this paper. |

This checklist is copyrighted by the Equator Network under the Creative Commons Attribution 3.0 Unported (CC BY 3.0) license.

1. Skrivankova VW, Richmond RC, Woolf BAR, Yarmolinsky J, Davies NM, Swanson SA, et al. Strengthening the Reporting of Observational Studies in Epidemiology using Mendelian Randomization (STROBE-MR) Statement. JAMA. 2021;under review.

2. Skrivankova VW, Richmond RC, Woolf BAR, Davies NM, Swanson SA, VanderWeele TJ, et al. Strengthening the Reporting of Observational Studies in Epidemiology using Mendelian Randomisation (STROBE-MR): Explanation and Elaboration. BMJ. 2021;375:n2233.
